# Probing Triplet Photochemistry in Ru(II) Porphyrins Using Singlet Oxygen Generation and Triplet–Triplet Annihilation Upconversion

**DOI:** 10.1021/acs.inorgchem.6c03345

**Published:** 2026-10-11

**Authors:** Aleksandra Nyga, Marli Ferreira, Piotr Pander, Ivan Gusev, Larissa Gomes Franca, Georgina H. Burgoyne Morris, Martyna Jankowska, Priscila Pazini Abatti, Karol Erfurt, Rachel C. Evans, Agata Blacha-Grzechnik

**Affiliations:** † SPIN-Lab Centre for Microscopic Research on Matter, University of Silesia in Katowice, 75 Pulku Piechoty 1A, Chorzow 41-500, Poland; ‡ Institute of Chemistry, University of Silesia in Katowice, Szkolna 9, 40-006 Katowice, Poland; § Faculty of Chemistry, 49569 Silesian University of Technology, Strzody 9, 44-100 Gliwice, Poland; ∥ Centre for Organic and Nanohybrid Electronics, Silesian University of Technology, Konarskiego 22b, 44-100 Gliwice, Poland; ⊥ Department of Physics, Durham University, South Road, Durham DH1 3LE, U.K.; # Department of Materials Science and Metallurgy, 2152 University of Cambridge, Cambridge CB3 0FS, U.K.; ∇ Department of Chemistry, Federal University of Santa Catarina, 88040-900 Florianópolis, Santa Catarina, Brazil

## Abstract

Porphyrins are known for their rich triplet-state photophysics, making them attractive platforms for light-driven applications. Compared with extensively studied Pd­(II) and Pt­(II) porphyrins, Ru­(II) porphyrins remain less systematically explored, particularly for triplet-driven reactivity. Here, we demonstrate that a single porphyrin framework can perform complementary functions: generate singlet oxygen (^1^O_2_) and photosensitize triplet–triplet annihilation upconversion (TTA-UC). We examine six related systems: free-base porphyrins (**1A**/**1B**) and Ru­(II) complexes **2A**/**2B** with CO and **3A**/**3B** with CO plus **PyPX** (10-(pyridin-4-yl)-10*H*-phenoxazine). Complexes **3A** and **3B** are newly synthesized Ru­(II) porphyrins, enabling direct assessment of axial ligation effects within this series. Optical spectroscopy, DFT/TD-DFT calculations, ^1^O_2_ measurements, and TTA-UC experiments investigate how metalation, bromination, and axial ligation influence triplet-state properties. Ru­(II) insertion promotes intersystem crossing, leading to ^1^O_2_ quantum yields up to 70%. Bromination further increases **Φ**_Δ_ in the free-base and monoaxial complexes. In contrast, PyPX coordination significantly increases **Φ**_Δ_ in the nonbrominated system (**2A** to **3A**) but has negligible influence in the brominated analogue (**2B** to **3B**). Complexes **3A**/**3B** also sensitize TTA-UC, achieving efficiencies of ∼1.3–1.4% with perylene in THF. Collectively, our results establish design principles for Ru­(II) porphyrins as triplet sensitizers, enabling control over complementary triplet-driven photochemical processes within a single molecular platform.

## Graphical abstract

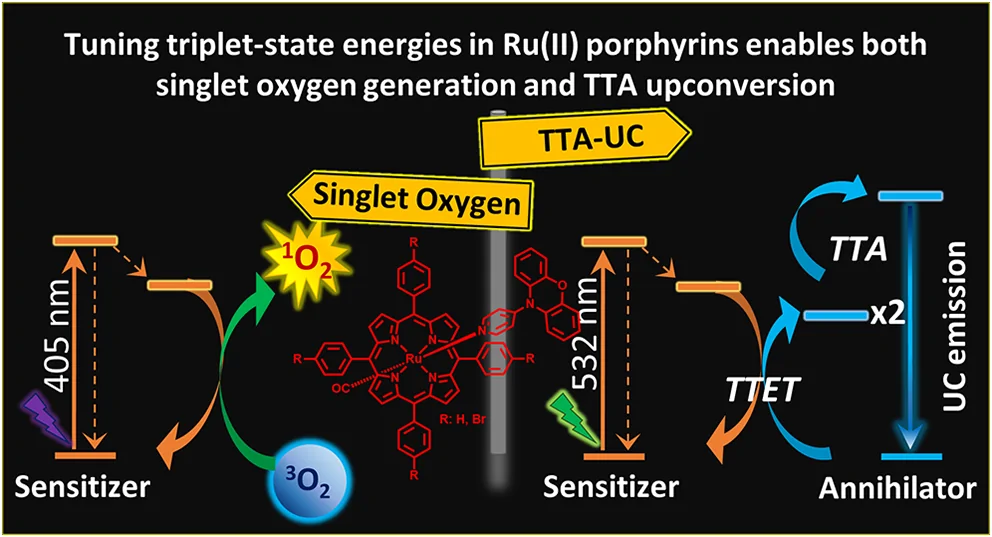

## 1 Introduction

Porphyrins provide a versatile platform for investigating structure–property relationships in photoactive coordination compounds due to their robust π-conjugated framework. Subtle variations in substitution pattern, symmetry, and metal coordination can greatly affect excited-state energies, intersystem crossing (ISC), and triplet-state dynamics,^1–5^ enabling broad tunability of optical and redox behavior.^6–12^

The triplet excited states of porphyrins can be utilized in different but related photochemical processes. Their photosensitizing ability enables the generation of singlet oxygen (^1^O_2_) via Dexter-type energy transfer to molecular oxygen (^3^O_2_). The resulting ^1^O_2_ is a short-lived, highly reactive species widely exploited in photodynamic therapy, antimicrobial treatments, and photocatalytic reactions.^5,13–22^ Conversely, these same triplet states can participate in triplet–triplet annihilation upconversion (TTA-UC), in which two triplet excitons undergo annihilation to form a higher-energy singlet state and anti-Stokes emission.^23^ Owing to these triplet-state processes, porphyrin systems have found applications in photovoltaics, photocatalysis, sensing, bioimaging, and drug delivery.^8,9,24–27^

Effective TTA-UC depends on ISC, triplet–triplet energy transfer, and bimolecular annihilation,^28,29^ requiring appropriate triplet yield, lifetime, and energy alignment.^30^ Similarly, ^1^O_2_ generation requires efficient triplet formation, a sufficiently long triplet lifetime, and energetic compatibility with ^3^O_2_.^31^ Thus, both processes rely largely on the same fundamental triplet-sensitizer properties, while differing in the identity of the triplet acceptor and the subsequent photophysical steps. Their combined investigation therefore provides complementary information on triplet-state formation, energy transfer, and bimolecular reactivity.

Although metal-centered (MC)^4^ and ligand-centered (LC)^32^ approaches for engineering triplet states are individually well established, their combined effects in Ru porphyrins remain insufficiently understood in terms of systematic structure–property relationships.^33,34^ Because both ^1^O_2_ sensitization and TTA-UC depend on efficient population of long-lived triplet excited states, strategies that improve ISC are especially important for developing effective photosensitizers for phototherapeutic and photon upconversion applications.^9,16^

Compared to Pt­(II) and Pd­(II) porphyrins,^35–38^ which have been widely employed as triplet sensitizers for TTA-UC, Ru­(II) porphyrins have been less explored, particularly for triplet-mediated photochemical applications.^9,33,39,40^ To the best of our knowledge, no previous study has simultaneously investigated ^1^O_2_ generation and TTA-UC in Ru­(II) carbonyl porphyrin frameworks, including Ru­(II)­(Por)­(CO) systems and their axially coordinated analogs.

Strategies to enhance ISC or tune triplet energies are often explored separately, despite the fact that metalation, peripheral substitution, and axial ligation can jointly influence LC/CT character, spin–orbit coupling (SOC), and nonradiative decay pathways.^2,4,32^ Metalation of the porphyrin core increases SOC and facilitates ISC through heavy-metal effects (HAE),^41–43^ while in Ru­(II) porphyrin complexes, the magnitude of this effect depends not only on the metal identity but also on its electronic coupling to the porphyrin π-system. Their octahedral coordination environment additionally enables axial ligation as a practical handle to tune the local electronic structure.^12,44,45^

Similarly, modification at the *meso*-positions of the porphyrin macrocycle provides an additional strategy to tune the photophysical properties. For instance, halogenation can modify the π-electron distribution while introducing additional SOC contributions, often associated with HAE, thereby impacting ISC efficiency and triplet-state dynamics.^46,47^ Despite the broad tunability offered by these structural modifications, their combined effects on triplet-state energies and bimolecular triplet reactivity in Ru­(II) porphyrins remain relatively underexplored.

In this study, we investigate a series of Ru­(II) porphyrins designed to probe the combined effects of metal coordination, bromination, and axial ligation within a common structural framework. We report the first Ru­(II) porphyrin complexes bearing the 10-(pyridin-4-yl)-10*H*-phenoxazine **(PyPX)** axial ligand to modulate the electronic environment at the metal center. By varying these structural components, we aim to understand how the coordination environment and substitution patterns influence triplet-state properties and the resulting photophysical behavior. To this end, we employ ^1^O_2_ generation and TTA-UC as complementary probes of the triplet manifold, supported by DFT/TD-DFT calculations. Our results demonstrate that axial ligation modulates triplet-driven reactivity in a substitution-dependent manner, underscoring the nonintuitive structure–property relationships in Ru­(II) porphyrins.

## 2 Experimental Section

### 2.1 Methods

Free-base porphyrins **1A** and **1B**, their Ru­(II) carbonyl complexes **2A** and **2B**, the axial ligand **PyPX**, and the corresponding axially coordinated complexes **3A** and **3B** were synthesized and characterized using NMR spectroscopy, attenuated total reflectance Fourier-transform infrared spectroscopy, and high-resolution mass spectrometry. The electrochemical and photophysical properties of the compounds were investigated using cyclic voltammetry, UV–Vis absorption spectroscopy, and steady-state and time-resolved photoluminescence spectroscopy. Singlet oxygen generation was evaluated in dichloromethane using 2,3,4,5-tetraphenylcyclopentadienone as a chemical trap, with quantum yields determined relative to 5,10,15,20-tetraphenylporphyrin under 405 nm excitation. Triplet–triplet annihilation upconversion measurements were performed in deaerated tetrahydrofuran using **3A** or **3B** as the sensitizer and 9,10-diphenylanthracene or perylene as the annihilator under 532 nm excitation. Full synthetic procedures, compound characterization data, experimental protocols, instrument parameters, and additional results are provided in the Supporting Information.

### 2.2 Computational Methods

Density functional theory and time-dependent density functional theory calculations were performed using ORCA 5.0.3. Ground-state geometries were optimized at the BP86/def2-TZVP/CPCM­(CH_2_Cl_2_) level with D3BJ dispersion correction. Excited-state energies and spin–orbit coupling properties were calculated using relativistically corrected ZORA-def2-TZVP basis sets. Full computational settings and additional results are provided in the Supporting Information.

## 3 Results and Discussion

### 3.1 Molecular Design, Synthesis, and Structural Characterization

To explore how metal coordination and peripheral modification affect the excited-state behavior of porphyrins, a modular series of Ru­(II) porphyrins based on a common macrocyclic structure was designed. This strategy allows systematic changes in structural parameters while keeping a consistent electronic core, enabling direct comparisons across the series.

The system incorporates three main modifications. First, introduction of a Ru­(II) center creates a coordination environment that improves SOC and provides access to the triplet state. Second, peripheral bromination alters the electronic structure of the π-system and adjusts spin–orbit interactions within the macrocycle. Finally, axial coordination with 10-(pyridin-4-yl)-10*H*-phenoxazine (**PyPX**) refines the local coordination environment at the metal center. Together, these variations enable investigation of how metal coordination, substitution patterns, and axial ligation impact triplet-state formation and dynamics in the porphyrin scaffold.

To access the required structural framework, the corresponding porphyrin precursors were prepared. 5,10,15,20-tetraphenylporphyrin (**1A**) and 5,10,15,20-tetrakis­(4′-bromophenyl)­porphyrin (**1B**) were synthesized using an iodine-catalyzed synthesis, with minor modifications to a literature procedure.^48^ This protocol was also compared with the Lindsey^49^ and Adler-Longo methods.^50,51^ The iodine-catalyzed approach offers several advantages, including the mild Lewis acidity of iodine, no requirement for microwave activation,^52^ and very short reaction times (10 min, Scheme 1, step (i)). It employs an inexpensive catalyst (I_2_, 10 mol %) and a straightforward workup, providing reasonably good yields (up to 20%) while allowing the use of undistilled solvents and reagents and higher reactant concentrations (50 mM) compared with the classical Lindsey method (10 mM). Oxidation was completed by addition of 2,3-dichloro-5,6-dicyano-1,4-benzoquinone (DDQ), followed by stirring for 30 min (Scheme 1, step (ii)). A detailed comparison of the methods and full synthetic procedures is provided in the Supporting Information (SI).

**Scheme 1:**
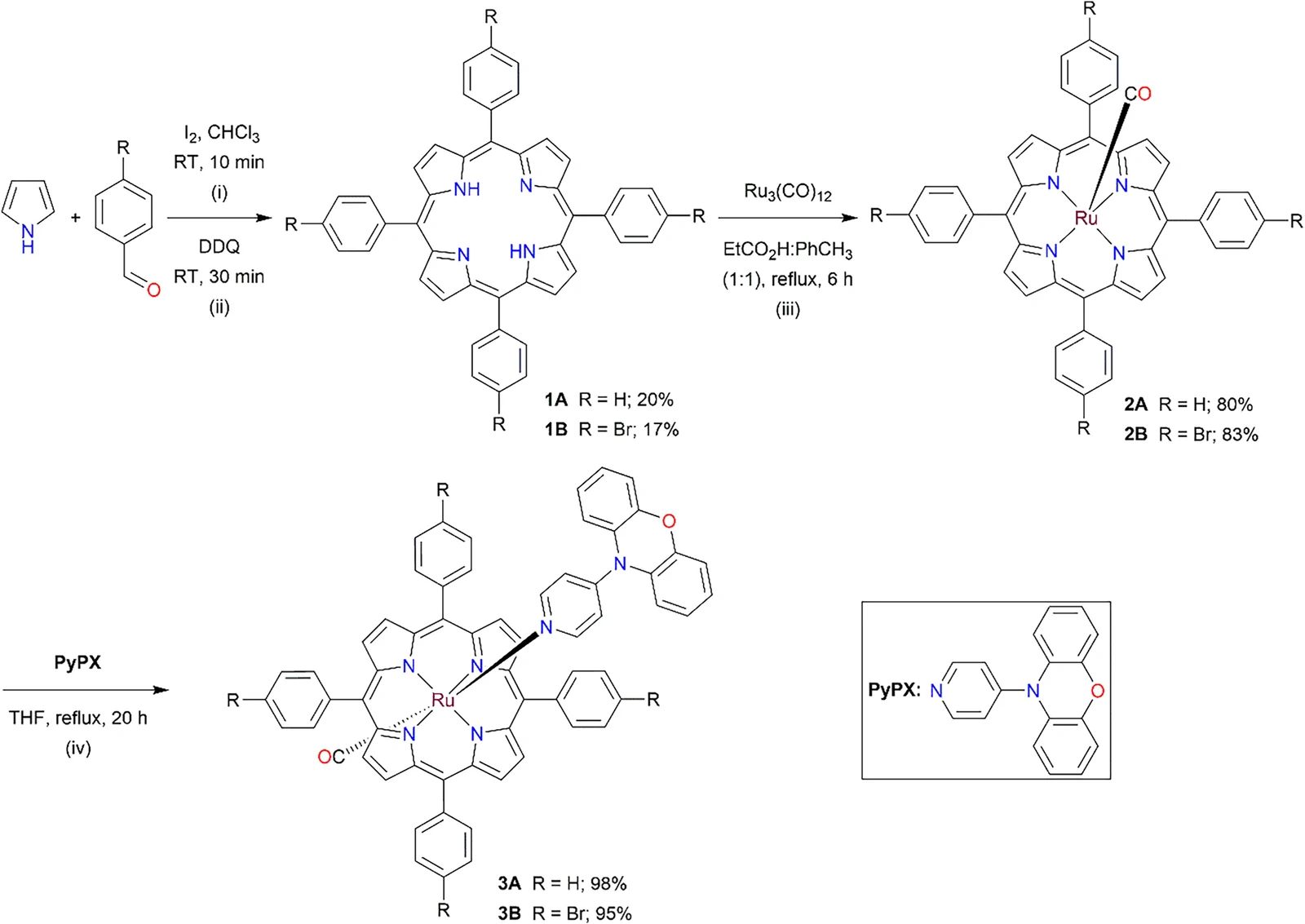
Synthesis of Free-Base Porphyrins and Their Ruthenium Complexes

Ruthenium insertion was investigated using triruthenium dodecacarbonyl (Ru_3_(CO)_12_) under reflux in an inert atmosphere, following conditions adapted from reported procedures.^53^ A range of solvents, including toluene,^53^ propionic acid,^54,55^ decalin^40^ and DMF,^56^ was screened over extended reaction times (up to 48 h), but these conditions proved largely ineffective, with only trace conversion observed by thin layer chromatography (TLC). Interestingly, a 1:1 mixture of toluene-propionic acid provided optimal conditions, yielding the target complexes in high efficiency (up to 83%) within ∼6 h (Scheme 1, step (iii)).

To further tune the triplet state properties, two Ru­(II) complexes were prepared via insertion of an axial **PyPX** ligand to the sixth vacant coordination position in the Ru­(II) porphyrin. The reaction between a given Ru­(II) porphyrin complex (**2A**/**2B**) and the **PyPX** ligand on refluxing in tetrahydrofuran leads to very good yields (up to 98%) of the corresponding biaxially coordinated Ru­(II) porphyrin complex (**3A**/**3B**, Scheme 1, step (iv)). The solubility of the target complexes was significantly increased relative to the Ru­(II) porphyrin precursors (**2A**/**2B**), likely due to the introduction of the bulky **PyPX** ligand, which reduces π–π stacking interactions between porphyrin cores, thereby facilitating dissolution and analysis. The use of pyridyl ligands in axial coordination with Ru­(II) porphyrins is well established, typically relying on solution-phase titration studies to generate the corresponding adducts and monitor supramolecular binding events.^44,57,58^ However, these systems are generally studied in situ and remain nonisolated. In contrast, we report here the first isolated and fully characterized PyPX-functionalized Ru­(II) porphyrin system.

The molecular structures of all target compounds were confirmed by Nuclear Magnetic Resonance spectroscopy (^1^H NMR, ^13^C­{^1^H} NMR), High Resolution Mass Spectrometry (HRMS), and Fourier-Transform Infrared Spectroscopy (FT-IR). Detailed synthetic procedures and spectroscopic data are provided in the Supporting Information.

The coordination of the **PyPX** ligand to the carbonyl-ruthenium complex (**2A**/**2B**, step (iv)) was confirmed by ^1^H NMR analysis. The coordinated ligand is strongly influenced by the diamagnetic current of the ruthenium porphyrin complex core, producing a marked upfield shift of its resonances. The protons closest to the porphyrin core in the 2- and 6-positions of the pyridine ring shift upfield by 7.30 ppm, while the 3- and 5-position protons shift upfield by 2.24 ppm (Figures S1 and S2). Integration of the proton signals from the **PyPX** coligand indicates the presence of only one such coligand in the axial position.

FTIR spectroscopy is another valuable technique for probing axial coordination in Ru­(II) porphyrin carbonyl complexes by monitoring changes in the C=O stretching frequency (highlighted region as ν­(CO)) (Figure S3). Coordination of the Lewis base nitrogen of **PyPX** to the ruthenium porphyrins is therefore indirectly confirmed through its electronic influence on the Ru–CO bond. The monoaxially coordinated Ru­(II) porphyrin complexes exhibit C=O stretching mode at 1945 cm^–1^ (**2A**) and 1968 cm^–1^ (**2B**), while the target complexes **3A** and **3B** show shifts to 1939 and 1954 cm^–1^, respectively. These shifts to lower energies are attributed to enhanced Ru → C=O backbonding (dπ → π*) facilitated by the additional electron-rich axial ligand (**PyPX**). This backdonation from the ligand to the metal center strengthens the Ru–C=O interaction, leading to the observed shift in the C=O stretching frequency. Similar shifts have been reported in other Ru­(Porphyrin)­(CO) complexes with N-containing ligands, such as pyridine (Py) or imidazole.^59^

### 3.2 Calculations

To understand the basic electronic properties of the porphyrin ligands and their associated ruthenium­(II) complexes, we used density functional theory (DFT) and time-dependent DFT (TD-DFT). Quasi-degenerate perturbation theory was used to model the phosphorescent properties of these complexes. The excited-state properties of the investigated compounds are summarized in Table 1.

**Table 1:** Summary of the Calculated Excited State Properties of Molecules **1A**/**1B**, **2A**/**2B**, and **3A**/**3B** at the B3LYP/ZORA-DEF2-TZVP Level of Theory for Non-Metal Atoms and B3LYP/SARC-ZORA-TZVP for the Ru Center^i^

			Δ*E*_1–3_ (ZFS)^c^		Δ*E*_1–4_ ^d^					
Compound	S_1_, eV^a^	T_1_, eV^b^	cm^–1^	meV	cm^–1^	meV	*k*_r_^1–3^,s^–^1^e^	*k*_r_^1–4^,s^–1^ ^f^	τ_1–3_,ms^g^	τ_1–4_,ms^h^
**1A**	2.13	1.58	0	0.0	702	87.1	∼0.1	-	∼10^4^	-
**1B**	2.12	1.57	0	0.0	742	92.0	4	-	223	-
**2A**	2.45	1.96	4	0.5	39	4.9	65	97	15	10
**2B**	2.45	1.97	10	1.2	17	2.1	41	51	25	20
**3A**	2.37	1.80	4	0.5	67	8.3	112	125	9	8
**3B**	2.29	1.81	5	0.6	67	8.3	131	161	8	6

a Lowest singlet state energy.

b Lowest triplet state energy.

c Energy difference between the lowest (1) and the highest (3) sublevels of the T_1_ state, also known as the zero-field splitting (ZFS).

d Energy splitting between the lowest sublevel of T_1_ and the lowest sublevel of T_2_.

e Triplet radiative decay rate considering only the T_1_ sublevels.

f Triplet radiative decay rate considering the T_1_ sublevel and the thermally accessible lowest T2 sublevel.

g Triplet decay lifetime assuming no nonradiative losses, considering only the T_1_ sublevels, τ_1–3_ = *k*_r_^1–3 −1^.

h Triplet decay lifetime assuming no nonradiative losses, considering the T_1_ sublevel and the thermally accessible lowest T_2_ sublevel, τ_1–4_ = kr^1–4 −1^. Details of the triplet radiative decay rate calculation can be found in the SI and in our earlier works.^60–62^

i Negligible ZFS amplitude.

Both porphyrin ligands **1A** and **1B** adopt a near planar geometry of the main macrocycle with the peripheral phenyl groups tilted out of the main plane. Similarly, their respective ruthenium­(II) complexes assume a fully planar configuration of the porphyrin ligand, with the carbonyl coligand assuming an axial position orthogonal to the main plane of the complex. Complexes decorated with the second coligand **PyPX** are coordinated in the second axial position, orthogonal to the main coordination plane of the complex.

Molecular orbitals (MOs) relevant to the two lowest excited states are shown in Figures 1 and 2. The respective MOs from the second-highest occupied molecular orbital (HOMO–1) to the second-lowest unoccupied molecular orbital (LUMO+1) in the ligands and complexes without the axial **PyPX** ligand display near identical symmetry, indicative of the mainly ligand-centered (LC) nature of these MOs in the Ru­(II) complexes. The **PyPX**-decorated complexes display a different HOMO than their parent counterparts, localized on the phenoxazine unit due to its electron-rich nature. However, the LUMO and LUMO+1, as well as the HOMO–1 and HOMO–2 orbitals, display similar symmetry to the analogous orbitals in the free-base porphyrin ligands.

**Figure 1:**
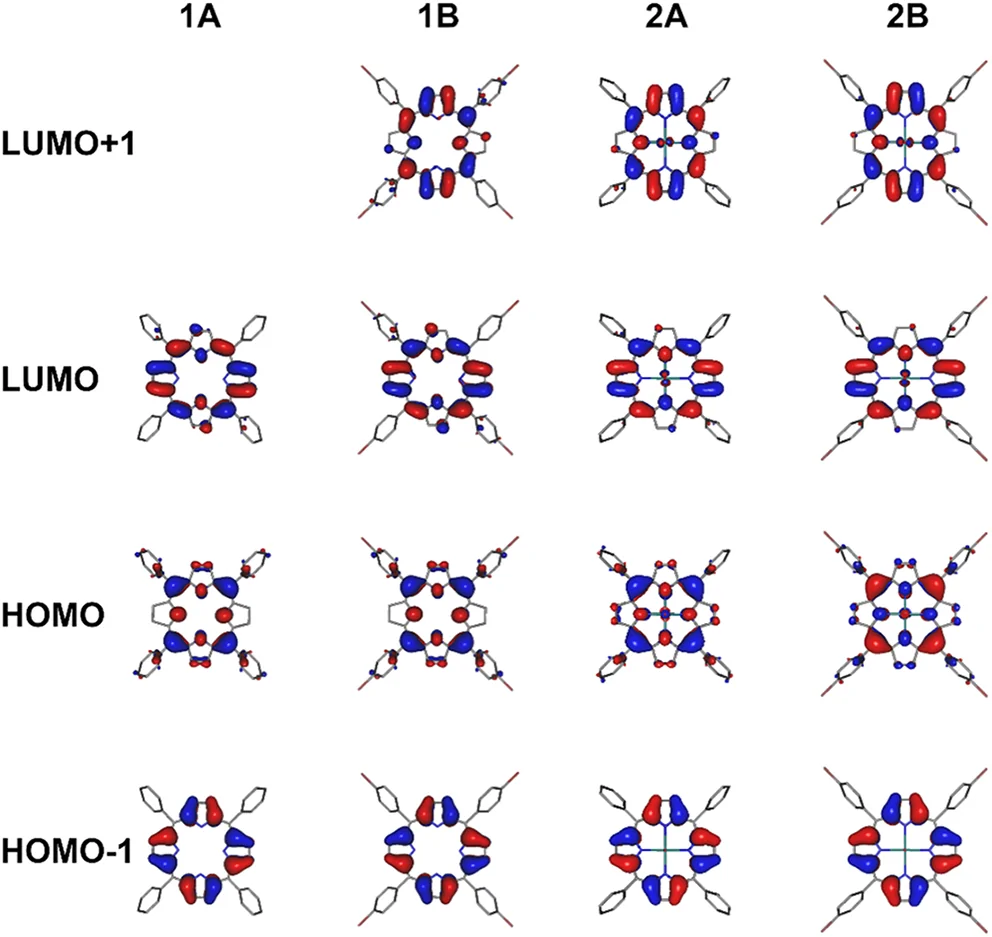
Molecular orbitals relevant to the two lowest singlet and triplet states in **1A**/**1B** and **2A**/**2B**. Iso value 0.03.

**Figure 2:**
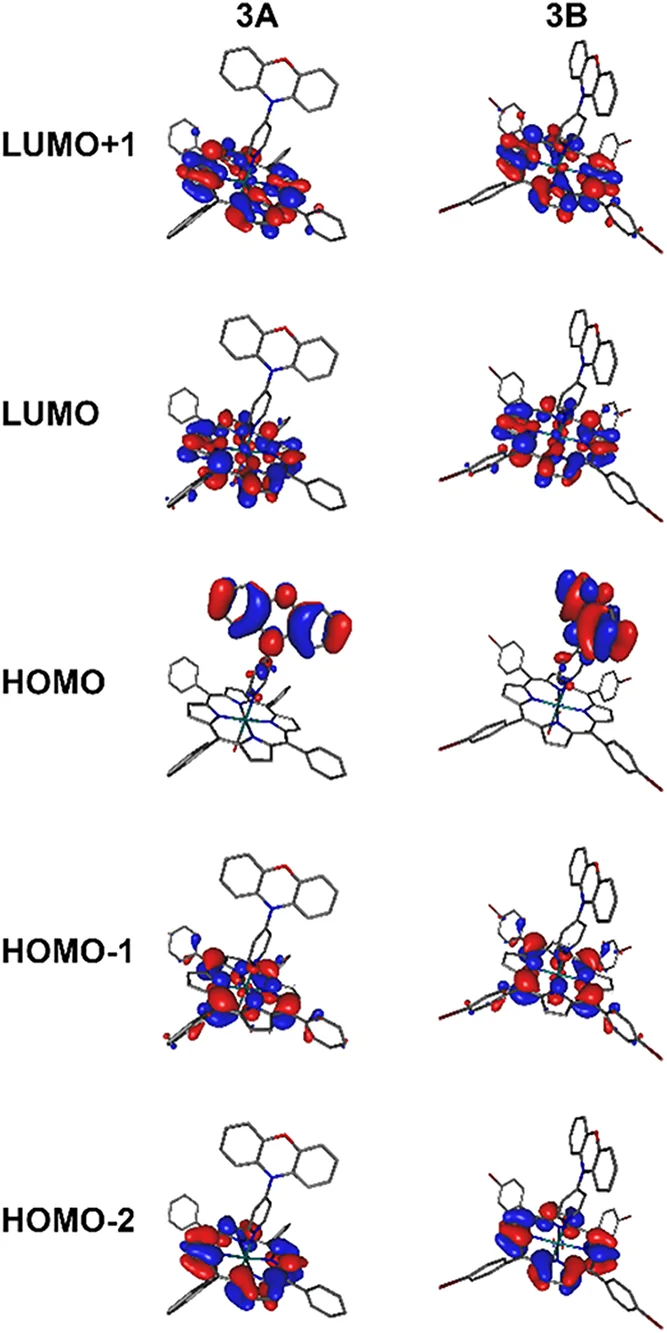
Molecular orbitals relevant to the lowest singlet and triplet states in **3A** and **3B**. Iso value 0.03.

In ligands **1A** and **1B**, the two lowest singlet excited states (S_1_ and S_2_) are separated by an energy difference Δ*E*_S2–S1_ ∼ 0.15 eV and dominated by HOMO → LUMO and HOMO–1 → LUMO+1, and HOMO → LUMO+1 and HOMO–1 → LUMO transitions, respectively. Similarly, the two lowest triplet excited states (T_1_ and T_2_) display an energy difference Δ*E*_T2‑T1_ = ∼0.09 eV and are dominated by HOMO → LUMO+1 and HOMO → LUMO transitions, respectively. Complexes **2A** and **2B**, with one axial carbonyl ligand, display degenerate S_1_ and S_2_ states (Δ*E*_S2–S1_ ∼ 0.001 eV) dominated by HOMO → LUMO and HOMO–1 → LUMO+1 excitations for S_1_, and HOMO–1 → LUMO and HOMO → LUMO+1 excitations for S_2_. The T_1_ and T_2_ states are also degenerate (Δ*E*_T2‑T1_ ∼ 0.001 eV), with the respective orbital pairings HOMO → LUMO and HOMO → LUMO+1. Hence, except for the degenerate nature of the two lowest singlet and triplet states, the orbital pairings in monoaxially substituted complexes **2A** and **2B** are analogous to those in the respective ligands **1A** and **1B**. The contribution of Ru­(II) orbitals to the excited state is minimal, resulting in their mostly LC nature.

Biaxially substituted complexes **3A** and **3B** feature a slightly different orbital pairing due to the presence of the axial **PyPX** ligand. Singlet excited states involving the HOMO (localized on **PyPX**) → LUMO (localized on the main porphyrin unit) or HOMO → LUMO+1 display charge-transfer (CT) character and a small oscillator strength, *f*_osc_(S_0_ → S*_n_*) ∼ 10^–5^–10^–4^, where *f*_osc_ denotes the transition probability between the ground singlet state S_0_ and an excited singlet state S*_n_*, due to the negligible orbital overlap. These weak transitions, therefore, are unlikely to stand out in the absorption spectrum of **3A** and **3B** and may be difficult to identify experimentally. Hence, the absorption spectra of **2A**/**3A** and **2B**/**3B** should be similar, as the other lowest singlet excited-states are of the same nature as in **2A** and **2B**. These CT states involving the HOMO → LUMO or HOMO → LUMO+1 S transition are near degenerate (Δ*E* ∼ 0.02 eV) and constitute the S_1_ and S_2_ states in **3B**. These states also constitute S_3_ and S_4_ states in **3A**; in both cases, they are energetically close (Δ*E* < 0.05 eV) to the other two lowest singlet states that display orbital pairing similar to that in **2A** and **2B**. States S_1_ and S_2_ in **3A** display orbital pairing HOMO–1 → LUMO and HOMO–1 → LUMO+1, respectively, while states S_3_ and S_4_ in **3B** display orbital pairing identical to respective states S_1_ and S_2_ in **3A**. Transitions involving these states display *f*_osc_(S_0_ → S*_n_*) ∼ 10^–2^, around two to three orders of magnitude larger than the CT transitions.

Analogous CT transitions were not found among the lowest four triplet excited states, indicating that the lowest triplet manifold is dominated by LC character, with negligible CT contribution. The T_1_ and T_2_ (Δ*E*_T2‑T1_ < 0.1 eV) display orbital pairing HOMO–1 → LUMO and HOMO–1 → LUMO+1, respectively. These are essentially the same orbital pairings as in the respective excited states in **2A** and **2B**. As these complexes essentially emit through their T_1_ states, this is consistent with their photoluminescence behavior, which is discussed in Sections 3.4.2.

Triplet states in metallorganic complexes split into three *quasi*-degenerate energy levels.^41^ The energy splitting between the first and third sublevels of the T_1_ state, also known as the zero-field splitting (ZFS), can be used as the overall measure of the amplitude of SOC.^41^ The triplet radiative rate can also act as a measure of SOC, as the two indicators are associated with each other. Considering these parameters, it is immediately apparent that the SOC strength is considerably weaker in the free-base porphyrin (**1A**/**1B**) than in the corresponding Ru­(II) porphyrin complexes (**2A**/**2B** and **3A**/**3B**), though we note that this is to be expected. The calculated triplet radiative rate in the bromine-decorated ligand **1B** is higher than in its parent molecule **1A**, highlighting the effect of a heavy halogen. All ruthenium­(II) complexes display modest splitting of the lowest triplet state (T_1_) due to the spin–orbit coupling-induced state mixing, with a ZFS < 10 cm^–1^, suggesting a rather low metal contribution and a mostly LC nature of the exited state. This is in line with the orbital iso surfaces displayed in Figures 1 and 2, which show a very small contribution of the Ru­(II) metal center to the excited state, with the electron density mainly localized over the porphyrin chelating ligand. Nevertheless, the effect of the Ru­(II) ion is orders of magnitude more significant than that of bromine, but the values of the ZFS ≈ 4–10 cm^–1^ are significantly lower than those reported in iridium­(III) or platinum­(II) complexes,^41^ which also correlates with overall larger triplet radiative rates of the latter two. For example, a typical platinum­(II) porphyrin complex displays a triplet lifetime in the range of 10–100 μs, while the calculated lifetimes of the presented Ru­(II) complexes are in the range of milliseconds (Table 1). These conclusions are in line with the findings reported earlier by some of the current authors, stating that the heavy metal effect associated with heavy halogens is relatively insignificant compared to that of the transition metal ions.^63^

We note an additional and interesting behavior of the lowest excited states in these porphyrin complexes, where the lowest sublevel of the T_2_ state (here denoted as state 4) is only a few meV above the lowest T_1_ sublevel (denoted as state 1). Consequently, the T_2_ state effectively also contributes to the phosphorescence radiative rate, even at temperatures as low as 77 K. Hence, the typical approach for calculating triplet radiative rates using only the 1–3 states^64^ appears insufficient in this case and we have included phosphorescence rates which consider the contribution of state 4, the lowest sublevel of T_2_. Thus, the obtained triplet radiative rates are up to 30% greater than those obtained by only including states 1–3 (Table 1).

We observe a clear trend between the four ruthenium­(II) complexes with the calculated radiative decay rate constant, *k*_r_, systematically larger for the **PyPX** decorated molecules (**3A**/**3B**). This suggests that the presence of the additional singlet CT state in these two complexes promotes slightly larger triplet radiative rates. On the other hand, the effect of the Br atoms on the radiative rates of Ru­(II) complexes is less systematic: bromination decreases *k*_r_ for **2A** → **2B**, but slightly increases it for **3A** → **3B**. This is likely due to the Ru­(II) metal center contributing significantly more to the overall SOC between S*_n_* and T*_n_* states than the halogen. The same does not apply to the free-base porphyrin (**1A**/**1B**), which in the absence of the Ru­(II) center, the relatively heavier Br atom induces now relevant HAE in the bromine-containing analogue.

In conclusion, the effects of bromination, metalation, and axial ligation on the triplet-state properties of these systems are interdependent rather than simply additive. In the free-base porphyrins, bromination increases the SOC between singlet and triplet states, leading to faster phosphorescence rates and more effective ISC through the HAE. However, in the Ru­(II) complexes, the SOC from the metal center already dominates the excited-state dynamics. As a result, bromination does not create a clear improvement in all triplet-state parameters. For instance, the calculated *k*_r_ drops for **2B** compared to **2A**, even with the presence of more heavy atoms. This behavior shows that the excited-state properties come from a delicate balance between SOC and changes in excited-state electronic character rather than from a simple additive HAE contribution. On the other hand, **PyPX** axially coordinated Ru­(II) complexes display higher radiative decay rate constants, even though this ligand has only a minor effect on the orbital geometry of the porphyrin-centered excited states ultimately responsible for triplet properties. We acknowledge though that this may simply be a computational artifact without a clear correlation. These results show that structural modifications in Ru­(II) porphyrins should not be viewed in isolation. Their influence on triplet-state energies and reactivity strongly depends on the overall electronic environment.

### 3.3 Electrochemical Characterization

To provide an experimental perspective on the computed electronic structure, the electrochemical behavior of the synthesized molecules was examined by cyclic voltammetry (CV). The HOMO and LUMO energy levels were estimated from the onset of the first oxidation and reduction curves, respectively (Table 2 and Figure 3). **1A** and **1B** undergo first and second reversible porphyrin π-ring centered oxidations.^65^ Furthermore, we observed two reversible reduction steps that can be assigned to the porphyrin π-ring centered reduction.^65^

**Figure 3:**
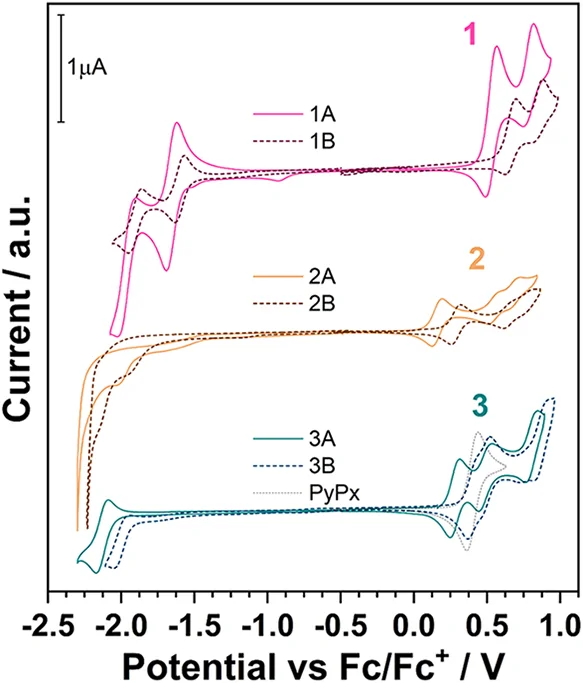
Cyclic voltammograms of porphyrins **1A**/**1B** and Ru­(II) complexes **2A**/**2B** and **3A**/**3B** in DCM. Derivatives decorated with bromine are shown in dashed lines. Oxidation of the **PyPX** ligand is shown as gray dashed line for the third (bottom) set of voltammograms.

**Table 2:** Selected Electrochemical Data and the Corresponding HOMO–LUMO Energy Levels for the Investigated Porphyrin Ligands and Ru­(II) Complexes^f^

Compound	*E*_onset, ox1_ ^a^	*E*_onset, red1_ ^b^	HOMO/eV^c^	LUMO/eV^d^	*E*_g_/eV^e^
**1A**	0.46	–1.58	–5.56	–3.52	2.04
**1B**	0.58	–1.53	–5.68	–3.57	2.11
**2A**	0.08	–1.91	–5.18	–3.19	1.99
**2B**	0.21	–1.77	–5.31	–3.33	1.98
**3A**	0.21	–2.06	–5.31	–3.04	2.27
**3B**	0.33	–1.92	–5.43	–3.18	2.25

a *E*_*o*nset_–onset potential.

b *E*_onset_–onset potential.

c HOMO–Highest Occupied Molecular Orbital.

d LUMO–Lowest Unoccupied Molecular Orbital.

e *E*_g_–energy gap.

f *c* = 0.5 mM analyte in 100 mM TBABF_4_ in DCM. Potentials are reported in V vs Fc/Fc^+^ redox couple. *E*_HOMO_ = −(*E*_onset, ox1_ + 5.1) [eV]; LUMO = −(*E*_onset, red1_ + 5.1) [eV].

The respective Ru­(II) complexes **2A** and **2B** display different electrochemical characteristics to the associated porphyrin ligands **1A** and **1B**. In this case, the redox potentials are shifted to lower values, which is due to the higher overall electron-donating ability of the Ru center and carbonyl axial ligand compared to the two protons in the free-base porphyrin.^66,67^ For both **2A** and **2B**, only a single irreversible reduction peak is observed in the potential window of DCM. These results are in line with the literature reports.^40^ A reversible reduction process is observed when CV analysis is performed in coordinating solvents such as DMSO, THF, DMF, PhCN, CH_3_CN, and pyridine.^55,68^ In this case, a solvent molecule is assumed to occupy the axial position of the complex, thereby stabilizing the reduced species.^66,67^

The axial **PyPX** ligand causes a shift of the first oxidation potential in **3A** and **3B** to more positive values by *ca*. 0.12 V with respect to **2A** and **2B**, similar to the literature-reported **RuCO-TPP-Py** complex with pyridine as axial ligand.^68^ However, the opposite behavior is observed for the reduction potential of **3A** and **3B**, which is shifted to more negative values by 0.15 V, resulting in a larger energy gap (Table 2). The difference in the reduction process of our target complexes can be attributed to the presence of the phenoxazine moiety, which is linked to pyridine at the *para*-position. The electron-donating phenoxazine helps the pyridyl nitrogen to form a stronger coordinate bond. It also increases the stability of the resulting complexes.

The *meso*-phenyls also affect the reduction and oxidation potentials. The oxidation potential increases as the electron-withdrawing capability of the substituent increases.^69^ In this way, both bromo-substituted ruthenium­(II) complexes (**2B**/**3B**) display oxidation potentials higher by ∼0.12 V compared to the nonbrominated parent complexes (**2A**/**3A**). An opposite effect is observed in the reduction process, where the potentials shift to more negative values by 0.14 V. Our results are in good agreement with those reported in the literature.^40^

The electrochemical energy gaps of the parent carbonyl porphyrin complexes are virtually the same, **2A** (1.99 eV) and **2B** (1.98 eV). Likewise, complexes with two axial ligands display similar gaps to each other, at **3A** (2.27 eV) and **3B** (2.25 eV), but larger than those of the former two. The largest difference in energy gap was observed between the two porphyrin ligands, where the recorded values are 2.04 and 2.11 eV for **1A** and **1B**, respectively. Additional bromines deepen the HOMO level from −5.56 eV (**1A**) to −5.68 eV (**1B**) while the effect on the LUMO energy is much less pronounced, from −3.52 to −3.57 eV, respectively in **1A** and **1B**.

The electrochemical trends are consistent with the electronic structures predicted by the DFT calculations. Specifically, the electron-rich **PyPX** ligand in **3A/3B** destabilizes the HOMO energy levels compared to **2A/2B**. Meanwhile, the LUMO levels stay mostly centered on the porphyrin, leading to larger electrochemical HOMO–LUMO gaps. This behavior aligns with the calculated frontier orbital distributions, which show a significant HOMO contribution from the **PyPX** ligand and the presence of low-lying charge-transfer excited states in the biaxially coordinated complexes.

### 3.4 Optical Spectroscopy

#### 3.4.1 UV–Vis Absorption and Photostability

Although the calculations as well as electrochemical analysis show only modest changes upon Ru­(II) insertion, bromination, and axial **PyPX** ligation, such structural modifications can still reshape the excited-state manifold. To elucidate these effects, we examine the UV–Vis absorption and the photostability of the complexes. The normalized absorption spectra of all complexes and their corresponding porphyrin ligands measured in DCM are shown in Figure 4. All compounds display a strong Soret band (λ_abs_ ≈ 412–418 nm), followed by weaker Q bands, as typical of porphyrins and their metal complexes.^6^ These bands are the result of mainly π–π* transitions, which often involve some d orbital contributions in Ru­(II) complexes.^6,40^ While the free-base porphyrins **1A** and **1B** exhibit four weak Q bands, only two Q bands are observed for Ru­(II) porphyrins **2A**, **2B**, **3A**, and **3B** (inset Figure 4). This reduction in Q-band number is consistent with literature reports,^40,45,55^ reflecting the perturbation caused by the Ru­(II) center’s d-electronic configuration, which induces strong mixing of orbital excitations and configurational interactions within the macrocycle. These effects are well described by Gouterman’s model.^6,7,70^

**Figure 4:**
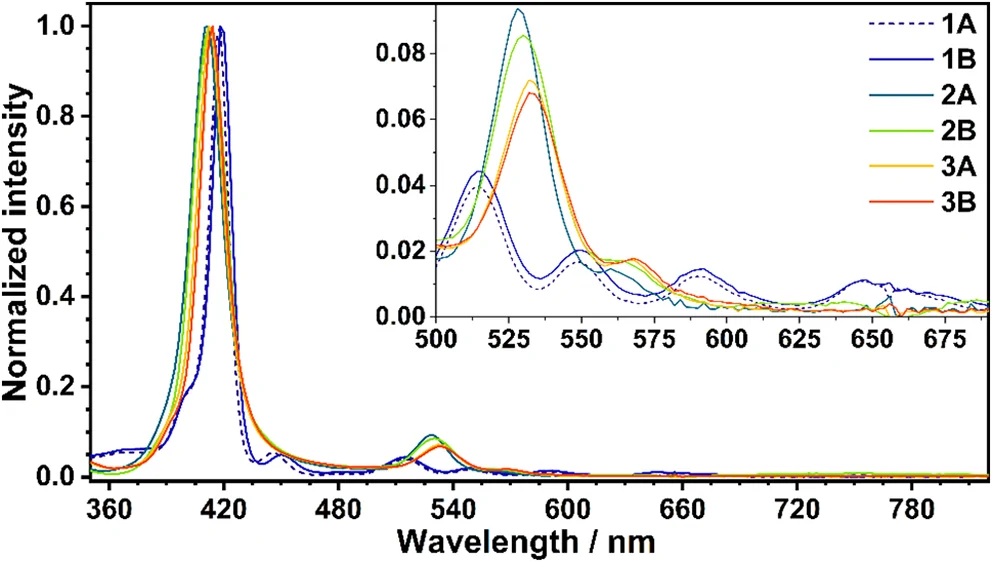
Normalized UV–Vis absorption spectra of **1A**/**1B** and Ru­(II) complexes **2A**/**2B** and **3A**/**3B** in CH_2_Cl_2_. Spectra are normalized to the Soret-band maximum; the inset highlights the Q-band region.

Photodegradation studies (Table 3) revealed significantly faster bleaching under broadband xenon lamp exposure (λ ≈ 240–1200 nm) than under monochromatic 405 nm excitation. Since singlet oxygen is generated through energy transfer from the triplet excited state of porphyrins, the observed photodegradation under aerobic conditions is expected to be largely mediated by ^1^O_2_-related oxidative pathways. For porphyrin photosensitizers, photobleaching does not necessarily scale monotonically with singlet oxygen photogeneration quantum yield, because oxygen can both participate in oxidative pathways and quench triplet states. Oxygen-dependent porphyrin photodegradation studies show that degassing conditions can markedly extend the triplet lifetime and thereby change the apparent photostability trends.^71,72^ Under broadband xenon-lamp irradiation, the samples are excited across both the Soret and Q-band regions, whereas 405 nm excitation addresses primarily the Soret band. This broader excitation window is consistent with the faster photobleaching observed under xenon-lamp irradiation in our experiments.

**Table 3:** Summary of the Optical Properties of the Tested Derivatives and Their Photodegradation^a^

Compound	λ_abs_ / nm	Photodegradation rate constant *k*_L405_ × 10^4^/mol·dm^–3^·s^–1^	Photodegradation rate constant *k*_LXe_ × 10^3^/mol·dm^–3^·s^–1^
**1A**	418	0.56	6.15
**1B**	418	0.053	2.89
**2A**	412	1.4	5.67
**2B**	412	3.5	2.77
**3A**	412	1.4	2.80
**3B**	414	2.9	2.00

a λ_abs_–Wavelength of the Soret absorption band which is simultaneously the strongest absorption band in the studied optical window; *k*_L405_ and *k*_LXe_–photodegradation rate constant during 405 nm laser and xenon-lamp irradiation, respectively.

#### 3.4.2 Photoluminescence

Photoluminescence (PL) characteristics were recorded in degassed 2-methyltetrahydrofuran (2-MeTHF) glass at 77 K using two freeze–pump–thaw cycles to remove dissolved oxygen (^3^O_2_), an efficient quencher of triplet excited states, due to the weak emission intensity of the Ru­(II) complexes at room temperature (Table 4 and Figure 5A). Upon Ru­(II) insertion, strong SOC is expected to promote efficient ISC and enable triplet emission (phosphorescence). We therefore recorded the spectra in degassed glassy 2-MeTHF to minimize oxygen quenching and to probe the intrinsic excited-state relaxation processes. Although ligands **1A** and **1B** display stronger fluorescence emission at room temperature than the respective Ru­(II) complexes, they were studied under the same low-temperature and degassed conditions as the complexes **2A**/**2B** and **3A**/**3B** to enable direct comparison before and after metalation. The PL spectra of all luminophores are well-resolved, due to their rigid structure, featuring three main vibronic bands, the third being significantly weaker than the other two. The emission of the complexes **2A**/**2B** and **3A**/**3B** is visibly red-shifted with respect to the ligands **1A** and **1B**, in line with similar behavior observed in other porphyrin complexes.^73,74^ The differences in the PL spectra between **1A** and **1B**, as well as within the **2A**/**2B** and **3A**/**3B** groups, are negligible, but the PL of the brominated variants (B series) is very slightly blue-shifted. Filling the empty second axial position with a pyridyl ligand (**PyPX**) does not appear to alter the PL spectra in any significant way, confirming the general conclusion from the computational study that emissive excited states generally originate from the porphyrin plane of the complex, while the electronic influence on the Ru­(II) center is negligible.

**Figure 5:**
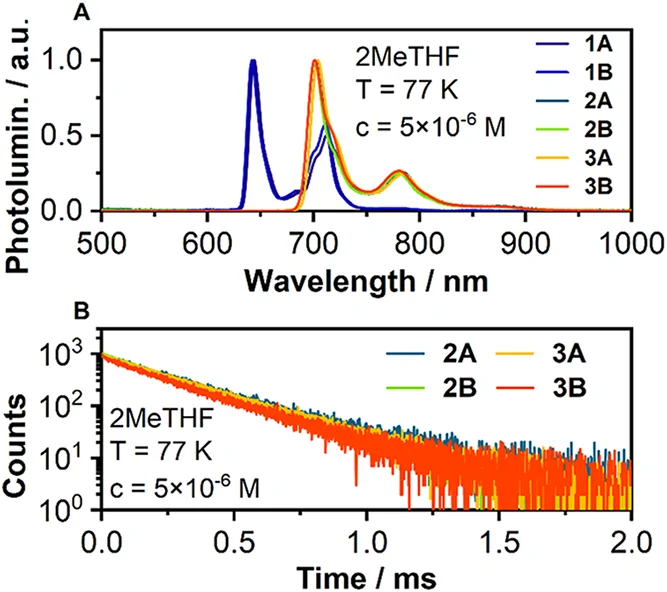
Photoluminescent characteristics of **1A**/**1B** to **3A**/**3B** in 2-MeTHF glass at 77 K. (A): Photoluminescence spectra; (B): Traces of photoluminescent decay for **2A**/**2B** and **3A**/**3B** only. λ_ex_ = 405 nm.

**Table 4:** Summary of Emission Characteristics of **1A**/**1B** to **3A**/**3B** in 2-MeTHF glass at 77 K, together with Singlet Oxygen Quantum Yields (Φ_Δ_ *)* Determined in DCM under 405 nm Laser Excitation Using TPCPD as^1^O_2_ Trap

Compound	λ_em_ (77 K), nm^a^	T_1_, eV^b^	τ (77 K), μs^c^	Φ_Δ_, %^d^
**1A**	644, 684, 702sh, 713, 785	-	-	47^e^
**1B**	641, 683, 699sh, 710, 783	-	-	54 ± 8
**2A**	703, 724sh, 783, 878	1.79	296	58 ± 7
**2B**	701, 719sh, 780, 874	1.80	252	69 ± 2
**3A**	704, 724sh, 785, 879	1.79	280	70 ± 6
**3B**	701, 718sh, 780, 873	1.80	258	69 ± 5

a PL maxima, sh – shoulder.

b Triplet energy calculated from the PL onset.

c Photoluminescence decay lifetime.

d Quantum yield of singlet oxygen photogeneration.

e The quantum yield (Φ_Δ_) was calculated using tetraphenylporphyrin (1A) as a reference via the standard comparative method with refractive-index correction. The Φ_Δ_ of 1A in DCM was obtained from measurements in CHCl_3_ and recalculated using $\phi_{DCM}=\phi_{CHCl_{3}}\cdot \frac{K_{DCM}}{K_{CHCl_{3}}}\cdot \frac{I_{CHCl_{3}}}{I_{DCM}}\cdot \frac{n_{DCM}}{n_{CHCl_{3}}}$ ,^75^ with the literature value Φ _CHCl3_ = 55%.^1^

Ligands **1A** and **1B** display fluorescence at 77 K, and we were unable to detect any non-negligible signals originating from their triplet emission. Unlike the ligands, the respective Ru­(II) complexes display long-lived emission (τ = 252–296 μs) originating from their triplet state, a consequence of the significant SOC introduced through the transition metal ion (Figure 5B). As we do not observe any detectable fluorescence from these complexes, one can assume near 100% efficiency of intersystem crossing, leading to significant populations of relatively long-lived triplet states being produced upon photoexcitation. Calculations indicate photoluminescence lifetimes 1–2 orders of magnitude longer than those observed experimentally (τ ∼ 9–25 ms vs τ ∼ 252–296 μs for complexes **2A**–**3B**, Tables 1 and 4), indicating that additional nonradiative decay channels remain active even at 77 K. This discrepancy most likely arises from residual aggregation or microheterogeneity in the frozen glass matrix, despite the use of dilute solutions (5 μM). The moderate emission intensities observed (Figure 5B) further suggest low radiative transition probabilities for the T_1_ → S_0_ transition, consistent with the small computed oscillator strengths. Nevertheless, one can note a systematic difference in the PL lifetime between brominated complexes **2B** and **3B**, τ ∼ 252–258 μs, and the parent nonhalogenated complexes **2A** and **3A**, τ ∼ 280–296 μs, which could be explained by the Br atoms accelerating the radiative, nonradiative, or both decay pathways. As in the case of the PL spectra, the PL lifetimes of complexes **3A** and **3B** with saturated axial positions behave nearly identically to their analogs **2A** and **2B** (Figure 5B), indicating that axial **PyPX** coordination has only a minor influence on triplet-state emission dynamics, in agreement with the calculated electronic structure, which predicts minimal perturbation of the porphyrin-centered excited states upon axial ligation.

### 3.5 Singlet Oxygen Photogeneration

The photosensitizing properties of the investigated molecules were assessed based on their ability to generate singlet oxygen using tetraphenylcyclopentadienone (TPCPD) as a selective ^1^O_2_ trap. Quantitative singlet-oxygen quantum yields (Φ_Δ_) were determined under monochromatic 405 nm laser excitation,^10,15,76–78^ which primarily excites the porphyrin macrocycle (Figures 6A and S5). Under these conditions, the contribution of the axial **PyPX** ligand depends on the substitution pattern: it enhances ^1^O_2_ generation in the nonbrominated complex **3A** compared to **2A**, but has a negligible effect in the brominated series (**3B** vs **2B**).

**Figure 6:**
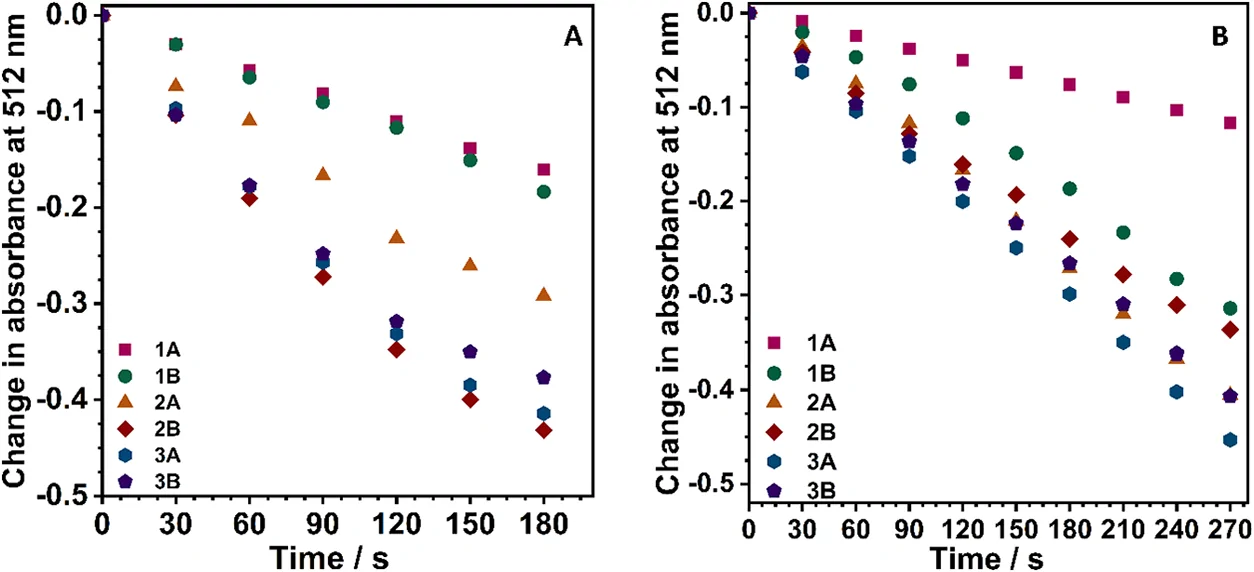
Changes in TPCPD absorbance at 512 nm over time for the ^1^O_2_–mediated photooxidation sensitized by the porphyrin derivatives in DCM. Singlet oxygen was generated by irradiating the sensitizers with (A) a 405 nm laser or (B) a xenon lamp.

In separate photooxidation experiments, broadband xenon lamp was used to compare the apparent TPCPD photooxidation kinetics. Under broadband illumination, the number of absorbed photons changes continuously during the experiment due to photobleaching (Table 3),^71^ which invalidates the linear assumptions required for reliable determination of absolute Φ_Δ_ values. Therefore, these data are reported only as comparative kinetics. In this case, **3A** shows the fastest apparent ^1^O_2_ production, as evidenced by the largest time-dependent decrease in the 512 nm TPCPD absorption band (Figure 6B).

Analysis of structure–property relationships related to ^1^O_2_ photogeneration reveals several trends:

#### 3.5.1 Metal Insertion

Introduction of Ru­(II) as the central ion (**1A** → **2A** and **1B** → **2B**) significantly improved the ^1^O_2_ photogeneration under both excitation conditions (Figure 6). This enhancement is attributed to the heavy-atom effect (HAE), which promotes faster ISC (S_1_ → T_1_) and increases triplet-state population.^57,79^

#### 3.5.2 Bromination

Under selective 405 nm excitation (Figure 6A), bromination leads to a slight increase in Φ_Δ_ for the free-base porphyrins (**1A** → **1B**), while a more pronounced increase is observed for the Ru­(II)-containing systems (**2A** → **2B**). This behavior is consistent with the calculated SOC effects of bromination, although their contribution to ^1^O_2_ photogeneration depends on the molecular framework.

#### 3.5.3 Axial Coordination (PyPX)

In the nonbrominated series, **3A** shows a noticeable increase in Φ_Δ_ compared to **2A** (Table 4), indicating that axial **PyPX** coordination enhances ^1^O_2_ generation. This effect may be attributed not only to reduced nonradiative decay but also to the introduction of an additional singlet charge-transfer state, as suggested by the theoretical calculations, which may promote stronger mixing of excited states and slightly increase the triple–triplet radiative rates. Although the calculations indicate some charge-transfer character associated with the additional singlet state, no direct experimental evidence for an intramolecular electron-transfer process was obtained. Together, these effects may improve the efficiency of triplet-state utilization for oxygen quenching. In the brominated series, the effect of the axial ligand is negligible (**3B** vs **2B**), as the HAE dominates. Under broadband xenon illumination (Figure 6B), **3A** also shows the fastest apparent Φ_Δ_ generation, suggesting that the axial **PyPX** ligand contributes additional energy transfer pathways when the system absorbs across a wider spectral range.

The Φ_Δ_ values reach up to 70%, demonstrating efficient singlet oxygen generation across the series. These trends are consistent with the theoretical calculations of the excited-state properties and measured emission behavior: bromine enhances SOC between singlet and triplet states, promoting faster ISC and phosphorescence, as reflected in the increased Φ_Δ_ values for the brominated derivatives (e.g., **2B** vs **2A**). At the same time, the presence of Ru­(II) already provides strong SOC, so in the fully substituted systems (**3A**/**3B**) the effect of bromination becomes less pronounced. The axial **PyPX** ligand slightly increases the radiative rates in the Ru­(II) complexes, consistent with the observed rise in Φ_Δ_ from **2A** to **3A**. Importantly, the low-temperature phosphorescence lifetimes should not be directly correlated with Φ_Δ_, as these parameters were determined under different experimental conditions. These trends are also in line with the observed photodegradation behavior (Table 3), suggesting that structural modifications affecting triplet-state formation and relaxation may contribute to both ^1^O_2_ photogeneration and photostability in this series.

### 3.6 Triplet–Triplet Annihilation Upconversion (TTA-UC)

After evaluating the photosensitizing properties via singlet oxygen generation, complexes **3A** and **3B** were selected for TTA-UC studies due to their efficient formation of long-lived triplet states and suitable triplet energy alignment for triplet–triplet energy transfer. To enable efficient triplet–triplet energy transfer (TTET) between the sensitizers and the annihilator, it is necessary to align the sensitizer and annihilator triplet energies.^12^ Diphenylanthracene (DPA) and perylene are among the most extensively studied and widely employed annihilators in TTA-UC systems, with their TTA-UC behavior being well established in the literature^80,81^ with triplet excited states (T_1_) of 1.77 eV^8^ and 1.53 eV,^9^ respectively. These benchmark annihilators were selected to provide an established TTA-UC platform for assessing the suitability of the newly synthesized Ru­(II) complexes **3A** and **3B** as photosensitizers. Comparing the triplet excited state energies of DPA and perylene with **3A** and **3B**, we observed that the T_1_ energies of the Ru­(II) porphyrins were slightly higher than that of DPA, and significantly higher than that of perylene (see Figure 7B). Figure 7A compares the absorption and emission spectra of DPA and perylene in THF solution for reference, along with their respective molecular structures.

**Figure 7:**
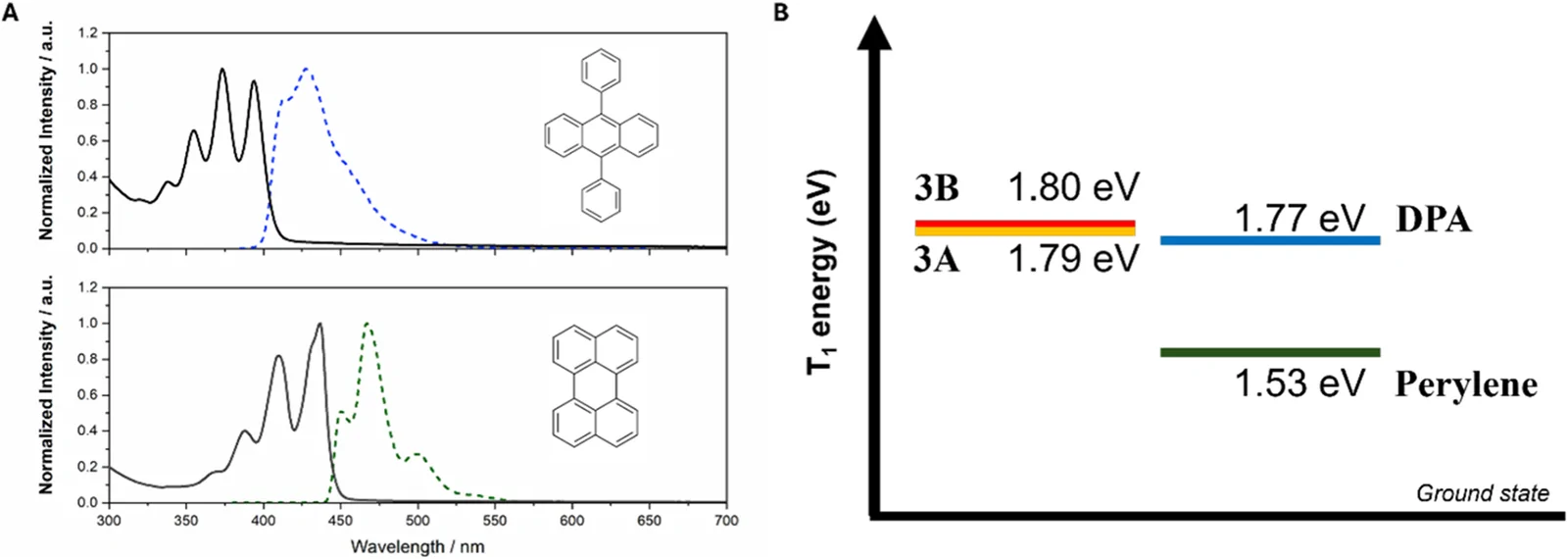
(A): Normalized absorption (solid lines) and emission spectra (dashed lines) of DPA (top panel) and perylene (bottom panel) in THF solutions (400 μM) and their respective molecular structures. λ_ex_ = 375 nm. (B): Comparison of T_1_ energy levels of the sensitizers, **3A** and **3B**, with the annihilators DPA and perylene. T_1_ energy level values were obtained from the references.^8,9^

The Ru­(II) porphyrins combined with DPA showed significantly lower upconversion quantum yield (Φ_UC_) values, around 0.2%, compared to around 1.3% with perylene (Figure 8), measured in THF solution at room temperature (sensitizer/annihilator = 400:10 μM) under 532 nm laser excitation at a maximum intensity of 100 W cm^–2^. This result is somewhat expected, as the greater energy difference between the triplet sensitizers and annihilators can lead to a more favorable TTET.^9^ On the other hand, the similar T_1_ energies between the **3A** and **3B** complexes and DPA allow them to remain in thermal equilibrium, leading the TTET and phosphorescence decay pathways to compete with each other. This is illustrated in Figure S7, where the DPA:**3A** and DPA:**3B** exhibit a stronger Ph contribution compared to Perylene:**3A** and Perylene:**3B**, which exhibit minimal phosphorescence. When comparing the two Ru (II) porphyrins, **3A** demonstrated slightly higher Φ_UC_ values with both annihilators. This can be attributed to the longer natural phosphorescence lifetimes of **3A** (τ ∼ 280 μs), which allows TTET to outcompete the phosphorescence decay pathway, likely leading to a higher triplet population in the annihilator and, consequently, enhanced upconversion. In contrast, for **3B**, the shorter natural Ph lifetime (τ ∼ 258 μs) might lead to a larger contribution of the phosphorescence decay pathway relative to TTET. The phosphorescence lifetimes reported for **3A** and **3B** were measured at low temperature (Table 4), as room temperature phosphorescence was too weak for reliable analysis. Therefore, quantitative triplet energy-transfer efficiencies could not be determined under the TTA-UC experimental conditions.

**Figure 8:**
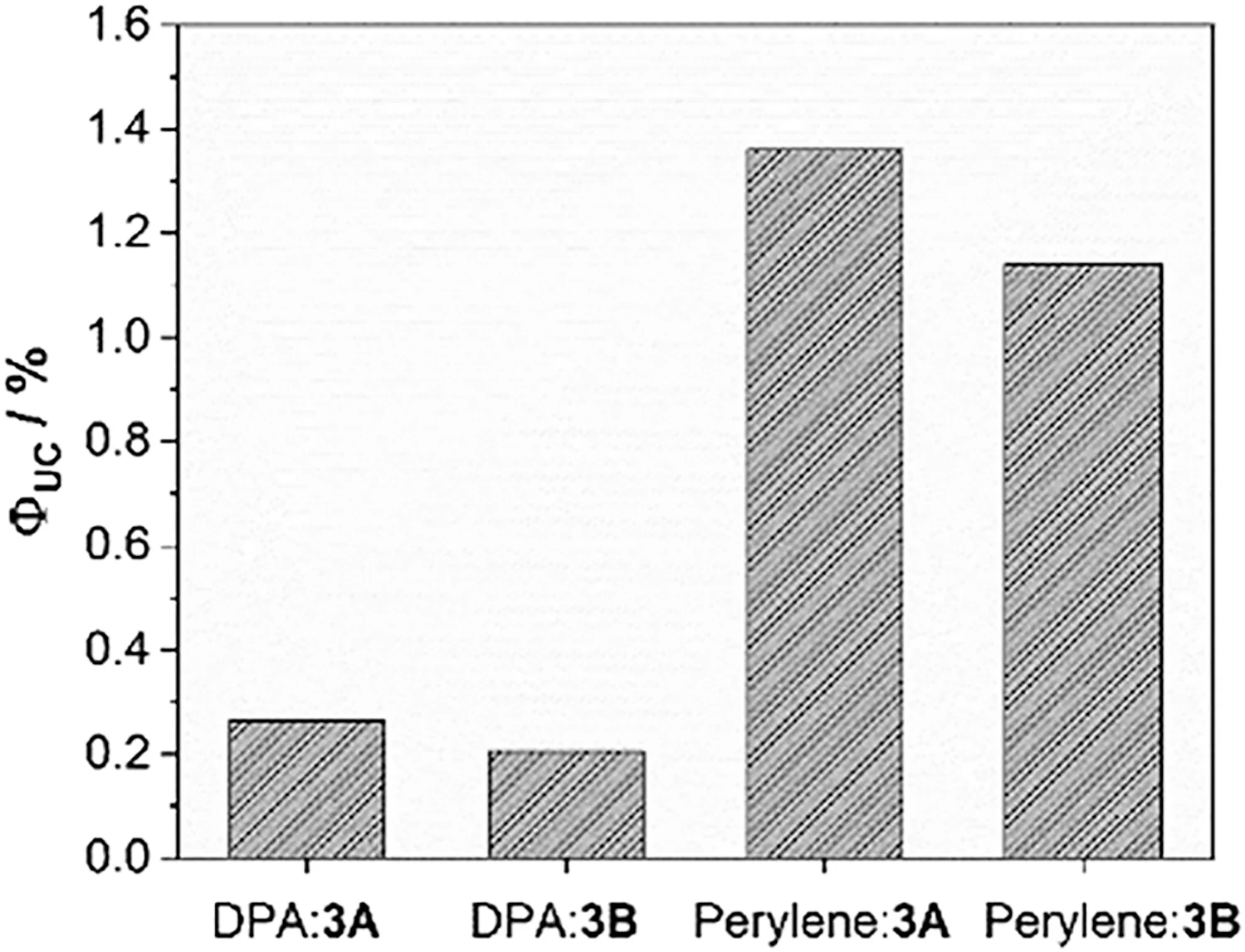
Upconversion quantum yield (Φ_UC_) of the TTA-UC pair in THF solutions at room temperature (400:10 μM) using **3A** and **3B** complexes as sensitizers, and DPA and perylene as annihilators. Measurement was performed using 532 nm excitation source at maximum laser intensity of 100 W cm^–2^.

Table 5 summarizes the decay lifetimes associated with upconversion and phosphorescence obtained from biexponential and triexponential tail fitting. The corresponding decay traces and fitted curves are provided in the SI (Figure S8).

**Table 5:** Summary of TTA-UC Kinetic Studies Using **3A** and **3B** Complexes as Sensitizers and DPA and Perylene as Annihilators in THF Solutions (400:10 μM) at Room Temperature

	^a^UC lifetimes				^b^Phosphorescence lifetimes				
TTA-UC systems	τ_1_, μs/f_1_, %	τ_2_, μs/f_2_, %	⟨τ_UC_⟩,μs	χ^2^	τ_1_, μs/f_1_, %	τ_2_, μs/f_2_, %	τ_3_, μs/f_3_, %	⟨τ_Ph_⟩, μs	χ^2^
DPA:**3A**	100/46	250/54	181	1.235	8.9/23	78/29	330/48	183	1.199
DPA:**3B**	25/97	380/3	44	1.389	7.9/66	52/34	-	23	1.168
Perylene:**3A**	39/81	70/19	37	1.234	2.6/3	25/56	59/41	38	1.246
Perylene:**3B**	16/36	28/64	24	1.156	^c^	^c^	^c^	^c^	^c^

a Lifetime fitting data for UC decay curves (average lifetime (⟨τ_UC_⟩), individual lifetimes (*t_i_*) and fractional contributions (*f_i_*) and goodness-of-fit (χ^2^). λ_ex_ = 532 nm, λ_em_ = 440 nm for DPA:**3A**and DPA:**3B**, λ_em_ = 470 nm for Perylene:**3A** and Perylene:**3B**

b Lifetime fitting data for phosphorescence decay curves (average lifetime (⟨τ_Ph_⟩), individual lifetimes (*t_i_*) and fractional contributions (*f_i_*) and goodness-of-fit (χ^2^). λ_ex_ = 532 nm, λ_em_ = 720 nm.

c There is no detectable signal.

The average UC lifetimes (⟨τ_UC_⟩) decrease when DPA is replaced by perylene as annihilator, with ⟨τ_UC_⟩ changing from ∼181 μs for DPA:**3A** to ∼37 μs for perylene:**3A**. This is consistent with the larger energy difference between the triplet sensitizer and the annihilator, which enables faster population of the annihilator triplet state. In addition, system using **3B** as sensitizer show significantly shorter ⟨τ_UC_⟩ and ⟨τ_Ph_⟩ lifetime values than its natural phosphorescence lifetime (τ ∼ 258 μs). The observation of this effect even in the DPA:**3B**, where triplet energy alignment is less favorable, indicates the presence of additional quenching pathways that reduce Φ_UC_ values. In related Ru­(II) porphyrin systems, Gray et al.^12^ reported TTA-UC quantum yields up to 4.5% in toluene with DPA, associated with significantly extended triplet lifetimes upon axial coordination. While a direct quantitative comparison is not possible due to differences in solvent, annihilator, and excitation conditions, our Φ_UC_ values (with perylene as annihilator) are within the same general range of efficiency, indicating that axial **PyPX** coordination provides a useful means of tuning triplet-mediated energy transfer in these systems.

## 4 Conclusions

In this work, we systematically investigated free-base porphyrins (**1A/1B**) and Ru­(II) porphyrins (**2A/2B** and the newly synthesized PyPX-bearing complexes **3A/3B**) to probe triplet-driven photochemistry. Using singlet-oxygen generation and triplet–triplet annihilation upconversion as complementary readouts, we show how metalation, bromination and axial ligation reshape the triplet manifold. All compounds exhibited high singlet-oxygen quantum yields (Φ_Δ_), reaching up to 70% and surpassing the reference sensitizer (Φ_Δ_ = 47%). The axial **PyPX** ligand in **3A** contributed to an increase in Φ_Δ_ compared to **2A**, highlighting a substitution-dependent effect of axial ligation on triplet-driven reactivity. In contrast, the same axial ligation has little effect in the brominated pair (**3B** vs **2B**). Complexes **3A** and **3B** were further examined for triplet–triplet annihilation upconversion experiments, affording quantum yields (Φ_UC_) of up to 1.3%, consistent with efficient triplet population and favorable triplet lifetimes, underscoring their dual triplet-sensitizing function. Overall, these results establish design guidelines for Ru­(II) porphyrins where targeted structural changes can steer competing bimolecular triplet pathways within a single molecular platform.

## Supplementary Material



## Data Availability

The data supporting the findings of this study are available within the article and its SI.

## Notes

Additional experimental details, materials and methods, synthetic procedures and compound characterization, computational methods, electrochemical and photophysical measurements, singlet oxygen photogeneration and triplet–triplet annihilation upconversion methods, supplementary spectroscopic and kinetic data, and NMR and high-resolution mass spectra of intermediates and final compounds (PDF)
