# Supplementary material for: Probing Triplet Photochemistry in Ru(II) Porphyrins Using Singlet Oxygen Generation and Triplet–Triplet Annihilation Upconversion
Source: Inorg Chem. 2026 Oct 11;65(42):24731–44. doi: 10.1021/acs.inorgchem.6c03345 (PMC13715520; doi:10.1021/acs.inorgchem.6c03345)
Supplement: Supplementary file 1 [file ic6c03345_si_001.pdf]

Supporting Information

Probing Triplet Photochemistry in Ru(II) Porphyrins using Singlet Oxygen Generation and Triplet-Triplet Annihilation Upconversion

Aleksandra Nyga,*[a,b,c] Marli Ferreira,*[d] Piotr Pander,[c,d,e] Ivan Gusev,[c] Larissa Gomes Franca,[f] Georgina H. Burgoyne Morris,[f] Martyna Jankowska,[c] Priscila Pazini Abatti,[g] Karol Erfurt,[c] Rachel C. Evans,[f] and Agata Blacha-Grzechnik[c, d]

[a] SPIN-Lab Centre for Microscopic Research on Matter, University of Silesia in Katowice, 75 Pulku Piechoty 1A, Chorzow 41-500, Poland

[b] Institute of Chemistry, University of Silesia in Katowice, Szkolna 9, 40-006 Katowice, Poland.

[c] Faculty of Chemistry, Silesian University of Technology, Strzody 9, 44-100 Gliwice, Poland

[d] Centre for Organic and Nanohybrid Electronics, Silesian University of Technology, Konarskiego 22b, 44-100 Gliwice, Poland

[e] Department of Physics, Durham University, South Road, Durham, DH1 3LE, U.K.

[f] Department of Materials Science and Metallurgy, University of Cambridge, Cambridge CB3 0FS, U.K.

[g] Department of Chemistry, Federal University of Santa Catarina, 88040-900 Florianópolis, Santa Catarina, Brazil

*Corresponding author:

E-mail: aleksandra.nyga@us.edu.pl, marli.ferreira@polsl.pl

Table of Contents

1. Synthesis and characterization of the compounds 3

1.1. Materials and methods 3

1.2. Synthetic procedures and spectroscopy data 3

2. Calculations 11

3. Electrochemical studies 12

3.1. Materials 12

3.2. Electrochemical analysis 12

4. Spectroscopic characterization of porphyrin derivatives 12

5. Photogeneration of singlet oxygen by porphyrin derivatives 13

5.1. Materials 13

5.2. Singlet oxygen photogeneration analysis 13

6. TTA-UC studies 14

6.1. UV/vis absorption spectroscopy 14

6.2. Steady-state photoluminescence (PL) spectroscopy 14

6.3. Upconversion, phosphorescence and time-resolved emission measurements 15

6.3.1. Upconversion and phosphorescence emission measurements 15

6.3.2. UC quantum yield (Φ_{UC}) 15

6.3.3. Lifetime measurements 16

7. Supplementary singlet oxygen photogeneration data 18

8. Supplementary photophysical data 18

9. Supplementary TTA-UC data 19

10. Copies of NMR charts of intermediates and final compounds 21

11. High resolution mass spectrometry spectra of intermediates and final compounds 25

12. References 31

1. Synthesis and characterization of the compounds

1.1. Materials and methods

All the solvents and chemicals were commercially available and used as received. Benzaldehyde (98+%, Acros Organics), 4-bromobenzaldehyde (99%, Angene), pyrrole (99%, Acros Organics), 10*H*-phenoxazine (98%, Angene), 4-iodopyridine (98%, Angene), 2,3-dichloro-5,6-dicyano-1,4-benzoquinone (DDQ, 98%, Sigma-Aldrich), *p*-chloranil (99%, Sigma-Aldrich), triruthenium dodecacarbonyl (98%, Angene), tri-*tert*-butylphosphonium tetrafluoroborate (97+%, Acros Organics), palladium(II) acetate (99.9%, Acros Organics), iodine (99+%, Acros Organics), potassium carbonate (98%, Acros Organics), trifluoroacetic acid (99+%, Fisher Chemical), propionic acid (>99.5%, Chempur), triethylamine (>99%, Fisher Chemical), anhydrous tetrahydrofuran (99.85%, Acros Organics), anhydrous toluene (99.85%, Acros Organics), anhydrous dichloromethane (99.7+%, Acros Organics, stabilized with amylene), Celite® (Roth), and deuterated chloroform ($CDCl_3$, ≥99.8 atom % D, Sigma-Aldrich, with 0.03% (v/v) TMS and 0.5 wt.% silver foil as stabilizer).

Column chromatography was performed using 60 Å silica-gel (230-400 mesh, Supelco) or using Aluminum oxide (neutral, Brockmann I, 40-300 µm, 60A). Thin-layer chromatography (TLC) was performed using TLC Silica gel 60 F254 plates (Merck KGaA), and the materials were visualized with a UV lamp (245 nm, 365 nm).

^1H and $^{13}C\{^1H\}$ NMR spectra were recorded on a Varian Mercury Plus 400 MHz spectrometer or a Varian 300 MHz spectrometer in deuterated chloroform ($CDCl_3$). Chemical shifts are reported in parts per million (ppm, δ) using tetramethylsilane (TMS) as an internal standard and the NMR data were processed in MestReNova.

Fourier-transform infrared spectroscopy (FT-IR) was recorded on a PerkinElmer Spectrum Two FT-IR spectrometer, using attenuated total reflectance (ATR) mode.

High-resolution mass spectrometry (HRMS) analyses were performed on a Waters Xevo G2 Q-TOF mass spectrometer (Waters Corporation) equipped with an ESI source operating in positive-ion mode. Full-scan MS data were collected from 100 to 5000 Da in positive ion mode with a scan time of 0.1 s. To ensure accurate mass measurements, data were collected in centroid mode and the mass was corrected during acquisition using leucine enkephalin solution as an external reference (Lock-Spray TM), which generated a reference ion at m/z 556.2771 Da ($[M + H]^+$) in positive ESI mode. The accurate mass and composition for the molecular ion adducts were calculated using the MassLynx software (Waters) incorporated with the instrument.

1.2. Synthetic procedures and spectroscopy data

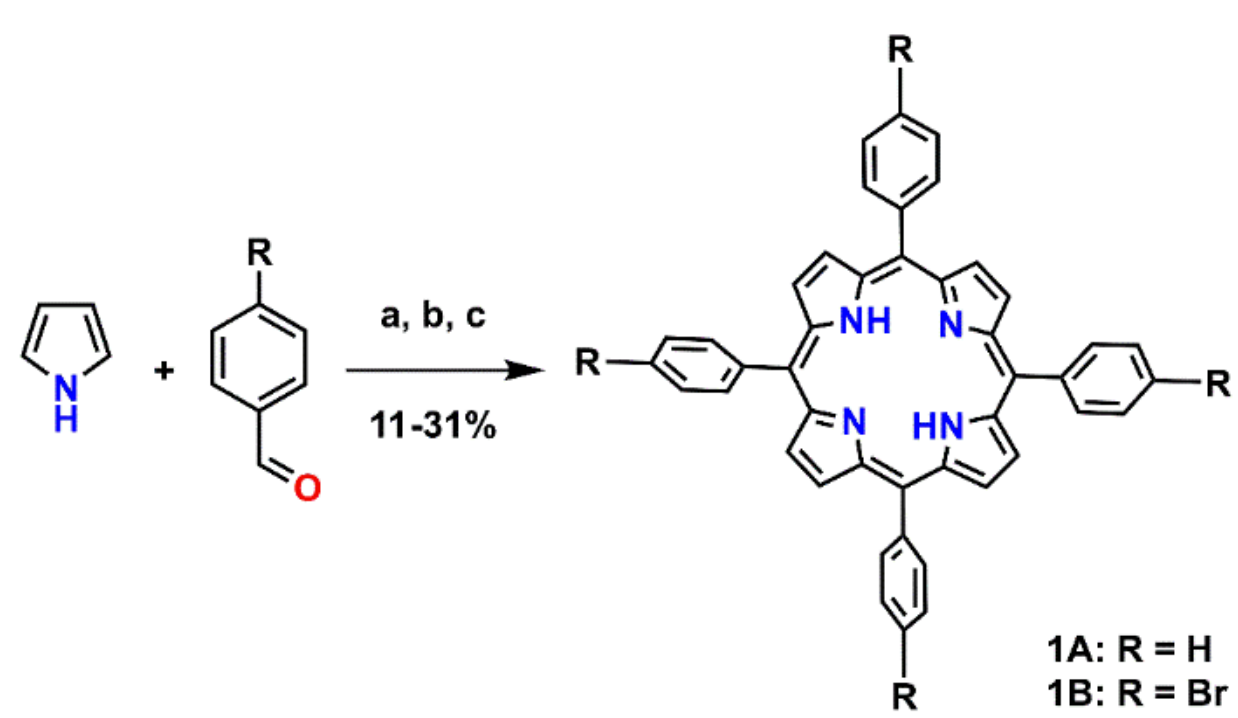

Scheme S1. *Experimental protocols for obtaining free base porphyrins, 5,10,15,20-tetraphenylporphyrin (**1A**) and 5,10,15,20-tetrakis(4'-bromophenyl)porphyrin (**1B**). (a) Lindsey method; (b) Adler-Longo method; (c) Iodine-catalyzed condensation reaction.*

Table S1. *Experimental protocols to obtain free-base porphyrins, 5,10,15,20-tetraphenylporphyrin (**1A**) and 5,10,15,20-tetrakis(4'-bromophenyl)porphyrin (**1B**).*

Method	***Solvent***	***Catalyst***	***Temperature***	***Oxidant***	***Reactant Concentration***	***Time***	***Isolated Yield*** [b]
**Lindsey**	Anhydrous DCM	H^+	25 °C	*p*-chloranil	0.01 M	2 h	**1A** = 31%<br>**1B** = 11%
**Adler-Longo**	Propionic acid	H^+	141 °C	O_2 (air)	0.1 M	3 h	**1A** = 20%<br>**1B** = 14%
**Iodine** [a]	Chloroform	I_2	25 °C	DDQ	0.05 M	10 min	**1A** = 20%<br>**1B** = 17%

Conditions: Pyrrole (1 equiv) and Benzaldehyde (1 equiv). [a] *Undistilled reagents and solvent, 0.1 equiv of* I_2*.* [b] *The formation of **1A/1B** was confirmed by* ^1H *NMR spectroscopy.*

Lindsey method (a):[1] Trifluoroacetic acid (2.05 g, 18.0 mmol, 114.0 g mol^{-1}, 1.8 equiv) was added to a solution of pyrrole (671 mg, 10.0 mmol, 67.1 g mol^{-1}, 1 equiv) and the appropriate benzaldehyde (10.0 mmol, 1 equiv) in dichloromethane (1.0 L), protected from light and under argon. The resulting dark violet solution was stirred at room temperature for 2 hours. After that, *p*-chloranil (2.46 g, 245.9 g mol^{-1}, 10.0 mmol, 1 equiv) was added and the resulting mixture was stirred for 18 hours. After this time, triethylamine was added (7.0 mL), then the solvent volume was reduced, under reduced pressure, and the resulting dark suspension (ca. 100 mL) was filtered over a short pad of neutral alumina.

1A*:* The crude material was purified by flash column chromatography on silica gel in DCM-hexanes (2:1; v/v) mixture. Yielding 475.8 mg (0.774 mmol, 614.8 g mol^{-1}, 31%) of a dark violet solid.

1B*:* The crude material was purified by flash column chromatography on silica gel in DCM-hexanes (1:1; v/v) mixture. Additionally, the product was precipitated from $CHCl_3$-MeOH (1:5; v/v) mixture to yield 255.8 mg (0.275 mmol, 930.3 g mol^{-1}, 11%) of deep purple crystals.

Adler-Longo method (b):[2] Pyrrole (1.68 g, 67.1 g mol^{-1}, 25.0 mmol, 1 equiv) and the appropriate benzaldehyde (25.0 mmol, 1 equiv) were added successively to refluxing propionic acid (250 mL). The resulting dark purple mixture was refluxed under aerobic conditions for 3 hours. After cooling, the crystals were collected by filtration, washed with methanol, and then purified by flash column chromatography on neutral alumina in DCM-hexanes (1:1; v/v) mixture. Additionally, the target product was precipitated from *n*-hexane-$CHCl_3$ (9:1; v/v) mixture.

1A: Yielding 768.5 mg (1.25 mmol, 614.8 g mol^{-1}, 20 %) of deep violet crystals.

1B: Yielding 814.0 mg (0.875 mmol, 930.3 g mol^{-1}, 14%) of deep purple crystals.

Iodine-catalyzed condensation reaction (c):[3] Under argon and light protection, iodine (254 mg, 1.0 mmol, 253.8 g mol^{-1}, 0.1 equiv) was added to a degassed solution of pyrrole (671 mg, 10.0 mmol, 67.1 g mol^{-1}, 1

equiv) and the appropriate benzaldehyde (10.0 mmol, 1 equiv) in chloroform (200 mL) and stirred at room temperature for ca. 10 min (until no starting material remains). The reaction progress was monitored by thin layer chromatography (TLC). After that, 2,3-dichloro-5,6-dicyano-1,4-benzoquinone (2.27 g, 10.0 mmol, 227.0 g mol^{-1}, 1 equiv) was added and stirred for further 30 min. Then the mixture was filtered through a short pad of silica gel and the solvent was evaporated.

1A: The product was purified by column chromatography on silica gel using a DCM–hexanes (2:1; v/v) mixture. A purple band with bright red fluorescence was collected and the solvent was evaporated yielding 307.4 mg (0.50 mmol, 614.8 g mol^{-1}, 20%) of violet crystals.

1B: The product was purified by column chromatography on neutral alumina in hexanes–$CHCl_3$ (3:1; v/v) mixture. A purple band with dark red fluorescence was collected and the solvent was evaporated. Then the target porphyrin was precipitated from *n*-hexane–$CHCl_3$ (5:1; v/v) mixture. Yielding 395.4 mg (0.425 mmol, 930.3 g mol^{-1}, 17%) of deep violet crystals.

5,10,15,20-tetraphenylporphyrin (1A): IR-ATR (*ν*, cm^{-1}): 3314 *ν*(N-H), 3054-3020 *ν*(=C-H), 1594, 1555, 1472, 1441, 1348, 1188, 1178, 1154, 1072, 979, 963 *δ*(CCH), 795, 721 *γ*(=C-H), 696, 560, 504, 420. **^{1}H NMR (400 MHz, CDCl$_3$) *δ* (ppm):** 8.78 (s, 8H, pyrrolic β-H), 8.20 – 8.09 (m, 8H), 7.73 – 7.65 (m, 12H), –2.86 (s, 2H, pyrrolic N-H). **HRMS (ESI-TOF):** *m*/*z* calculated for $C_{44}H_{31}N_4$ [*M* + H]$^+$: 615.2549; found: 615.2551.

5,10,15,20-tetrakis(4'-bromophenyl)porphyrin (1B): IR-ATR (*ν*, cm^{-1}): 3312 *ν*(N-H), 2922 *ν*(=C-H), 1585 *ν*(C=C), 1553, 1484, 1471, 1389, 1347, 1211, 1176, 1069, 1011, 991, 982, 964 *δ*(CCH), 839, 794, 725 γ(=C-H), 500, 456. **^{1}H NMR (400 MHz, CDCl$_3$) *δ* (ppm):** 8.89 (s, 8H, pyrrolic β-H), 8.07 (d, *J* = 8 Hz, 8H), 7.90 (d, *J* = 8 Hz, 8H), –2.89 (s, 2H, pyrrolic N-H). **HRMS (ESI-TOF):** *m*/*z* calculated for $C_{44}H_{27}Br_4N_4$ [*M* + H]$^+$: 930.8928; found: 930.8958.

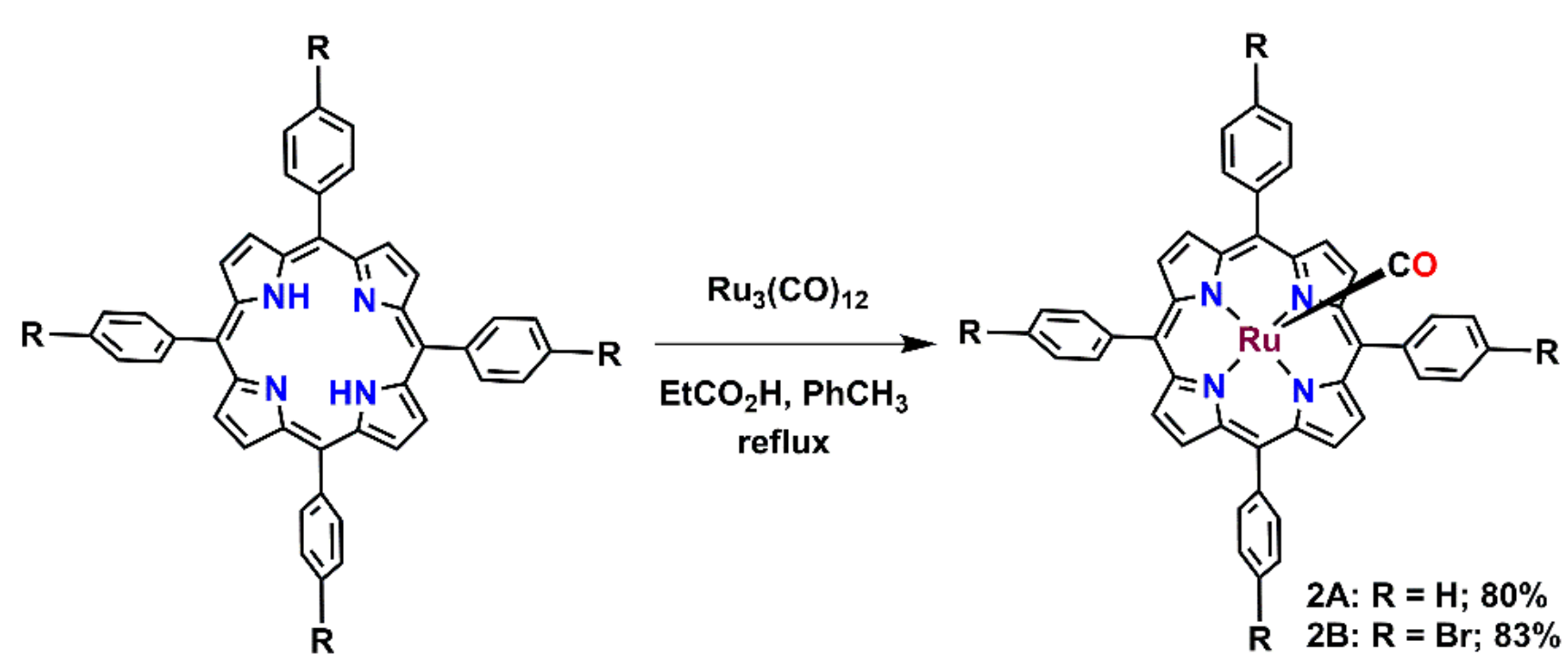

Scheme S2. *Synthetic approach to the insertion of ruthenium into free-base porphyrins.*

Ruthenation of free-base porphyrins: Porphyrins **2A** and **2B** were synthesized according to the literature with minor modifications:[4] The degassed mixture of triruthenium dodecacarbonyl (127.9 mg, 0.20 mmol,

639.3 g mol^{-1}, 2 equiv), the appropriate free-base porphyrin (0.1 mmol, 1 equiv), propionic acid (10 mL), and toluene (10 mL) was refluxed under argon for approx. 6 hours (until no starting material remains). A complete conversion of the free-base porphyrin was observed by TLC (disappearance of the red fluorescence). After this period, the solvent was removed with rotary evaporator and the crude dark red solid was purified by flash column chromatography on silica gel in chloroform. A single red-orange band was collected and the solvent was removed.

5,10,15,20-tetraphenyl-21*H*,23*H*-porphine ruthenium(II) carbonyl (2A): The target porphyrin was precipitated from a *n*-hexane–$CHCl_3$ (2:1; v/v) mixture, yielding 80% of a carmine red solid. **IR-ATR (*ν*, cm^{-1}):** 1945 *ν*(C=O), 1596, 1529, 1486, 1440, 1349, 1306, 1206, 1175, 1068, 1008 *δ*(CCH), 835, 796, 752, 718, 703, 666, 533, 462. **^{1}H NMR (300 MHz, $CDCl_3$) *δ* (ppm):** 8.69 (s, 8H, pyrrolic β-H), 8.27 – 8.09 (m, 8H), 7.82 – 7.66 (m, 12H). **HRMS (ESI-TOF):** *m*/*z* calculated for $C_{45}H_{28}N_4ORu$ $[M]^+$: 742.1307; found: 742.1306.

5,10,15,20-tetrakis(4'-bromophenyl)porphine ruthenium(II) carbonyl (2B): The target porphyrin was precipitated from a *n*-hexane–$CHCl_3$ (5:1; v/v) mixture, yielding 83% of a carmine red solid. **IR-ATR (*ν*, cm^{-1}):** 1967 *ν*(C=O), 1596, 1529, 1477, 1389, 1348, 1307, 1174, 1073, 1006 *δ*(CCH), 797, 722, 486. **^{1}H NMR (400 MHz, $CDCl_3$) *δ* (ppm):** 8.69 (s, 8H, pyrrolic β-H), 8.06 (dd, *J* = 8 Hz, 2 Hz, 4H), 8.21 (dd, *J* = 8 Hz, 2 Hz, 4H), 7.89 (dd, *J* = 8 Hz, 2 Hz, 4H), 7.88 (dd, *J* = 8 Hz, *J* = 2 Hz, 4H). **HRMS (ESI-TOF):** *m*/*z* calculated for $C_{45}H_{24}Br_4N_4ORu$ $[M]^+$ 1057.7686; found: 1057.7900. Additionally, the loss of the CO axial co-ligand was observed, giving $[M - CO]^+$. **HRMS (ESI-TOF):** *m*/*z* calculated for $C_{44}H_{24}Br_4N_4Ru$ $[M - CO]^+$: 1029.7737; found: 1029.7917.

Phenyl ring rotation in ruthenium complexes of TPP derivatives

The phenyl rings are at an angle to the porphyrin plane in ruthenium complexes of tetraphenylporphyrin. The existence of different ligands on the two sides of the Ru porphyrin plane causes nonequivalence of the *ortho* and *meta* protons on the phenyl ring indicating that phenyl ring rotation is slow on the NMR time scale. Consequently, an ABCD spin system[5,6] is observed for the phenyl protons of *para*-bromo tetraphenylporphyrins (see Figure S9 and S12).

Axial functionalization:

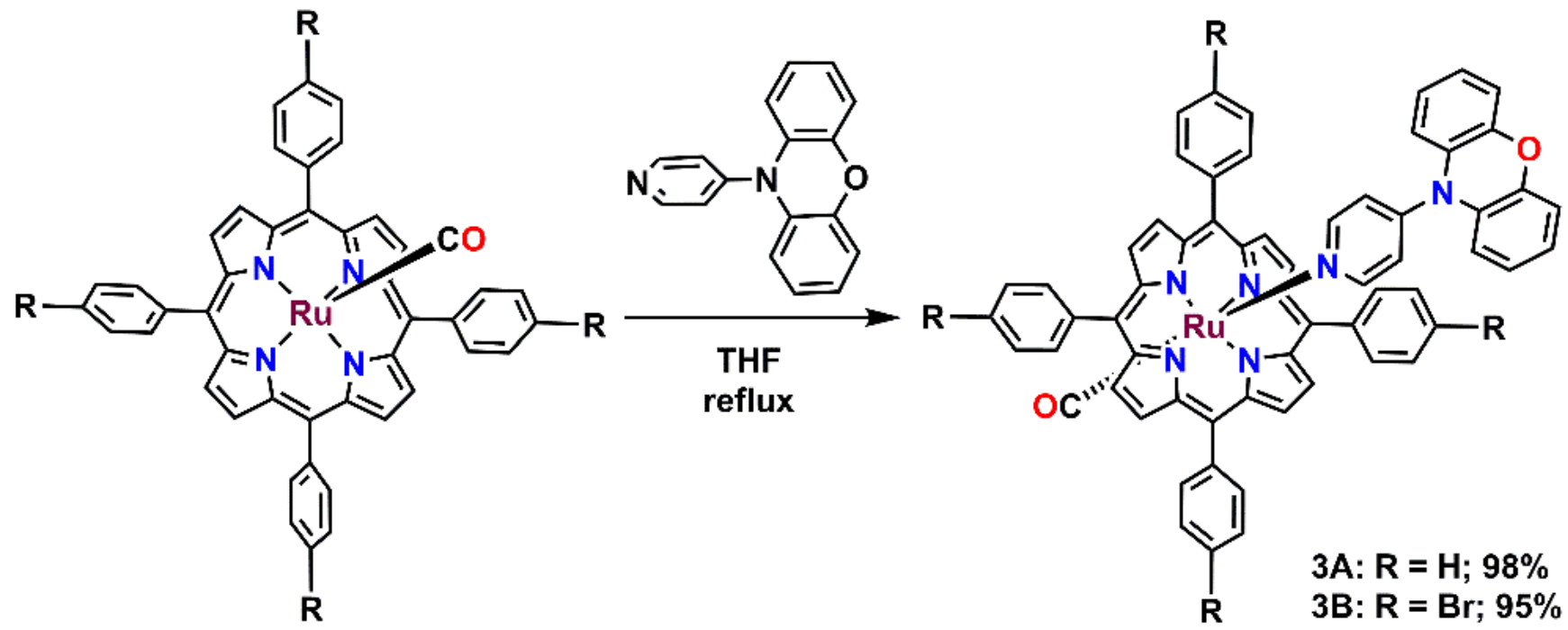

Scheme S3. *Synthetic approach to* ***PyPX*** *ligand insertion into axial position of ruthenium porphyrin.*

The solution of 10-(pyridin-4-yl)-10*H*-phenoxazine (26.0 mg, 0.1 mmol, 260.3 g mol^{-1}, 1 equiv) and the appropriate porphyrin (0.1 mmol, 1 equiv) in anhydrous tetrahydrofuran (20 mL) was refluxed under argon for 20 hours. The solvent was then removed and the dark red solid was purified by column chromatography on silica gel using a hexanes–DCM (1:1; v/v) mixture. A single red band was collected and the solvent was removed.

5,10,15,20-tetraphenyl-21H,23H-porphine ruthenium(II) (carbonyl)-[10-(pyridin-4-yl)-10H-phenoxazine] (3A): Additionally, the target porphyrin was precipitated from a *n*-hexane–$CHCl_3$ (9:1; v/v) mixture. Yield: 98% of a crimson red solid. **IR-ATR (*ν*, cm^{-1}):** 1938 *ν*(C=O), 1624, 1598, 1505, 1479, 1462, 1440, 1347, 1330, 1269, 1228, 1218, 1177, 1071, 1008 *δ*(CCH), 808, 795, 754, 702, 666, 622, 517, 463. **^{1}H NMR (400 MHz, $CDCl_3$) *δ* (ppm):** 8.59 (s, 8H, pyrrolic β-H), 8.27 – 8.17 (m, 4H), 8.06 (d, *J* = 7 Hz, 4H), 7.85 – 7.61 (m, 12H), 6.83 (td, *J* = 8, 1.2 Hz, 2H, PX-H), 6.72 (dd, *J* = 8 Hz, 0.8 Hz, 2H, PX-H), 6.66 (td, *J* = 8 Hz, 1.2 Hz, 2H, PX-H), 6.05 (dd, *J* = 8 Hz, 0.8 Hz, 2H, PX-H), 5.03 (d, *J* = 7 Hz, 2H, Py-H), 1.34 (d, *J* = 7 Hz, 2H, Py-H). **$^{13}C\{^{1}H\}$ NMR (100 MHz, $CDCl_3$) *δ* (ppm):** 151.2, 149.5, 144.2, 143.8, 143.4, 141.3, 135.7, 135.3, 131.8, 129.8, 129.5, 126.2, 125.0, 123.2, 122.9, 122.0, 120.4, 117.4, 108.6. **HRMS (ESI-TOF):** *m*/*z* calculated for $C_{62}H_{40}N_6O_2Ru$ [*M*]$^+$: 1002.2256; found: 1002.2344. Additionally, was observed the loss of CO axial co-ligand, giving [*M* − CO]$^+$. **HRMS (ESI-TOF):** *m*/*z* calculated for $C_{61}H_{40}N_6ORu$ [*M* − CO]$^+$: 974.2307; found: 974.2430.

5,10,15,20-tetrakis(4'-bromophenyl)porphine ruthenium(II) (carbonyl)-[10-(pyridin-4-yl)-10H-phenoxazine] (3B): Further, the target compound was precipitated from a *n*-hexane–DCM (9:1; v/v) mixture. Yield: 95% of a crimson red solid. **IR-ATR (*ν*, cm^{-1}):** 1954 *ν*(C=O), 1601, 1503, 1479, 1463, 1347, 1329, 1267, 1229, 1221, 1209, 1178, 1071, 1005 *δ*(CCH), 803, 792, 758, 715, 620, 486. **^{1}H NMR (400 MHz, $CDCl_3$) *δ* (ppm):** 8.59 (s, 8H, pyrrolic β-H), 8.05 (dd, *J* = 8 Hz, 2 Hz, 4H), 7.95 (dd, *J* = 8 Hz, 2 Hz, 4H), 7.87 (dd, *J* = 8 Hz, 2 Hz, 4H), 7.83 (dd, *J* = 8 Hz, 2.0 Hz, 4H), 6.89 (td, *J* = 8 Hz, 1.2 Hz, 2H, PX-H), 6.78 (dd, *J* = 8 Hz, 1.2 Hz, 2H, PX-H), 6.73 (td, *J* = 8 Hz, 1.6 Hz, 2H, PX-H), 6.20 (dd, *J* = 8 Hz, 1.6 Hz, 2H, PX-H), 5.05 – 4.99

(AA'XX', 2H, Py-H), 1.27 – 1.21 (AA'XX', 2H, Py-H). **$^{13}C\{^{1}H\}$ NMR (100 MHz, $CDCl_3$) δ (ppm):** 151.2, 149.5, 144.2, 143.4, 141.3, 135.7, 135.3, 131.8, 129.8, 129.5, 126.2, 125.0, 123.2, 122.9, 122.0, 120.4, 117.4, 108.6. **HRMS (ESI-TOF):** *m/z* calculated for $C_{61}H_{36}Br_4N_6ORu$ [M − CO]$^+$: 1289.8687; found: 1289.9315. The molecular [M]$^+$ or quasimolecular [M + H]$^+$ ions were not detected. Nevertheless, besides a fragment corresponding to the loss of the axial CO co-ligand, fragmentation involving loss of the axial 10-(pyridin-4-yl)-10*H*-phenoxazine co-ligand was also detected, giving [M − $C_{17}H_{12}N_2O$]$^+$. **HRMS (ESI-TOF):** *m/z* calculated for $C_{45}H_{24}Br_4N_4ORu$ [M − $C_{17}H_{12}N_2O$]$^+$: 1057.7686; found: 1057.7860.

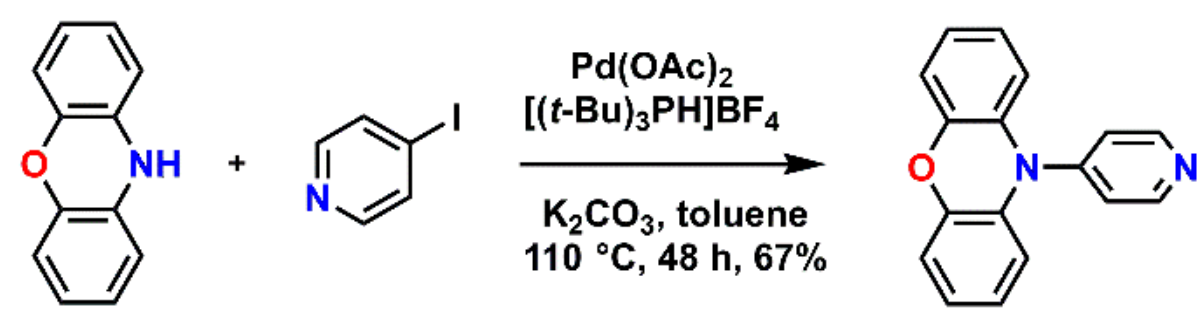

Scheme S4. *Synthesis of 10-(pyridin-4-yl)-10H-phenoxazine ligand (**PyPX**).*

10-(pyridin-4-yl)-10*H*-phenoxazine (PyPX): 10*H*-phenoxazine (2.00 g, 183.2 g mol^{-1}, 10.9 mmol, 1 equiv), 4-iodopyridine (2.46 g, 205.0 g mol^{-1}, 12.0 mmol, 1.1 equiv), potassium carbonate (4.52 g, 138.2 g mol^{-1}, 32.7 mmol, 3 equiv) and anhydrous toluene (24 mL) were added to a Schlenk tube and degassed. Then, palladium(II) acetate (49 mg, 224.5 g mol^{-1}, 0.218 mmol, 2 mol%) and tri-*tert*-butylphosphonium tetrafluoroborate (126.5 mg, 290.1 g mol^{-1}, 0.436 mmol, 4 mol%) were added under a stream of argon gas at room temperature. The reaction mixture was stirred and heated to 110 °C under argon for 48 hours. After that, the mixture was filtered through a Celite® pad, and the solvent was evaporated under reduced pressure. The crude product was purified by flash column chromatography on silica gel using a hexanes–EA (9:1; v/v) mixture. Yield: 1.90 g (260.3 g mol^{-1}, 7.30 mmol, 67%) of a white solid. **IR-ATR (*v*, cm^{-1}):** 1582 *v*(C=C), 1480 *v*(C=N), 1461, 1410, 1323, 1292, 1269, 1203, 1118, 1042, 928, 866, 813, 744 *v*(C-H), 653, 618, 589, 514, 424. **^{1}H NMR (400 MHz, $CDCl_3$) δ (ppm):** 8.64 (br s, 2H), 7.27 (d, J = 6 Hz, 2H), 6.87 – 6.80 (m, 4H), 6.79 – 6.71 (m, 2H), 6.35 (d, J = 8 Hz, 2H). **HRMS (ESI-TOF):** *m/z* calculated for $C_{17}H_{13}N_2O$ [M + H]$^+$: 261.1028; found: 261.1032.

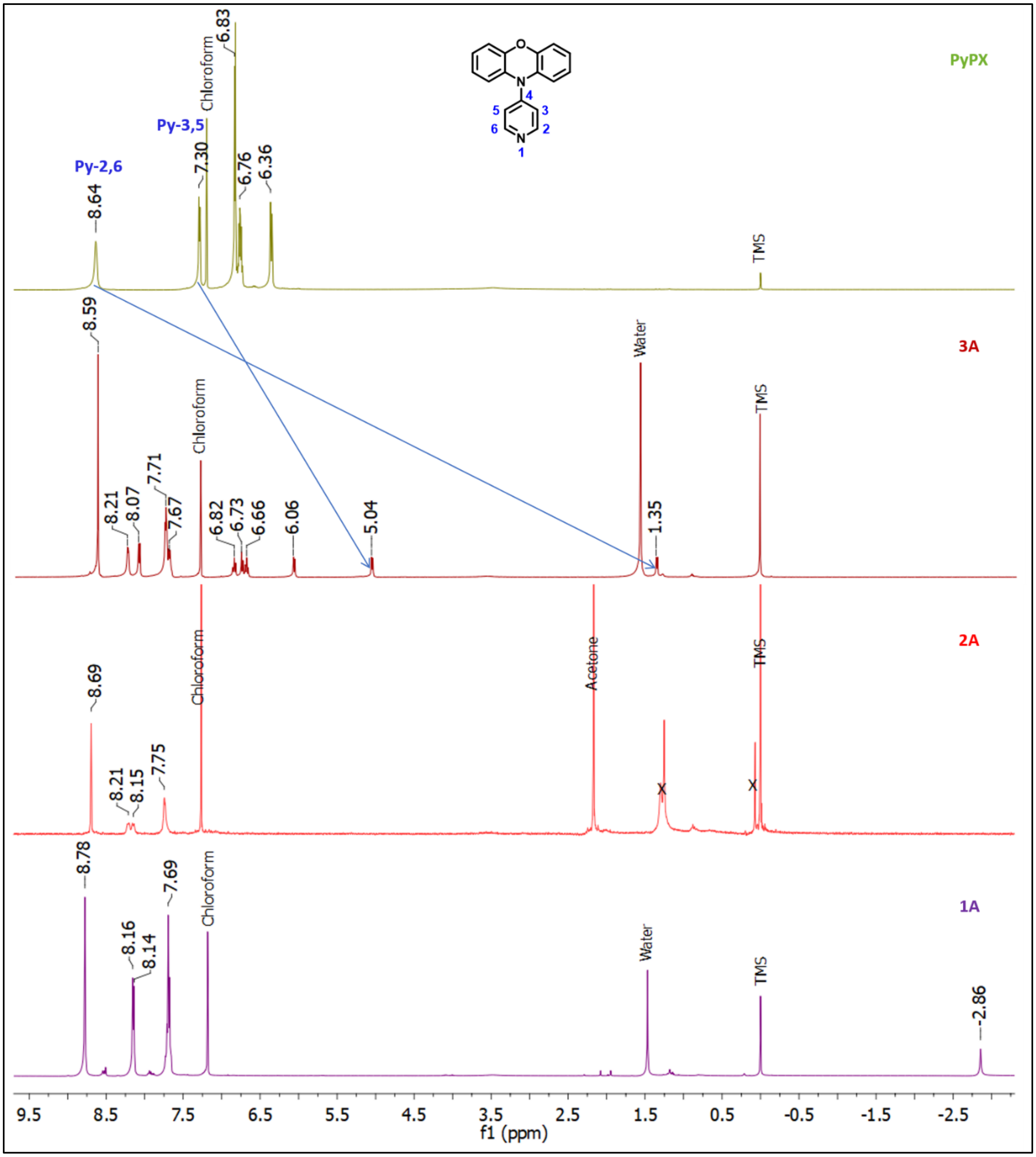

Figure S1. *Comparative* ^{1}*H NMR spectra of* ***PyPX*** *ligand and porphyrins* ***3A****,* ***2A****, and* ***1A*** *in* $CDCl_3$*; X = solvent impurities. The resonances that can be assigned to the pyridyl protons are indicated.*

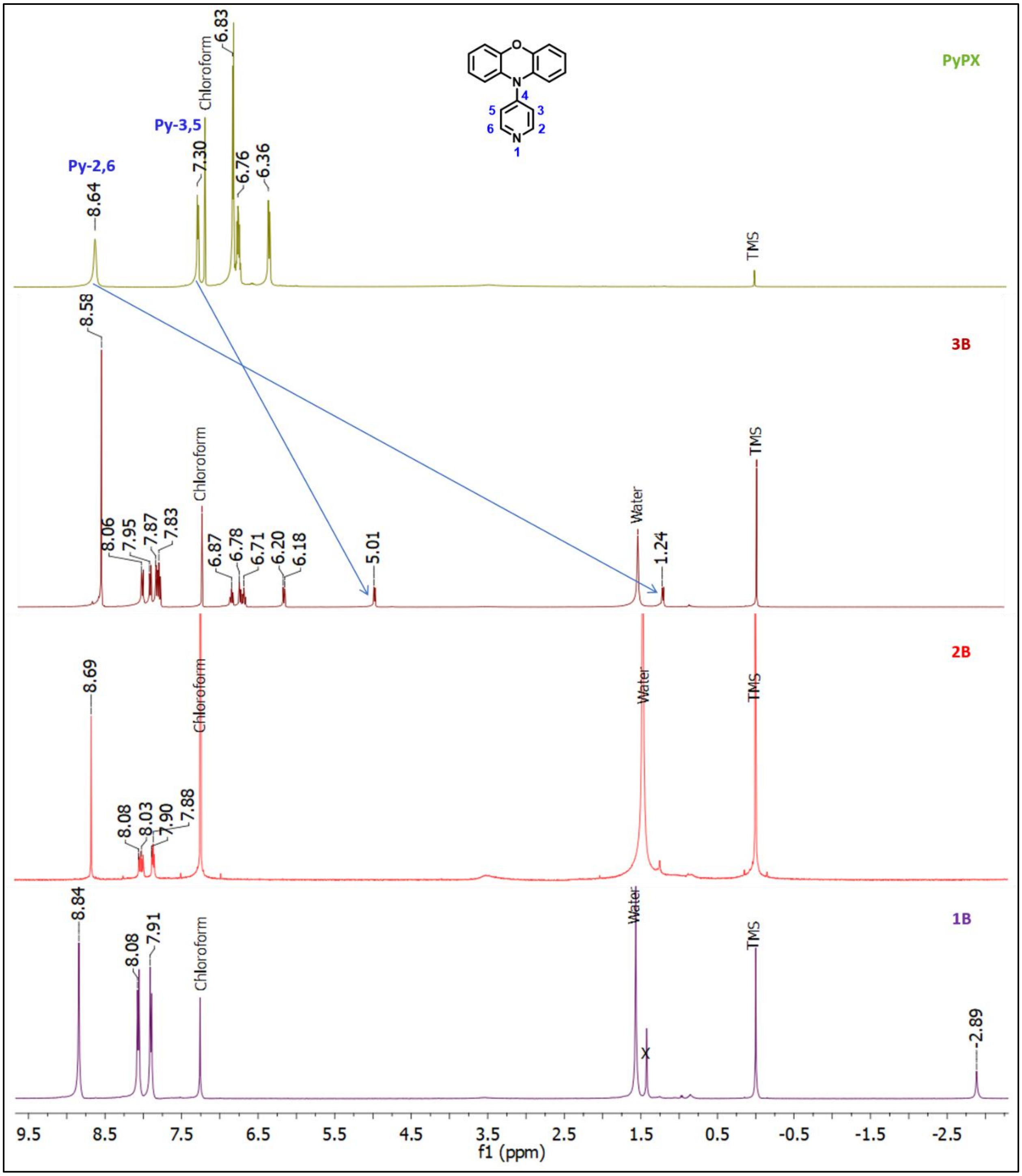

Figure S2. *Comparative* ^{1}H *NMR spectra of* ***PyPX*** *ligand and porphyrins* ***3B****,* ***2B****, and* ***1B*** *in* $CDCl_3$*; X = solvent impurities. The resonances that can be assigned to the pyridyl protons are indicated.*

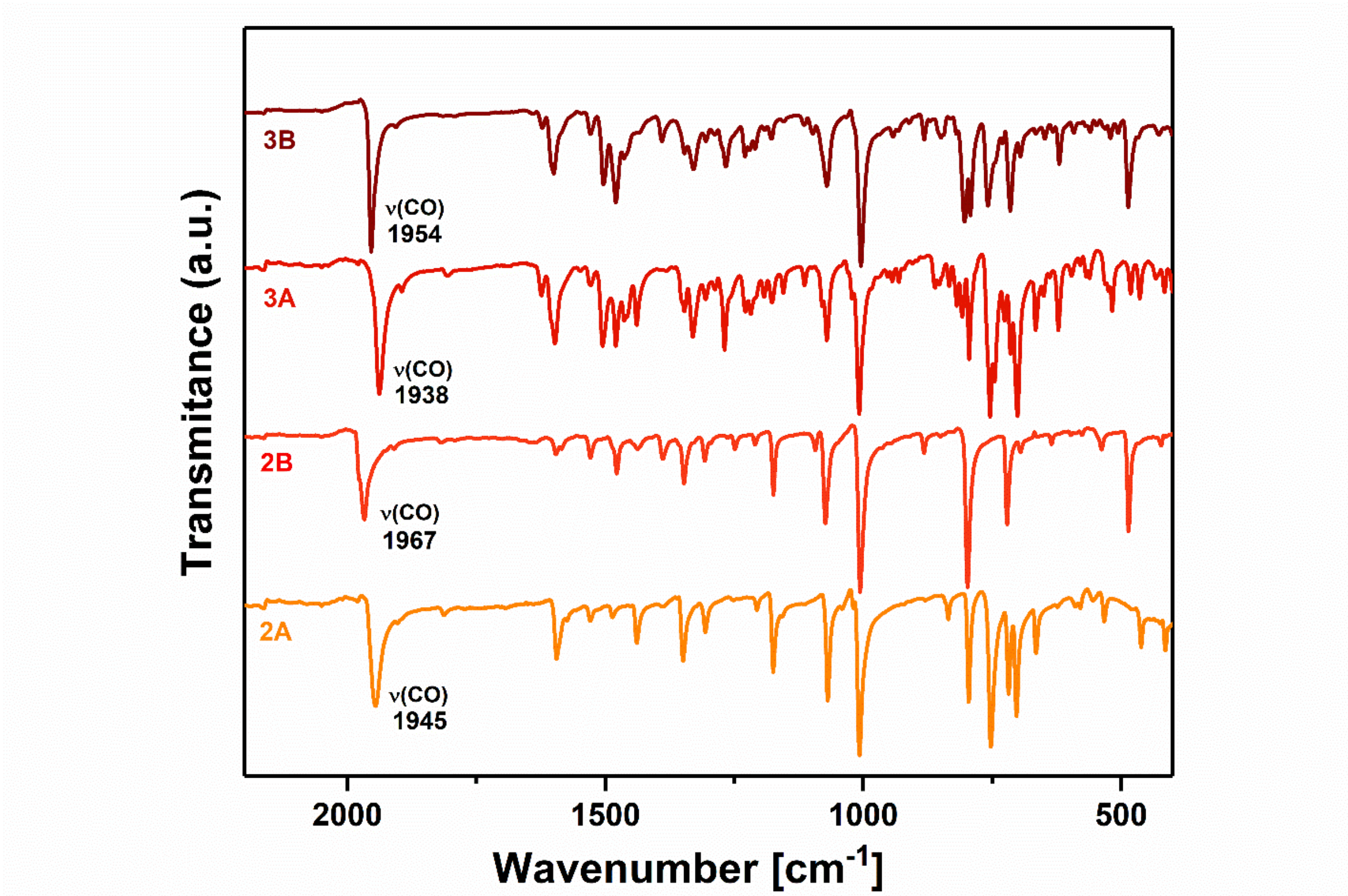

Figure S3. *ATR-FTIR spectra of the ruthenium porphyrin complexes **2A, 2B, 3A** and **3B** showing infrared absorption between 2200 and 400 cm^{-1}.*

2. Calculations

Excited state energy of TD-DFT states was calculated using the resultant T_1 geometry at the B3LYP/def2-TZVP[7] level of theory using CPCM for the appropriate solvent. In this case, relativistically corrected triple-zeta basis sets with the zeroth-order regular approximation (ZORA)[8,9] were used: ZORA-def2-TZVP[7] with the SARC/J[10] auxiliary basis for all atoms except Ru, for which a segmented all-electron relativistically contracted (SARC) SARC-ZORA-TZVP[10] basis set was used. Spin-orbit coupling (SOC) calculations were performed as implemented in the ORCA software. SOC matrix elements (SOCME) and SOC-corrected excitations (SOC states) were computed using the same settings. The RI-SOMF(1X) setting was used to accelerate SOC calculations. These settings have previously been used to model an earlier example of a TADF di-Ir(III) complex[11] and has shown perfect agreement between the computational model and the experiment. All molecular orbital (MO) iso surfaces were visualized using Gabedit 2.5.0.[12]

We use density functional theory (DFT) and time-dependent DFT (TD-DFT) as well as the quasi-degenerate perturbation theory (QDPT)[13,14] with zeroth-order regular approximation (ZORA)[8,9] implemented in Orca

5.0.3 [15,16] in order to gain an additional insight into the triplet state properties of porphyrin ligands and their complexes. Ground state (S_0) geometries were optimized at the BP86[17]/def2-TZVP[7]/CPCM(CH_2Cl_2) level of theory. We used the def2/J[18] auxiliary basis set and atom-pairwise dispersion correction with the Becke-Johnson damping scheme (D3BJ)[19,20] as supplementary settings. All optimizations were performed with tight SCF and geometry convergence criteria. All geometries were verified to be true energy minima by a frequency calculation, and no negative frequencies were found. Excited state energy of TD-DFT states was calculated using the resultant S_0 geometry. In this case, relativistically corrected triple-zeta basis sets with the zeroth-order regular approximation (ZORA)[8,9] were used: B3LYP/ZORA-def2-TZVP[7] with the SARC/J [10] auxiliary basis was used for all atoms except for Ru, for which a segmented all-electron relativistically contracted (SARC) SARC-ZORA-TZVP was used. Spin-orbit coupling (SOC) calculations were performed as implemented in the ORCA software. SOC matrix elements (SOCME) and SOC-corrected excitations (SOC states) were computed using the same settings as for the TD-DFT states. The RI-SOMF(1X) setting was used to accelerate SOC calculations. Singlet and triplet radiative rates were calculated according to a previously described methodology. [11,21,22] All molecular orbital (MO) iso surfaces were visualized using Gabedit 2.5.0. [12]

3. Electrochemical studies

3.1. Materials

Electrochemical measurements were performed in dichloromethane (HPLC grade, Sigma-Aldrich) containing tetrabutylammonium tetrafluoroborate ($TBABF_4$, 99%, Sigma Aldrich) as the supporting electrolyte.

3.2. Electrochemical analysis

Measurements were carried out in a 0.1 M $TBABF_4$ solution in DCM, purged with argon prior to analysis, using an SP-150 electrochemical workstation (Bio-Logic) and a three-electrode system consisting of a platinum disk electrode (Pt, EDAQ, 1 mm in diameter) acting as a working electrode, a silver wire as a pseudo-reference electrode and a platinum wire as the counter electrode. CV curves were recorded in the range from −2.3 to 1.0 V versus the ferrocene redox couple (Fc/Fc^+) potential at a scan rate of 0.05 V/s. The concentration of the tested compounds in an electrolyte solution was equal to 0.5 mM.

4. Spectroscopic characterization of porphyrin derivatives

The UV-Vis absorption spectra in CH_2Cl_2 were recorded using the Hewlett Packard 8452A UV-Vis absorbance spectrometer with 10 × 4 mm quartz cuvettes (10 mm path length; Hellma Analytics). For

photodegradation studies, the solution of the photoactive molecule was illuminated with either a white light xenon lamp (SLS205 Light Source, ThorLabs) or a 405 nm laser (Oxxius LBX-05-50-CSB-PPA). Photoluminescence properties of porphyrin ligands and Ru(II) complexes were studied in solvent glass at 77 K and were recorded in dilute ~10^{-5} M 2-methyltetrahydrofuran solutions degassed using two freeze-pump-thaw cycles. Temperature was controlled using a VNF-100 liquid nitrogen cryostat (Janis Research). Photoluminescence spectra were recorded using a QePRO spectrometer (Ocean Optics) and a 405 nm LED excitation source, while photoluminescence decay traces were recorded using a Horiba Delta Flex system equipped with a 330 nm SpectraLED light source.

5. Photogeneration of singlet oxygen by porphyrin derivatives

5.1. Materials

For singlet oxygen ($^1O_2$) photogeneration, a 2,3,4,5-tetraphenylcyclopentadienone (TPCPD, 98%, Aldrich) solution in dichloromethane (HPLC grade, Sigma Aldrich stabilized with methanol) was used as a photochemical trap. The quantum yield of the photogeneration process (Φ_Δ) was determined with use of tetraphenylporphyrin (**1A**) as a reference.

5.2. Singlet oxygen photogeneration analysis

Experiments were carried out in dichloromethane with 0.05 mM TPCPD under either broadband visible light or laser illumination (Figure S4).

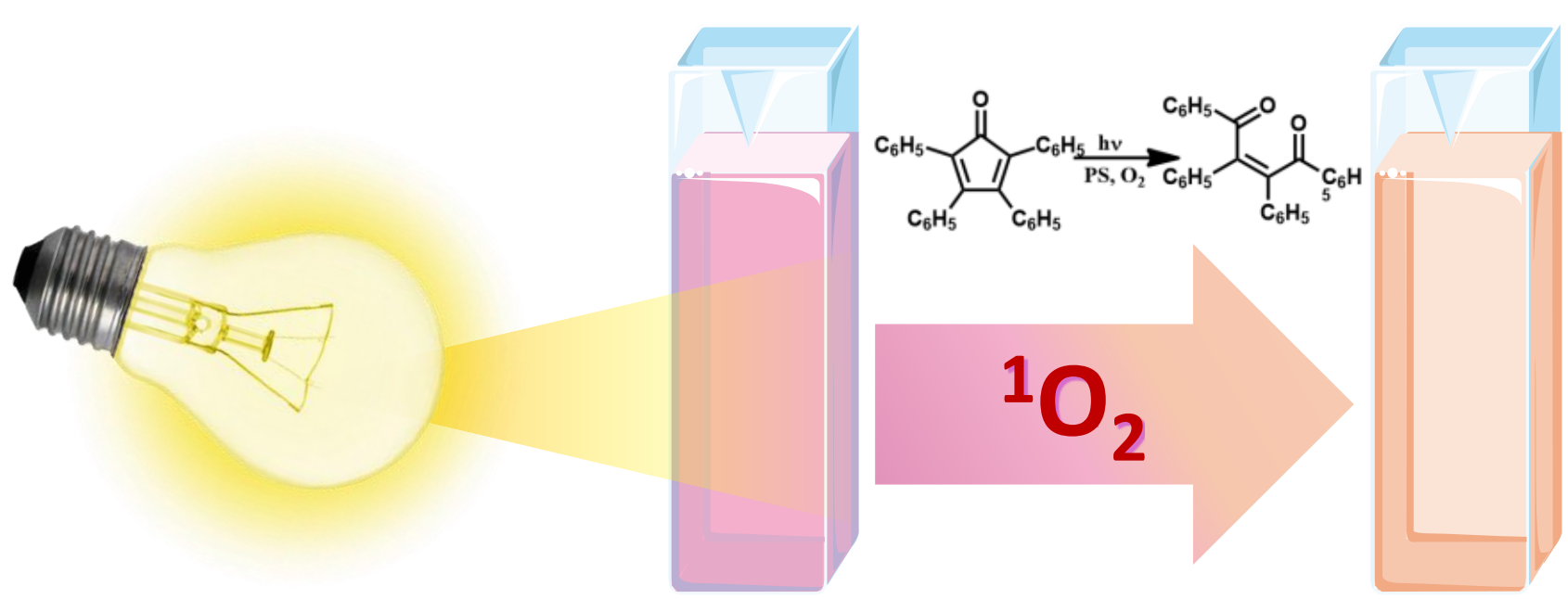

Figure S4. *TPCPD as a trap for $^1O_2$. Under visible/405 nm excitation the Ru(II) porphyrin generates $^1O_2$, which reacts with pink TPCPD converting it into a colorless endoperoxide. The remaining orange color arises from the photosensitizer/oxidation mixture. The decrease in the 512 nm TPCPD absorption band is used to calculate Φ_Δ.*

As in our previous studies,[23–25] and as detailed in Section 4, in situ UV–Vis kinetics were recorded in quartz cuvettes (10 mm path length). The sample was irradiated with xenon lamp or with 405 nm laser, with the excitation beam aligned orthogonally to the spectrometer detection path across the 4 mm dimension. Spectra were collected every 30 s over 5 min while monitoring the TPCPD absorption at 512 nm. A visible

change in the color of the solution, together with a progressive decrease in absorbance, indicated the progress of the photochemical process. Singlet oxygen ($^1O_2$) quantum yields (Φ_Δ) were measured with reference to standard 5,10,15,20-tetraphenylporphyrin (Φ_{TPP} = 55% in $CHCl_3$)[26] and calculated with accordance to Eq. S1:

$$\Phi_\Delta = \Phi_{TPP} \cdot \frac{k_i}{k_{TPP}} \cdot \frac{\alpha_{TPP}}{\alpha_i} \quad \text{(Eq. S1)}$$

where Φ_Δ and Φ_{TPP} are quantum yields of $^1O_2$ photogeneration of the tested photosensitizer and the TPP, respectively; k_i and k_{TPP} are the rate constants of TPCPD oxidation in the presence of the tested photosensitizer and TPP, respectively; α is absorption correction factor $\alpha = 1\text{-}10^{-A}$, where A is absorbance at the irradiation wavelength, in this case 405 nm. During the calculations, a correction was applied by subtracting the spectrum of TPCPD from the total signal to isolate the porphyrin absorbance at 405 nm. Broadband xenon illumination was used to compare only kinetics, as it excites a wide spectral range and bleaching becomes significant during the measurement.

6. TTA-UC studies

6.1. UV/vis absorption spectroscopy

UV/Vis absorption spectra were measured with a PerkinElmer Lambda 750 spectrophotometer. Measurements were carried out on solutions in THF in quartz SUPRASIL® cuvettes (10 mm pathlength). Absorption spectra of emitter solutions were taken using a wavelength scan with a resolution of 1 nm at a scan speed of 266.75 nm/min and a slit width of 2 nm. A reference sample of THF in an identical cuvette was used to apply a 100% transmission correction.

6.2. Steady-state photoluminescence (PL) spectroscopy

Steady-state PL spectroscopy was performed on a Fluorolog-3 spectrophotometer (Horiba Jobin Yvon). Solution emission spectra were recorded in the right-angle configuration using a single scan. The emission slits were adjusted so that the maximum PL intensity was within the range of linear response of the detector. Emission spectra were corrected for the wavelength response of the system and the intensity of the lamp profile over the excitation range, respectively, using correction factors supplied by the manufacturer.

6.3. Upconversion, phosphorescence and time-resolved emission measurements

The UC emission and phosphorescence spectra, UC quantum yield (Φ_{UC}) and lifetime measurements were performed using an FLS1000 time-correlated single photon counting (TCSPC) spectrometer (Edinburgh Instruments Ltd.). Samples were excited with a 532 nm laser (MGL-III-532, 200mW) and detected using a visible PMT-980 detector. All samples were deaerated by bubbling N_2 through the solutions for 30 minutes in a sealed cuvette. While the N_2 flow rate is not known, all samples were processed under identical conditions, ensuring consistent deaeration across the set.

6.3.1. Upconversion and phosphorescence emission measurements

All spectra were measured outside integrating sphere, with the laser power set to the maximum power 100 W cm^{-2}. For all UC emission measurements, spectra were collected from 380 to 500 nm with a step size of 1 nm and a dwell time of 0.1 s, and a short-pass filter (cut-off 500 nm, Thorlabs) placed in front of the detector. For all phosphorescence emission measurements, spectra were collected from 600 to 950 nm with a step size of 1 nm and a dwell time of 0.1 s, and a long-pass filter (cut-off 550 nm, Thorlabs) placed in front of the detector. The sensitizer and annihilator concentrations (10 µM and 400 µM, respectively) were kept constant for all TTA-UC experiments to enable direct comparison of the photosensitizing performance of the investigated complexes. These concentrations were selected following established TTA-UC sample preparation guidelines and previous TTA-UC studies.[27,28]

6.3.2. UC quantum yield (Φ_{UC})

For upconversion quantum yield (Φ_{UC}) measurements, all spectra were measured within an integrating sphere (SNS125 5-inch sphere, three windows, International Light Technologies). A baffle is placed in front of the observation window, which blocks any scattering and reflection of the laser from the sample surface. The laser power was set to maximum power 100 W cm^{-2}. To calculate Φ_{UC}, four data sets were collected for each sample: 1. sample emission (E_s); 2. sample scattering (S_s); 3. blank THF solution emission (E_b); 4. blank THF solution scattering (S_b).

For all UC emission measurements, spectra were measured from 380 to 500 nm with a step size of 1 nm and a dwell time of 0.1 s, and a short-pass filter (cut-off 500 nm, Thorlabs) placed in front of the detector. For all measurements of scattered laser intensity, spectra were measured from 530 to 534 nm with a step size of 0.1 nm and a dwell time of 0.1 s, and a neutral density filter (OD = 3) placed in front of the excitation source. All spectra were taken with three averaged scans and corrected for the transmission of the relevant filters in the relevant wavelength range.

The Φ_{UC} for each sample was calculated using:

$$\Phi_{\mathrm{UC}} = \frac{E_s - E_b}{S_s - S_b} \quad \text{(Eq. S2)}$$

where E is the integrated photon counts from emission spectra, and S is the integrated photon counts from the scattering spectra. The subscripts s and b indicate measurements with the sample present and those using the blank THF solution, respectively.

During the calculation, all data were corrected by the transmittance of each filter used, and normalized based on the slit-width, scan step and the scan duration used. A quantum yield is defined as the ratio of absorbed to emitted photons, meaning the upconversion quantum yield is limited to 50% since this is a bimolecular process. While some papers report this as a normalized value, Φ_{UC} is reported to its un-normalized value here, to a maximum of 50%.[29]

6.3.3. Lifetime measurements

Phosphorescence (Ph) decay profiles were measured at an emission wavelength of 720 nm using a long-pass filter (cut-off 550 nm) placed in front of the detector. For upconversion (UC) decays, emission decay was collected at 440 nm for DPA as sensitizer and 470 nm for perylene as sensitizer, with a short-pass filter (cut-off 500 nm) placed in front of the detector. The pulse repetition rate, collection time window, and pulse width were adjusted to best capture the decay profile, but kept consistent for equivalent measurements on different samples. The instrument response function (IRF) was measured using a SiO_2 particle suspension solution (Ludox® colloidal silica) using a neutral density filter (OD = 3) in front of the excitation source, and with no emission filter. IRF scans were measured immediately after the corresponding decays using the same instrument parameters.

Individual tail-fits were applied to each emission decay curve using the FAST software package (Edinburgh Instruments) using a multiexponential decay function:

$$I(t) = \sum_{i}^{n} \alpha_i e^{-t/\tau_{\mathrm{i}}} \quad \text{(Eq. S3)}$$

where α_i and τ_{i} are the amplitude and lifetime of the i_{th} component, respectively, and α_i is normalized to unity for n components. The goodness of fit was assessed using the reduced chi-square statistics (χ^2) and the randomness of the residuals. The fractional contributions (f_i) are also reported, where

$$f_i = \frac{\alpha_i \tau_i}{\sum_j^n \alpha_j \tau_j}$$ (Eq. S4)

The average lifetime (<τ>) was then determined using:

$$\langle \tau \rangle = \sum_i^n f_i \tau_i$$ (Eq. S5)

7. Supplementary singlet oxygen photogeneration data

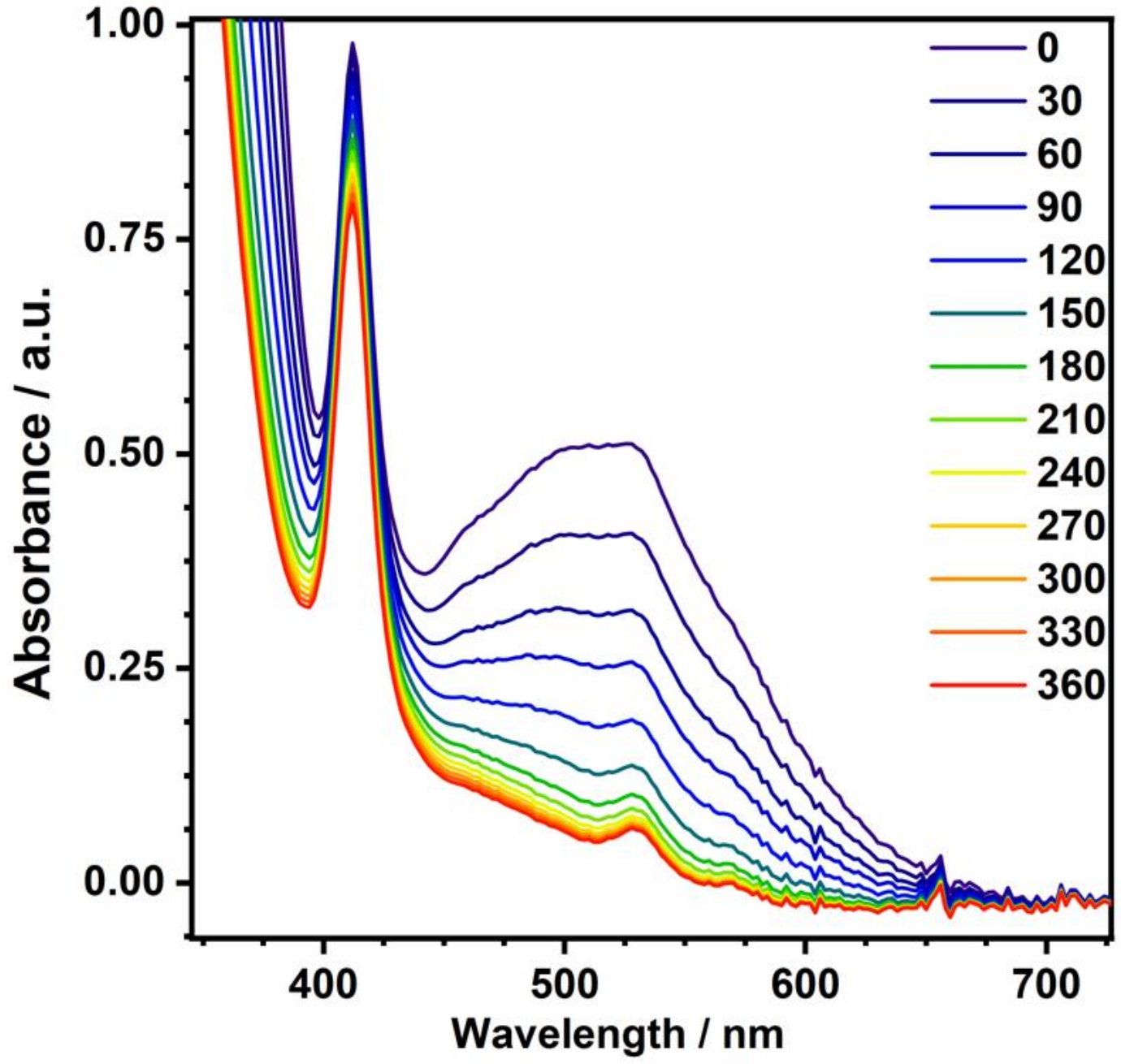

Figure S5. *Set of recorded UV-Vis absorption spectra showing changes in absorbance over time for the photooxidation reaction of TPCPD with singlet oxygen produced by the studied* ***3A*** *porphyrin derivative. In this case the porphyrin was irradiated with 405 nm laser, measurements were performed in DCM solution.*

8. Supplementary photophysical data

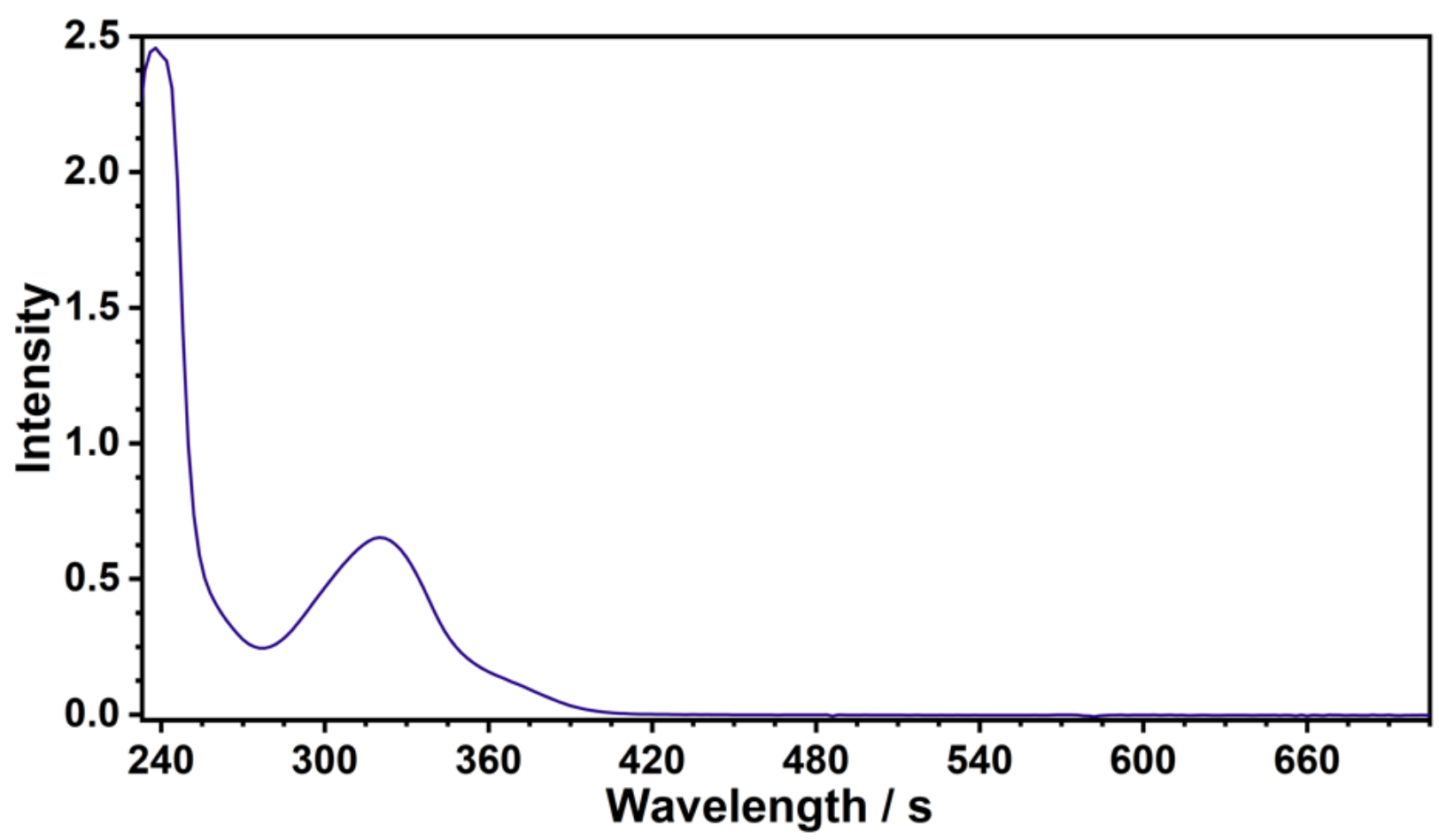

Figure S6. *UV-Vis absorption spectrum of 10-(pyridin-4-yl)-10H-phenoxazine (****PyPX****) ligand in DCM solution.*

9. Supplementary TTA-UC data

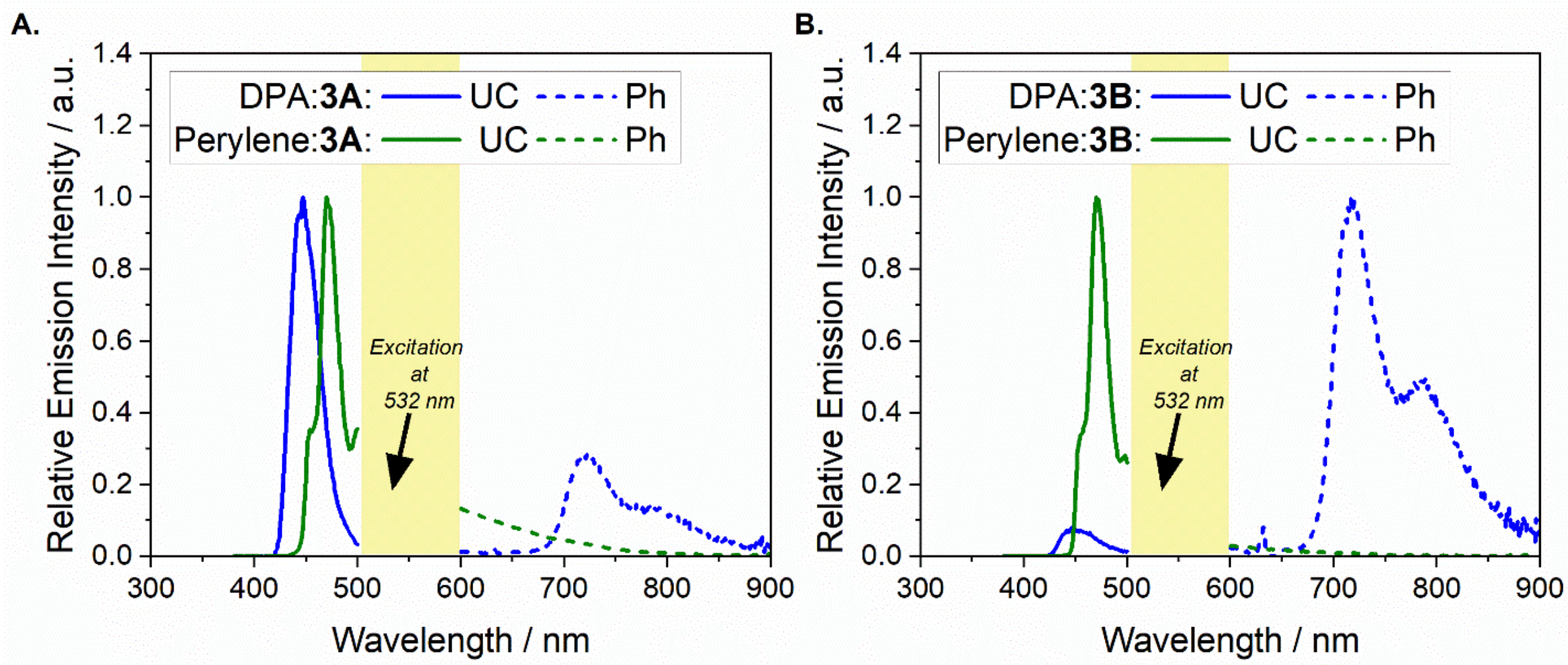

Figure S7: *Upconverted (380-500 nm, solid lines) and phosphorescence relative emission spectra (600-950 nm, dashed lines) of the TTA-UC pairs in THF solutions (400:10 µM), using DPA and perylene as annihilators, and A.:* ***3A*** *and B.:**3B**, as sensitizers.* λ_{ex}*= 532 nm.*

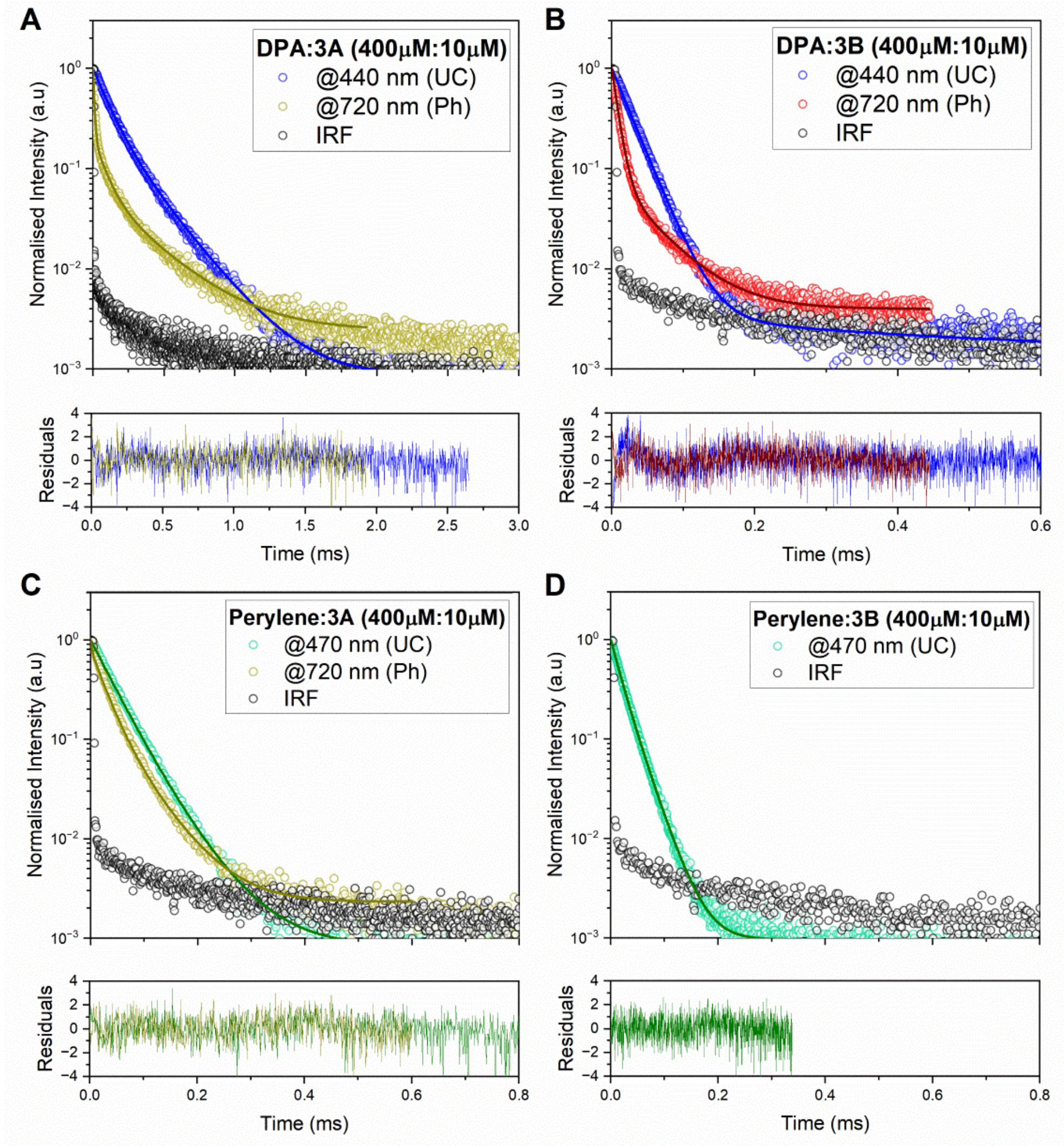

Figure S8: *Kinetic studies of TTA-UC systems in THF solutions at an emitter:sensitizer concentration ratio of 400:10 µM. A: DPA:**3A**, B:DPA:**3B**, C: Perylene:**3A**, D:Perylene:**3B**. Upconversion (UC) decay traces were collected at 440 nm for TTA-UC systems using DPA as the emitter and at 470 nm for systems using perylene as the emitter. Phosphorescence (Ph) decay traces were collected at 720 nm for all systems using both sensitizers (**3A** and **3B**). Experimental data are shown as open symbols and exponential decay fits as solid lines, and the corresponding residuals are included. All samples were degassed by N_2 purging for 30 minutes prior to measurement, and the experiments were performed at room temperature. λ_{ex}= 532 nm.*

10. Copies of NMR charts of intermediates and final compounds

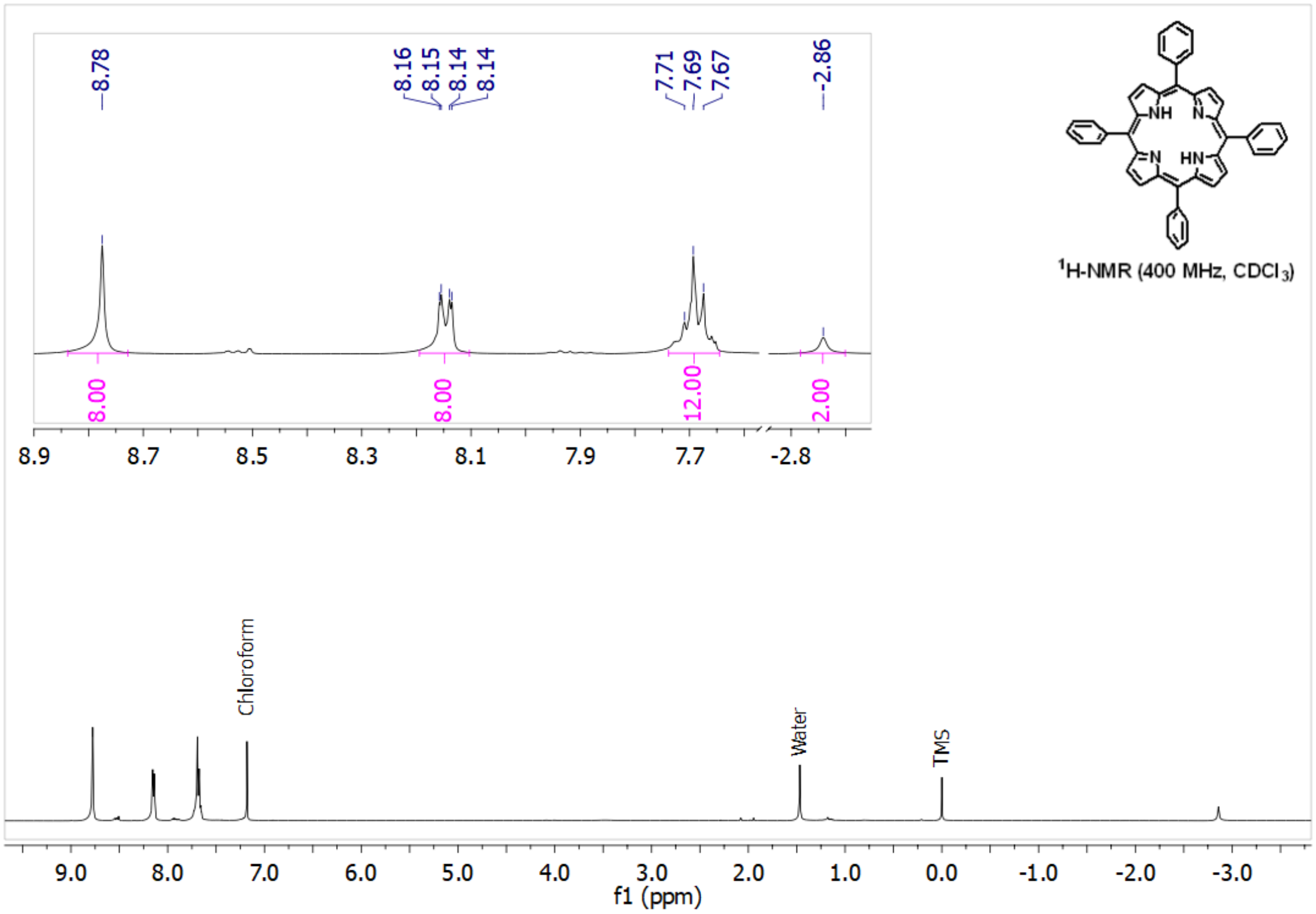

Figure S9. *¹H NMR spectrum of 5,10,15,20-tetraphenylporphyrin (**1A**).*

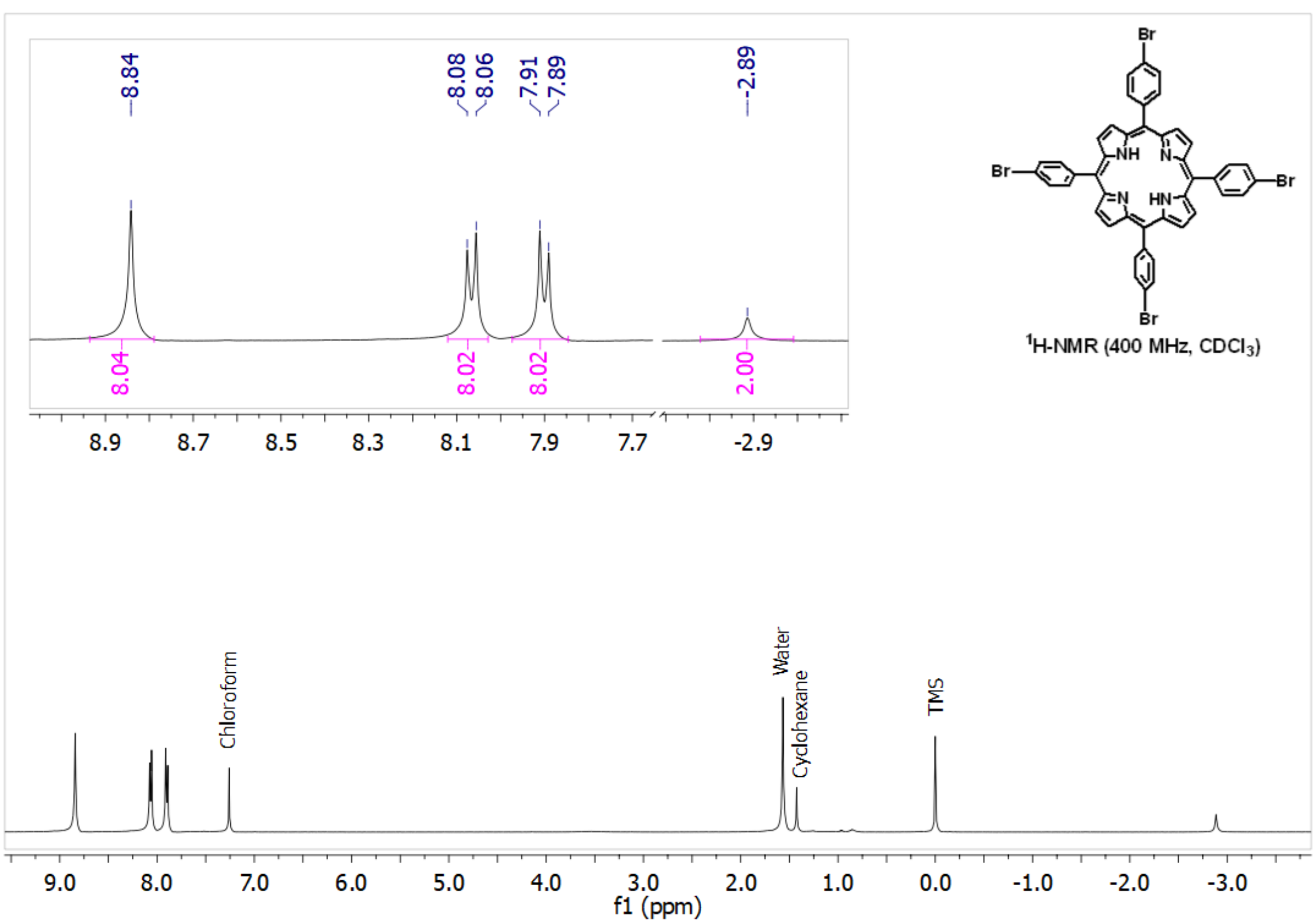

Figure S10. *¹H NMR spectrum of 5,10,15,20-tetrakis(4'-bromophenyl)porphyrin (**1B**).*

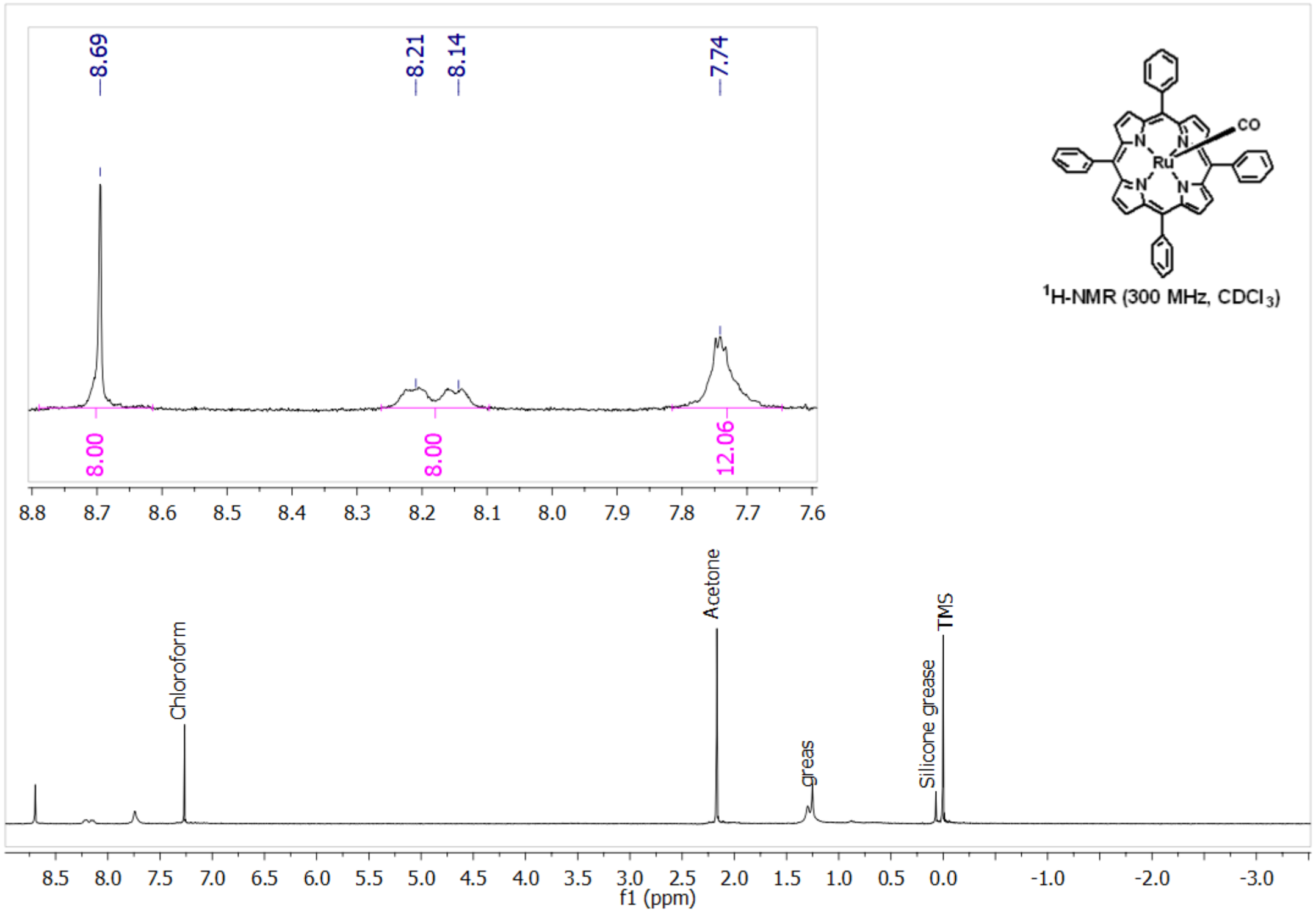

Figure S11. *^{1}H NMR spectrum of 5,10,15,20-tetraphenyl-21H,23H-porphine ruthenium(II) carbonyl (**2A**).*

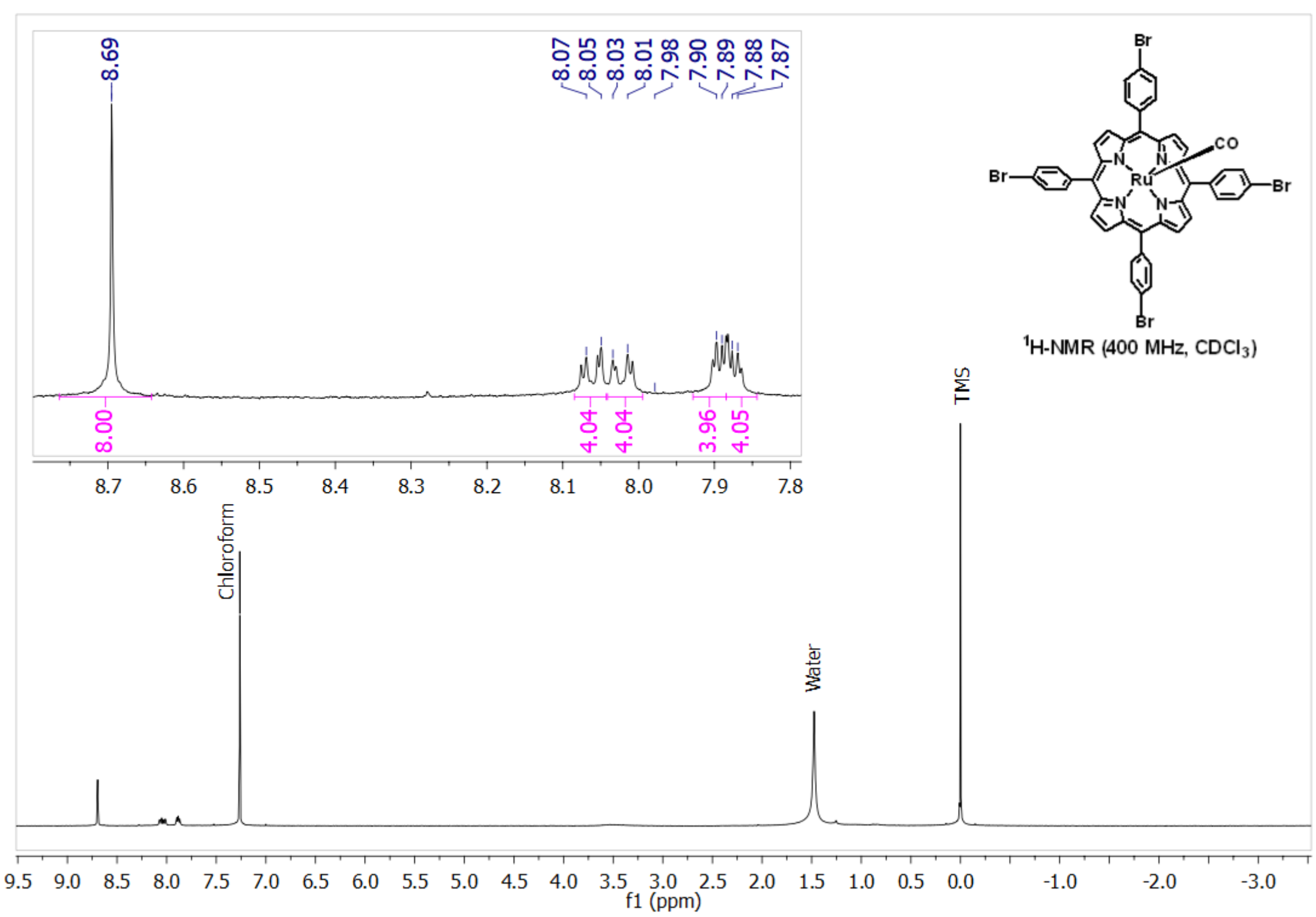

Figure S12. *^{1}H NMR spectrum of 5,10,15,20-tetrakis(4'-bromophenyl)porphine ruthenium(II) carbonyl (**2B**).*

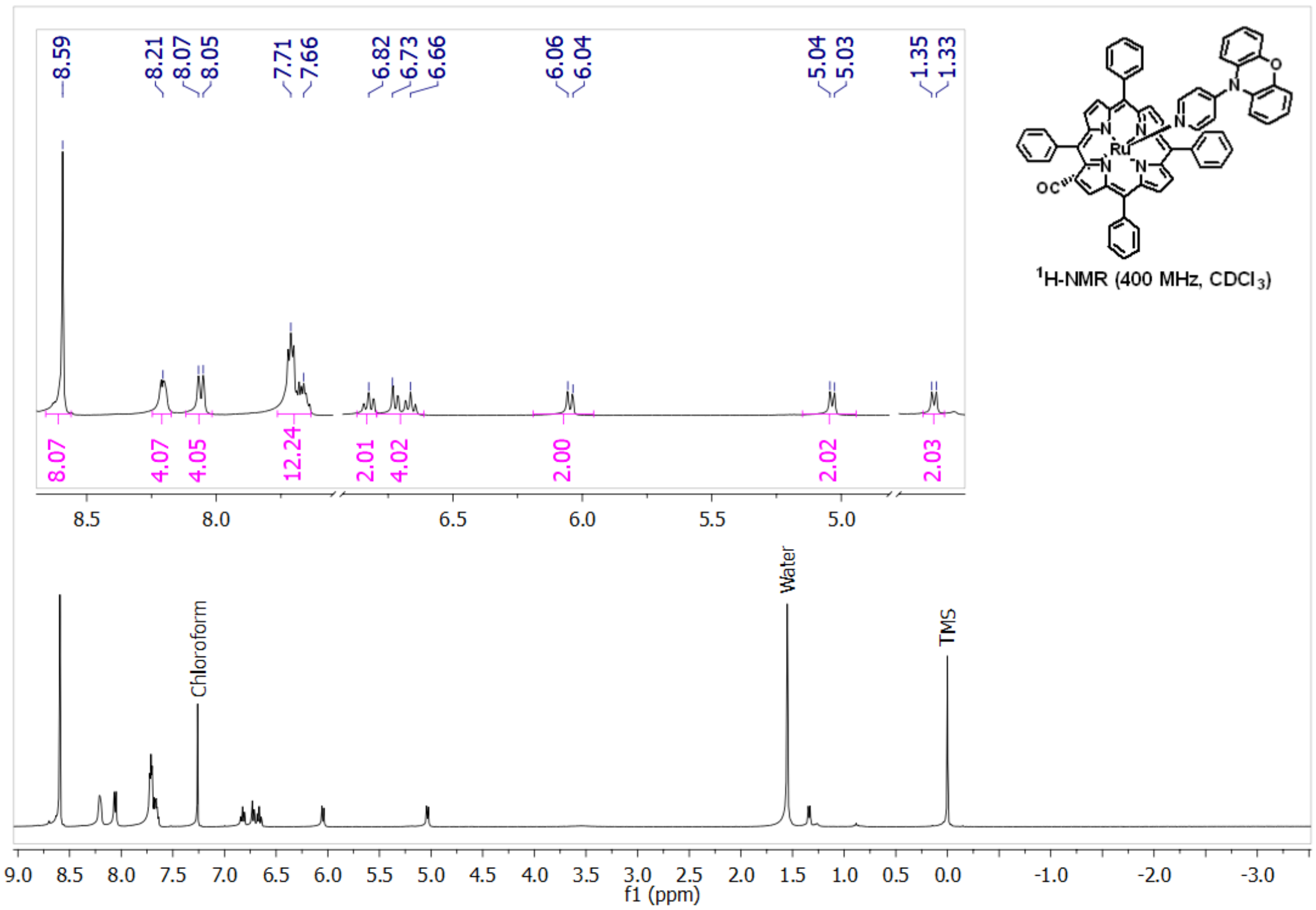

Figure S13. *^{1}H NMR spectrum of 5,10,15,20-tetraphenyl-21H,23H-porphine ruthenium(II) (carbonyl)-[10-(pyridin-4-yl)-10H-phenoxazine] (**3A**).*

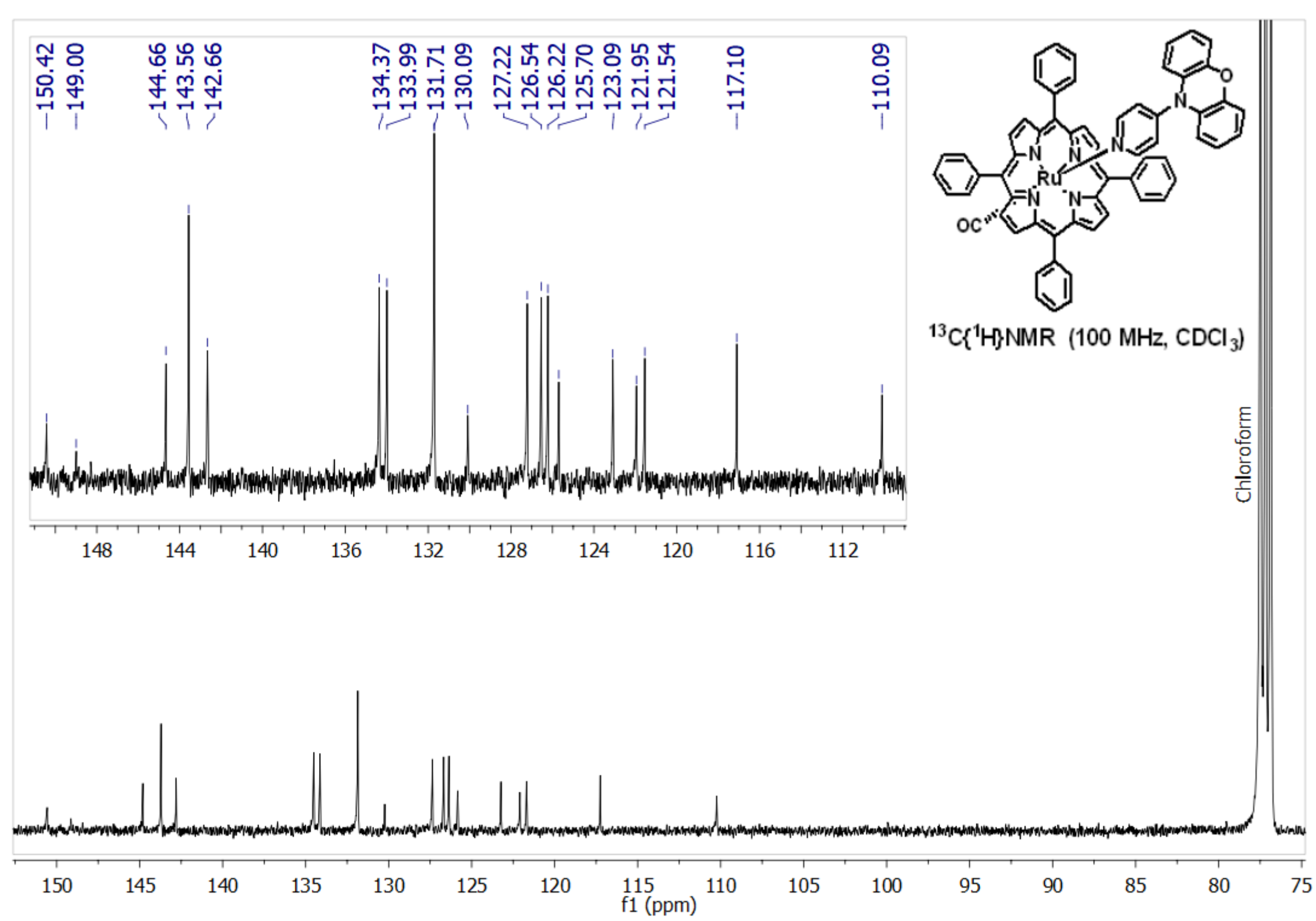

Figure S14. *$^{13}C\{^1H\}$NMR spectrum of 5,10,15,20-tetraphenyl-21H,23H-porphine ruthenium(II) (carbonyl)-[10-(pyridin-4-yl)-10H-phenoxazine] (**3A**).*

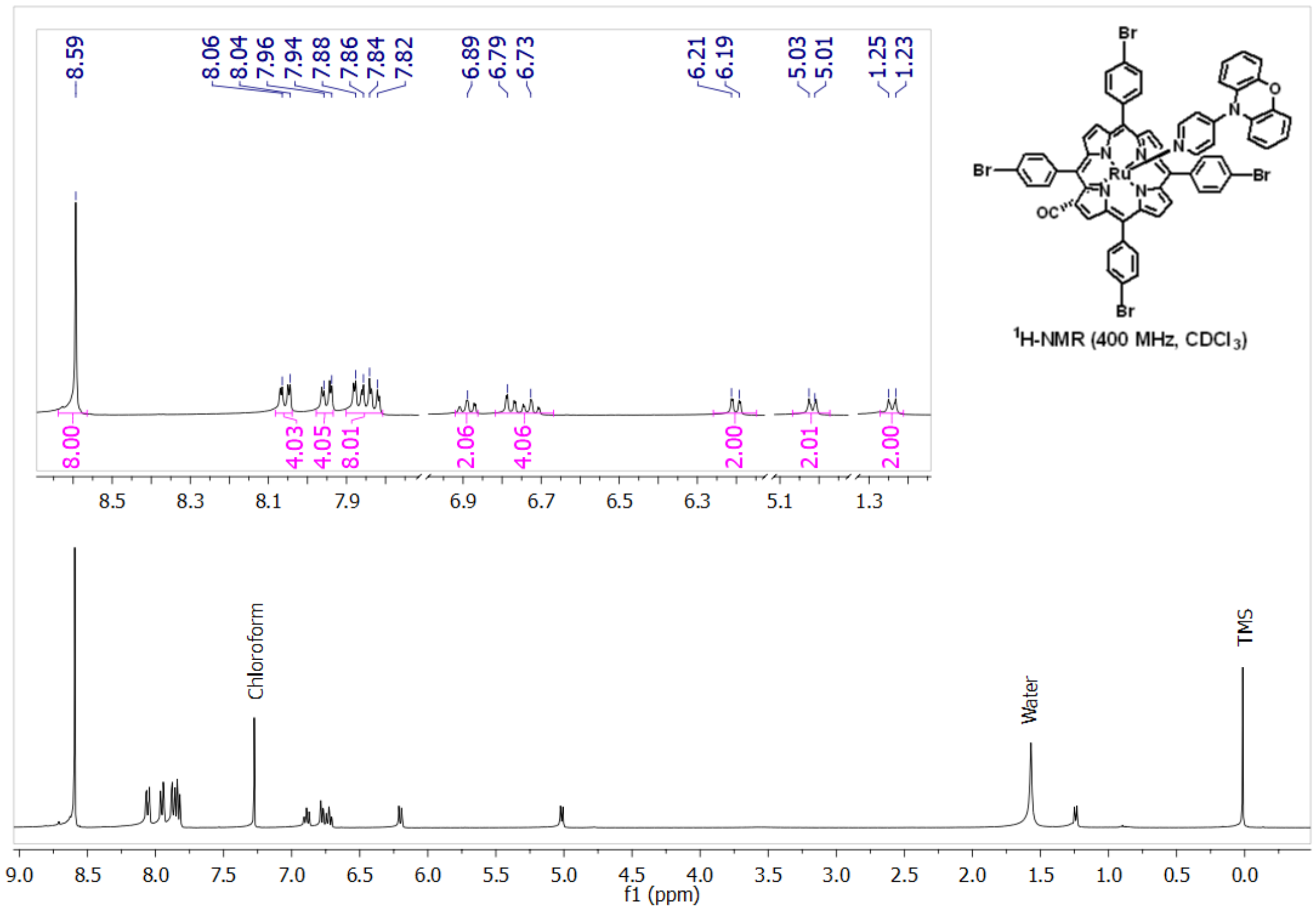

Figure S15. *^{1}H NMR spectrum of 5,10,15,20-tetrakis(4'-bromophenyl)porphine ruthenium(II) (carbonyl)-[10-(pyridin-4-yl)-10H-phenoxazine] (**3B**).*

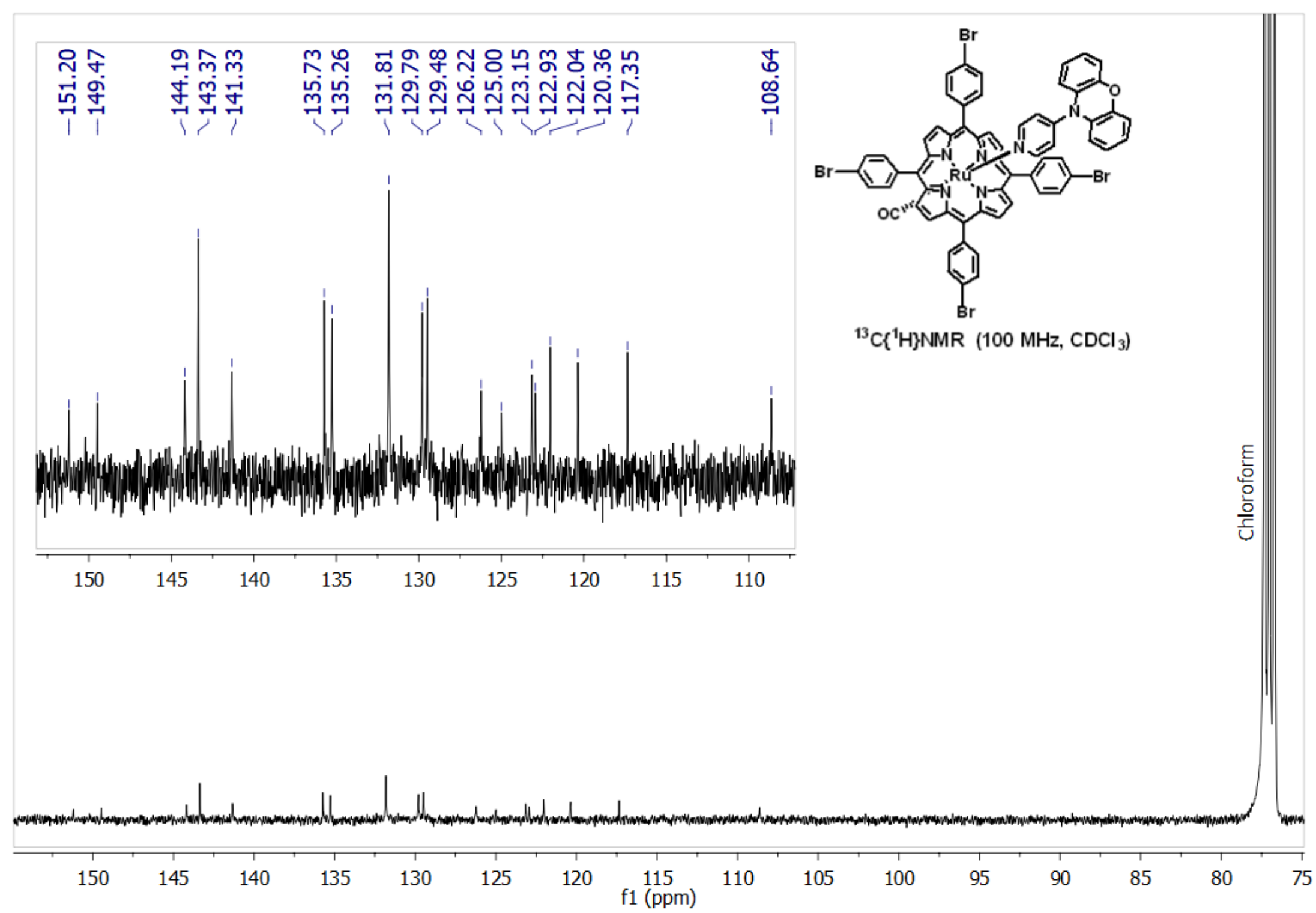

Figure S16. *^{13}C{^{1}H}NMR spectrum of 5,10,15,20-tetrakis(4'-bromophenyl)porphine ruthenium(II) (carbonyl)-[10-(pyridin-4-yl)-10H-phenoxazine] (**3B**).*

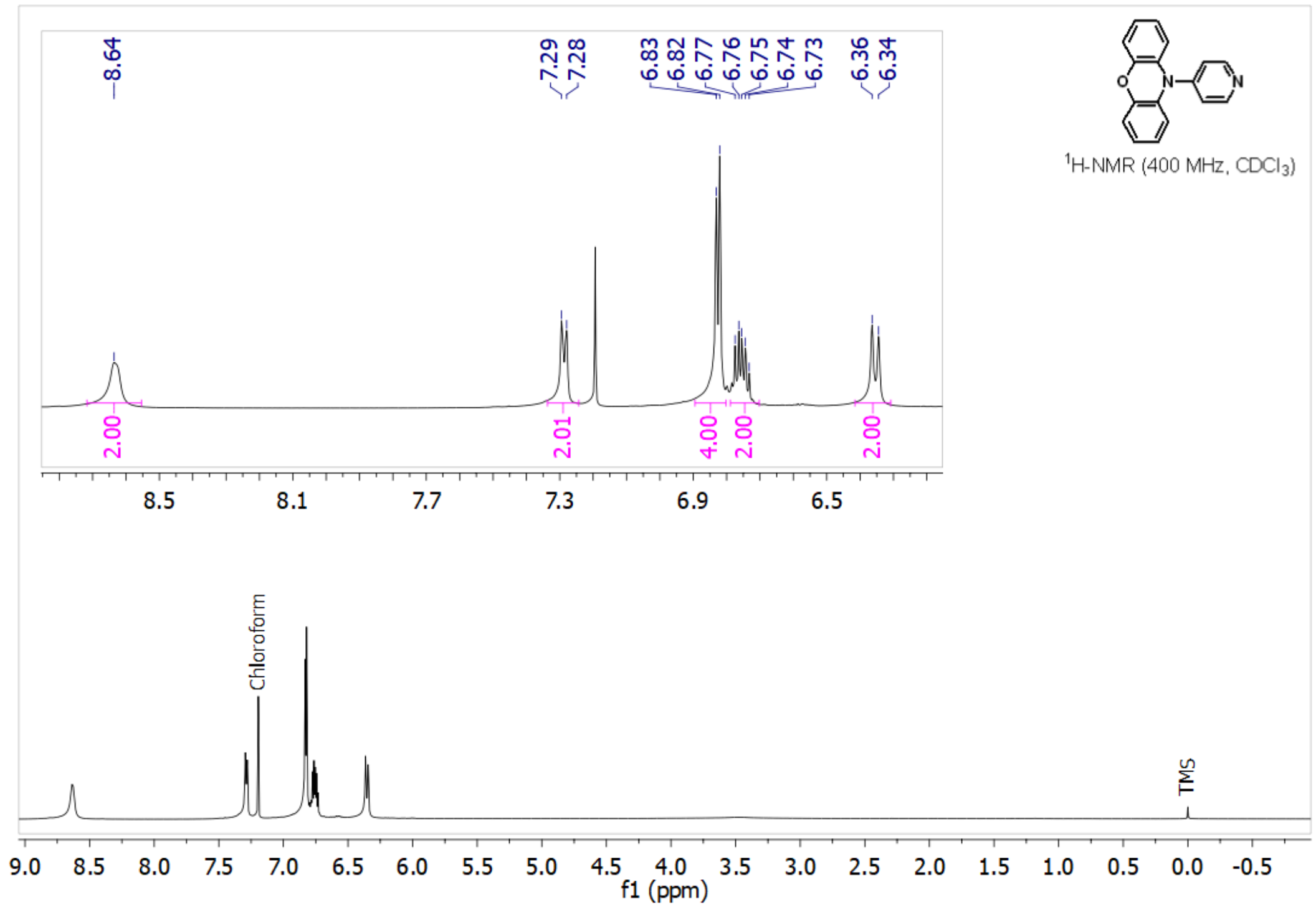

Figure S17. *¹H NMR spectrum of 10-(pyridin-4-yl)-10H-phenoxazine (**PyPX**).*

11. High resolution mass spectrometry spectra of intermediates and final compounds

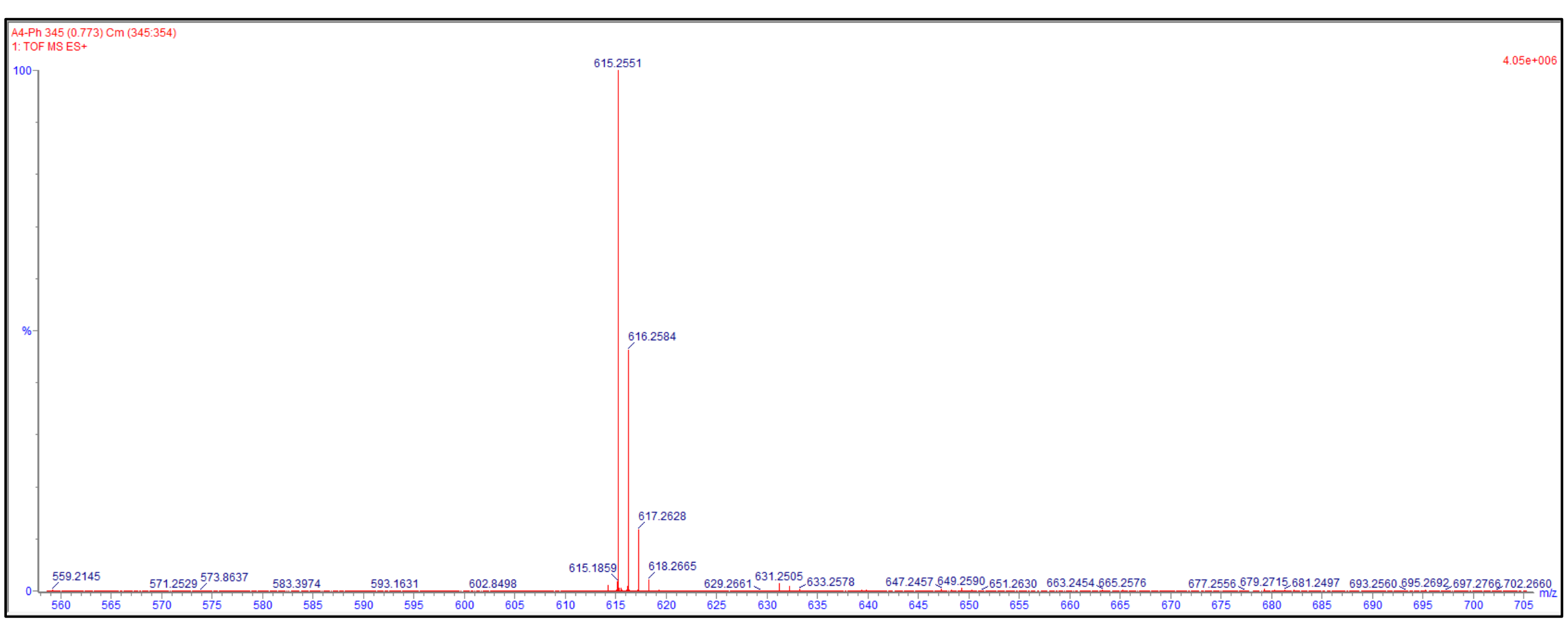

Figure S18. *HRMS (ESI-TOF) of 5,10,15,20-tetraphenylporphyrin (**1A**) observed as [M + H]⁺.*

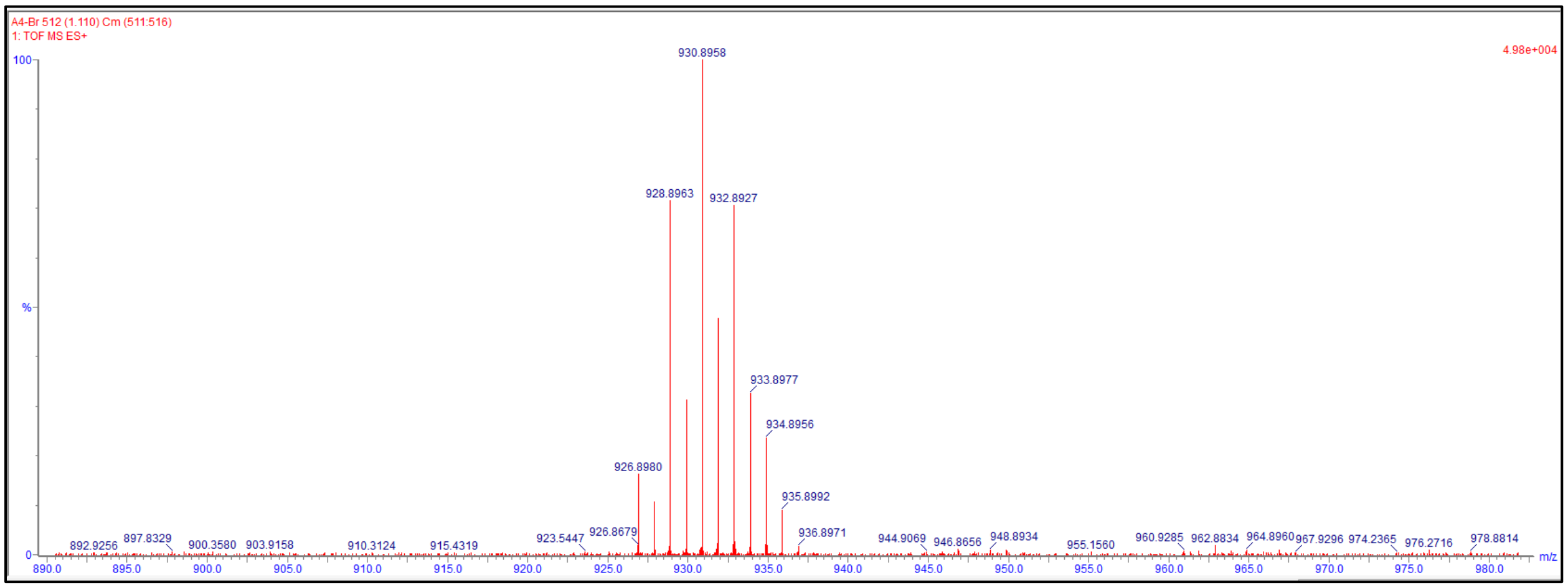

Figure S19. *HRMS (ESI-TOF) of 5,10,15,20-tetrakis(4'-bromophenyl)porphyrin (**1B**) observed as [M + H]⁺.*

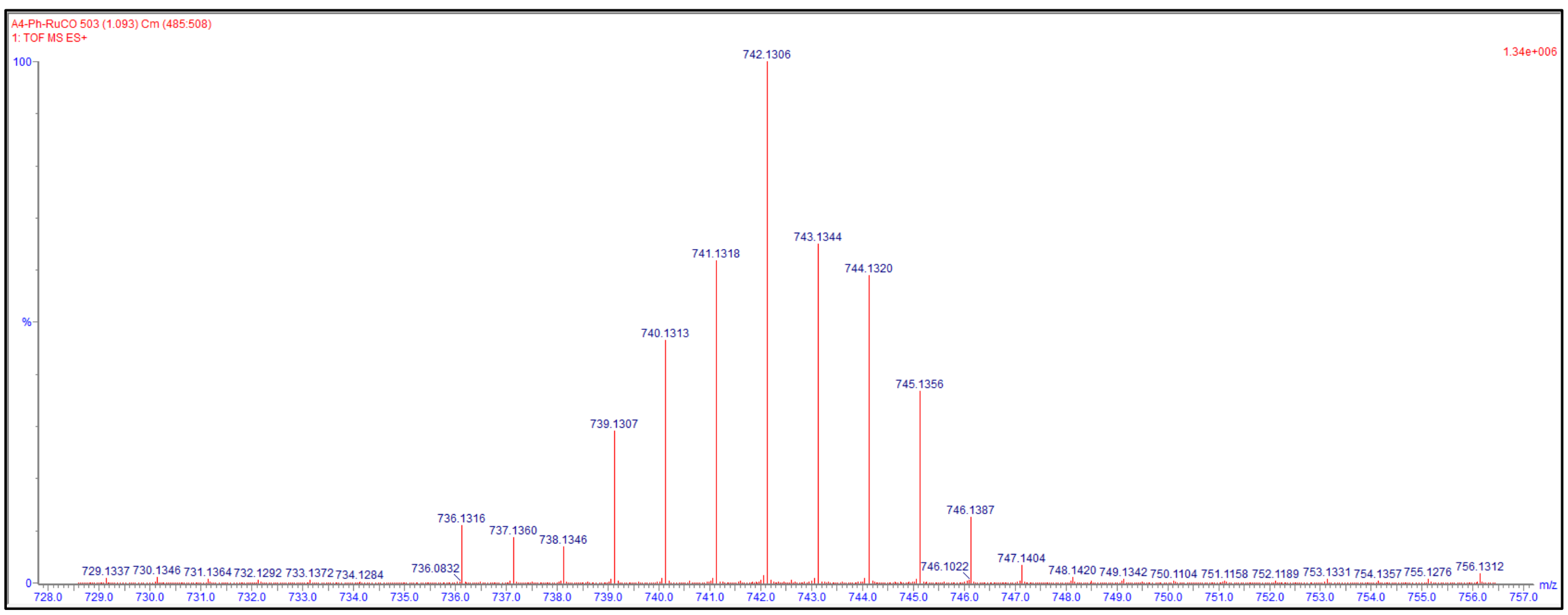

Figure S20. *HRMS (ESI-TOF) of 5,10,15,20-tetraphenyl-21H,23H-porphine ruthenium(II) carbonyl (**2A**) observed as [M]⁺.*

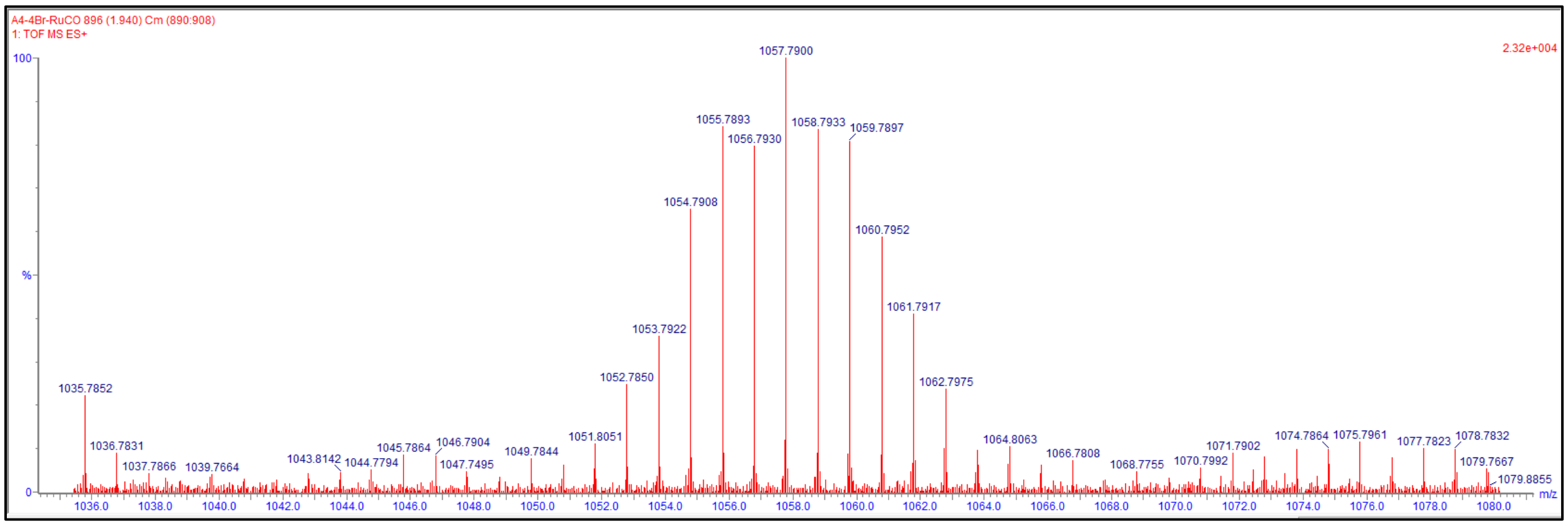

Figure S21. *HRMS (ESI-TOF) of 5,10,15,20-tetrakis(4'-bromophenyl)porphine ruthenium(II) carbonyl (**2B**) observed as [M]$^+$.*

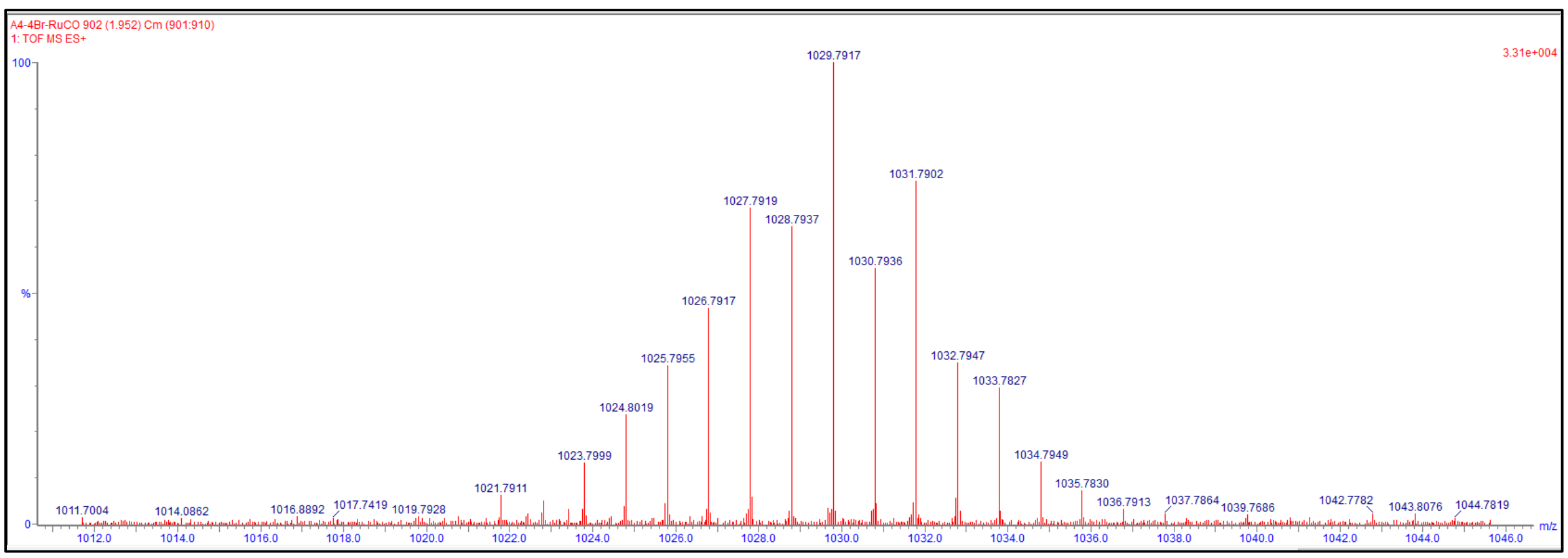

Figure S22. *HRMS (ESI-TOF) of 5,10,15,20-tetrakis(4'-bromophenyl)porphine ruthenium(II) carbonyl (**2B**), with loss of carbonyl axial ligand, observed as [M − CO]$^+$.*

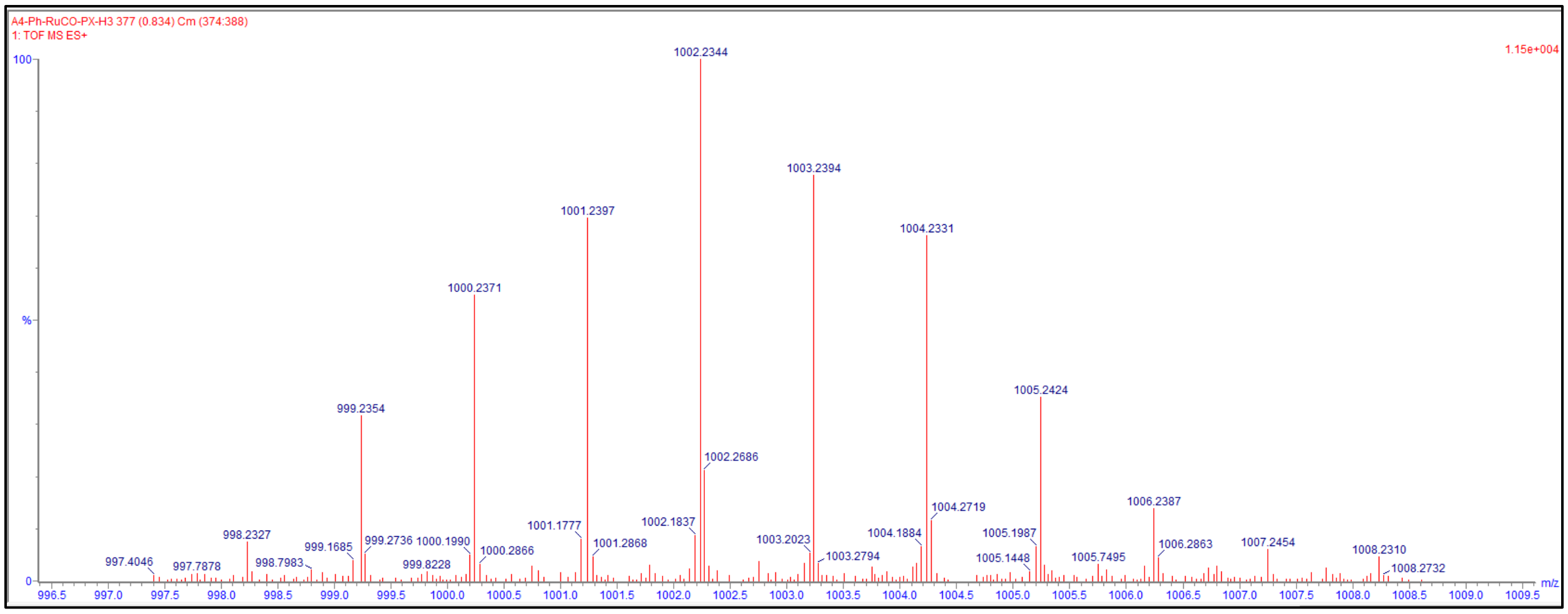

Figure S23. *HRMS (ESI-TOF) of 5,10,15,20-tetraphenyl-21H,23H-porphine ruthenium(II) (carbonyl)-[10-(pyridin-4-yl)-10H-phenoxazine] (**3A**) observed as [M]$^+$.*

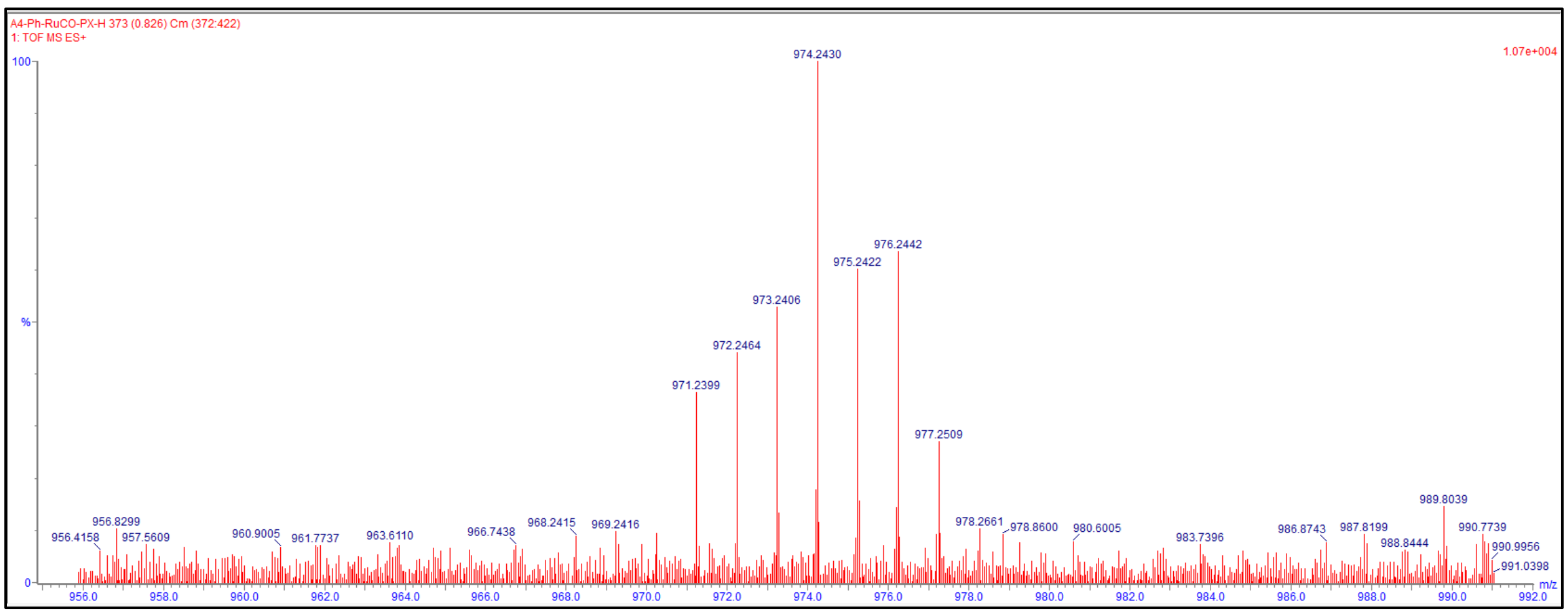

Figure S24. *HRMS (ESI-TOF) of 5,10,15,20-tetraphenyl-21H,23H-porphine ruthenium(II) (carbonyl)-[10-(pyridin-4-yl)-10H-phenoxazine] (**3A**), with loss of carbonyl axial ligand, observed as [M − CO]$^+$.*

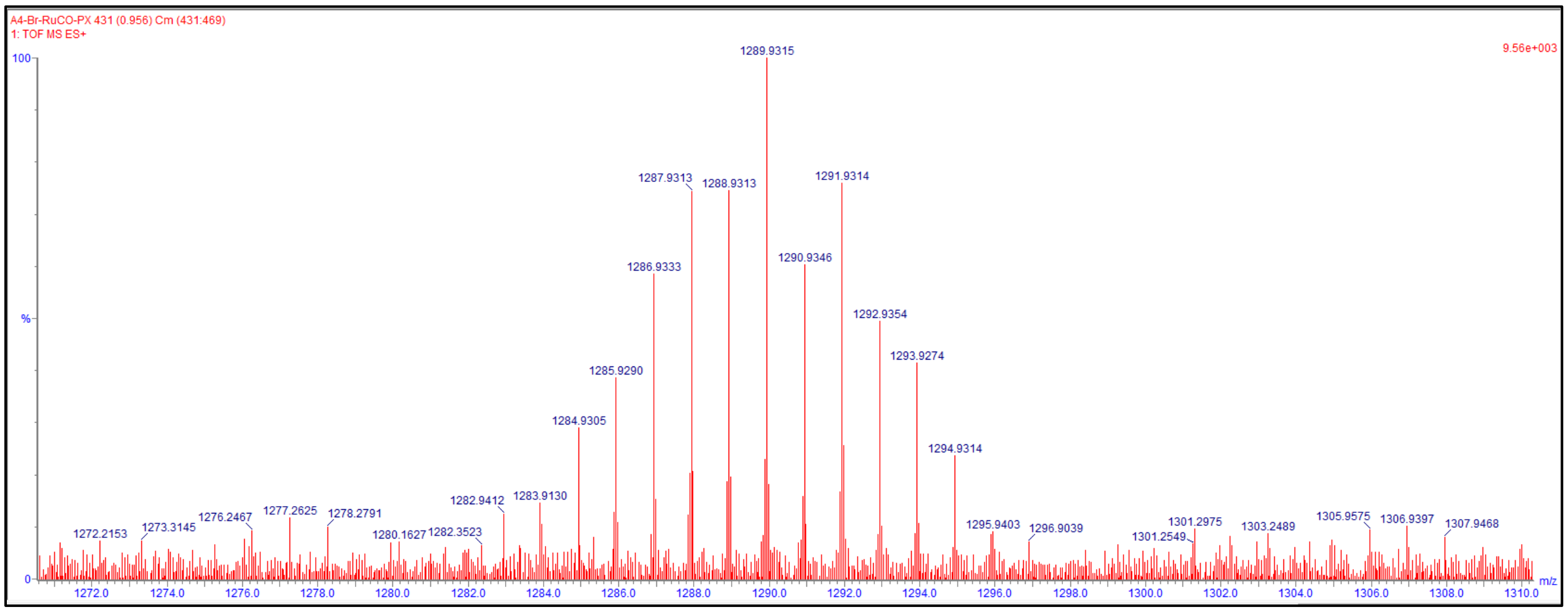

Figure S25. *HRMS (ESI-TOF) of 5,10,15,20-tetrakis(4'-bromophenyl)porphine ruthenium(II) (carbonyl)-[10-(pyridin-4-yl)-10H-phenoxazine] (**3B**), with loss of carbonyl axial ligand, observed as [M − CO]$^+$.*

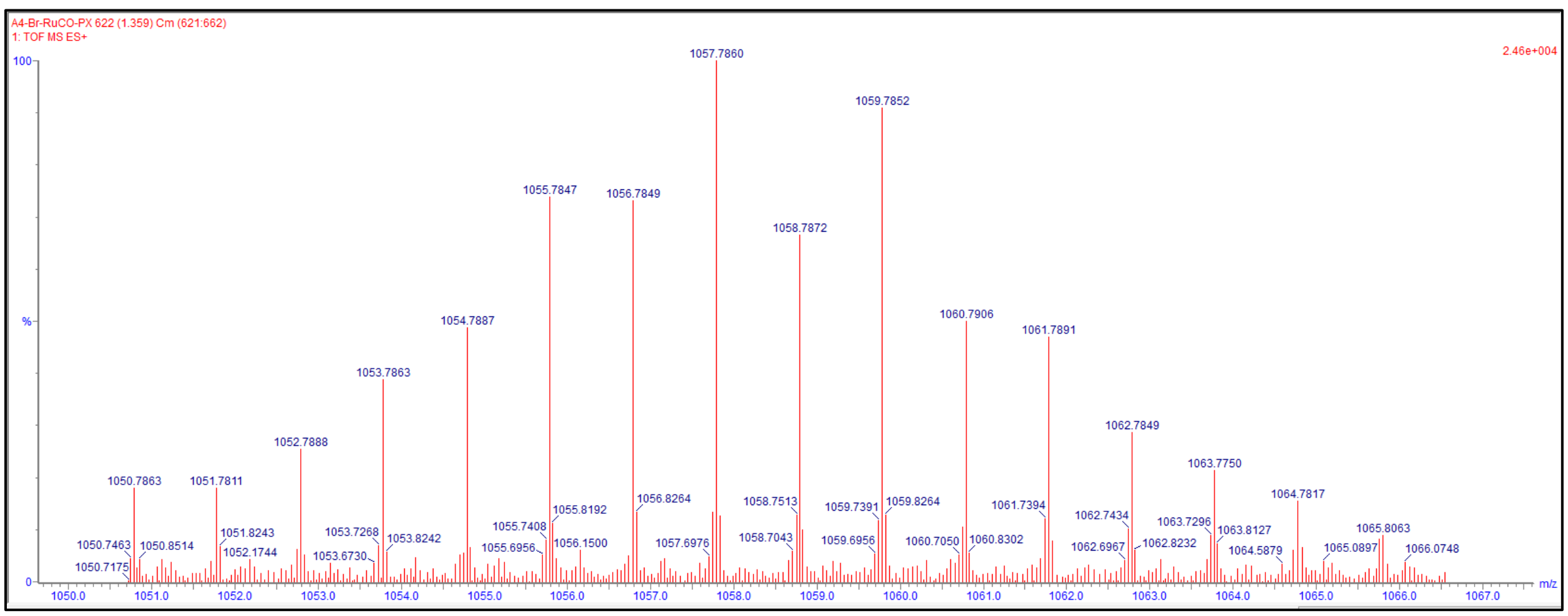

Figure S26. *HRMS (ESI-TOF) of 5,10,15,20-tetrakis(4'-bromophenyl)porphine ruthenium(II) (carbonyl)-[10-(pyridin-4-yl)-10H-phenoxazine] (**3B**), with loss of 10-(pyridin-4-yl)-10H-phenoxazine axial ligand, observed as [M − $C_{17}H_{12}N_2O$]$^+$.*

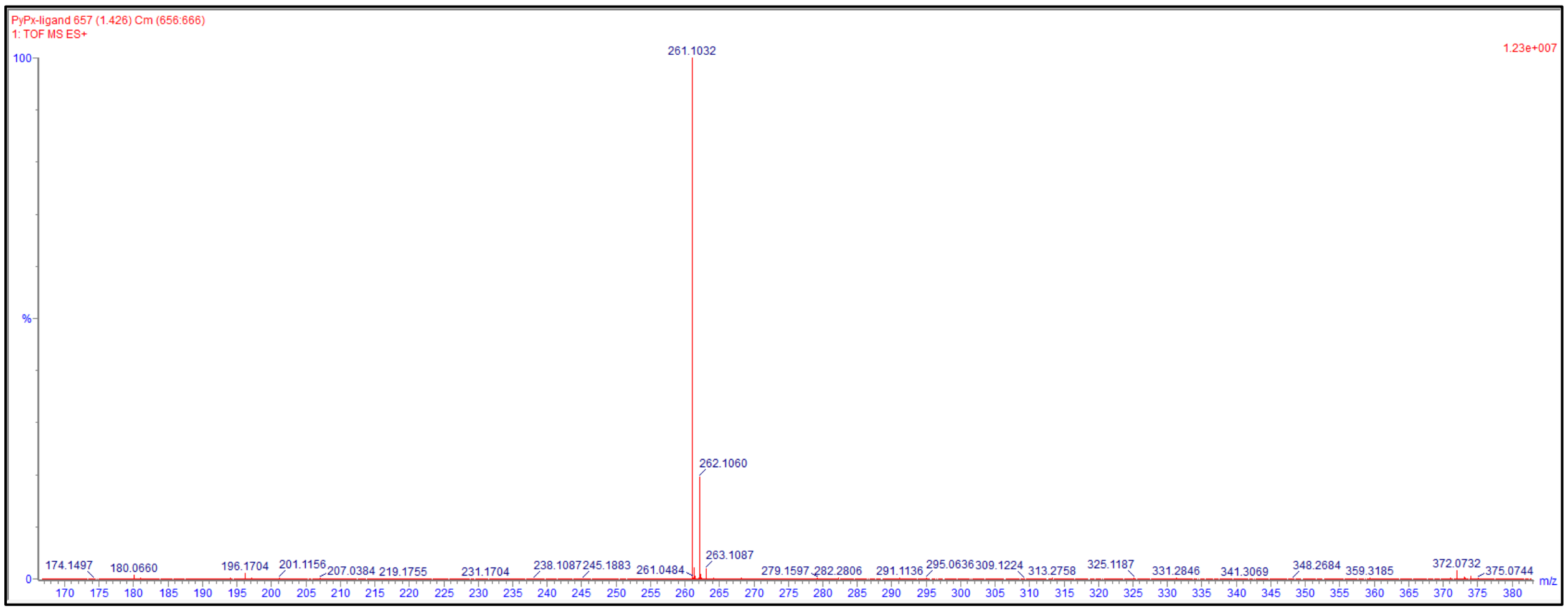

Figure S27. *HRMS (ESI-TOF) of 10-(pyridin-4-yl)-10H-phenoxazine (**PyPX**), observed as [M + H]$^+$.*

12. References

(1) Lindsey, J. S.; Schreiman, I. C.; Hsu, H. C.; Kearney, P. C.; Marguerettaz, A. M. Rothemund and Adler-Longo Reactions Revisited: Synthesis of Tetraphenylporphyrins under Equilibrium Conditions. *J. Org. Chem.* **1987**, *52*, 827–836. https://doi.org/10.1021/jo00381a022

(2) Liu, J.; Yang, X.; Sun, L. Axial Anchoring Designed Silicon-Porphyrin Sensitizers for Efficient Dye-Sensitized Solar Cells. *Chemical Communications* **2013**, *49* (100), 11785–11787. https://doi.org/10.1039/c3cc46581k

(3) Dandia, A.; Chauhan, S. M. S. Synthesis of Meso-Substituted Corroles and Porphyrins Using Iodine as a Catalyst. *Journal of Chemical Sciences* **2020**, *132* (1), 123. https://doi.org/10.1007/s12039-020-01823-z

(4) Vidal, A.; Battistin, F.; Iengo, E.; Milani, B.; Alessio, E. The Insertion of Ruthenium into Porphyrins Revisited and Improved: Proof of Concept Results with a Ruthenium(II) Monocarbonyl Compound, and the Spectacular Effect of Propionic Acid. *Eur. J. Inorg. Chem.* **2019**, *2019* (24), 2883–2890. https://doi.org/10.1002/ejic.201900428

(5) Eaton, S. S.; Fishwild, D. M.; Eaton, G. R. Effect of Para Substituent on Rates of Phenyl Ring Rotation in Gallium Complexes of Para-Substituted Tetraphenylporphyrins. *Inorg. Chem.* **1978**, *17* (6), 1542–1545. https://doi.org/10.1021/ic50184a031

(6) Eaton, S. S.; Eaton, G. R. Phenyl Ring Rotation in Metal Complexes of Tetraphenylporphyrin Derivatives. *J. Chem. Soc. Chem. Commun.* **1974**, 576–577. https://doi.org/10.1039/C39740000576

(7) Weigend, F.; Ahlrichs, R. Balanced Basis Sets of Split Valence, Triple Zeta Valence and Quadruple Zeta Valence Quality for H to Rn: Design and Assessment of Accuracy. *Physical Chemistry Chemical Physics* **2005**, *7* (18), 3297. https://doi.org/10.1039/b508541a

(8) Lenthe, E. van; Baerends, E. J.; Snijders, J. G. Relativistic Regular Two-component Hamiltonians. *J. Chem. Phys.* **1993**, *99* (6), 4597–4610. https://doi.org/10.1063/1.466059

(9) van Lenthe, E.; Baerends, E. J.; Snijders, J. G. Relativistic Total Energy Using Regular Approximations. *J. Chem. Phys.* **1994**, *101* (11), 9783–9792. https://doi.org/10.1063/1.467943

(10) Pantazis, D. A.; Chen, X. Y.; Landis, C. R.; Neese, F. All-Electron Scalar Relativistic Basis Sets for Third-Row Transition Metal Atoms. *J. Chem. Theory Comput.* **2008**, *4* (6), 908–919. https://doi.org/10.1021/ct800047t

(11) Pander, P.; Zaytsev, A. V.; Sil, A.; Baryshnikov, G. V.; Siddique, F.; Williams, J. A. G.; Dias, F. B.; Kozhevnikov, V. N. Thermally Activated Delayed Fluorescence in a Deep Red Dinuclear

Iridium(III) Complex: A Hidden Mechanism for Short Luminescence Lifetimes. *Chem. Sci.* **2023**, *14* (47), 13934–13943. https://doi.org/10.1039/D3SC04450E

(12) Allouche, A.-R. Gabedit-A Graphical User Interface for Computational Chemistry Softwares. *J. Comput. Chem.* **2011**, *32* (1), 174–182. https://doi.org/10.1002/jcc.21600

(13) Roemelt, M.; Maganas, D.; DeBeer, S.; Neese, F. A Combined DFT and Restricted Open-Shell Configuration Interaction Method Including Spin-Orbit Coupling: Application to Transition Metal L-Edge X-Ray Absorption Spectroscopy. *J. Chem. Phys.* **2013**, *138* (20), 204101. https://doi.org/10.1063/1.4804607

(14) de Souza, B.; Farias, G.; Neese, F.; Izsák, R. Predicting Phosphorescence Rates of Light Organic Molecules Using Time-Dependent Density Functional Theory and the Path Integral Approach to Dynamics. *J. Chem. Theory Comput.* **2019**, *15* (3), 1896–1904. https://doi.org/10.1021/acs.jctc.8b00841

(15) Neese, F. The ORCA Program System. *Wiley Interdiscip. Rev. Comput. Mol. Sci.* **2012**, *2* (1), 73–78. https://doi.org/10.1002/wcms.81

(16) Neese, F. Software Update: The ORCA Program System—Version 5.0. *Wiley Interdiscip. Rev. Comput. Mol. Sci.* **2022**, *12* (5). https://doi.org/10.1002/wcms.1606

(17) Becke, A. D. Density-Functional Exchange-Energy Approximation with Correct Asymptotic Behavior. *Phys. Rev. A (Coll. Park).* **1988**, *38* (6), 3098–3100. https://doi.org/10.1103/PhysRevA.38.3098

(18) Weigend, F. Accurate Coulomb-Fitting Basis Sets for H to Rn. *Physical Chemistry Chemical Physics* **2006**, *8* (9), 1057. https://doi.org/10.1039/b515623h

(19) Grimme, S.; Ehrlich, S.; Goerigk, L. Effect of the Damping Function in Dispersion Corrected Density Functional Theory. *J. Comput. Chem.* **2011**, *32* (7), 1456–1465. https://doi.org/10.1002/jcc.21759

(20) Grimme, S.; Antony, J.; Ehrlich, S.; Krieg, H. A Consistent and Accurate Ab Initio Parametrization of Density Functional Dispersion Correction (DFT-D) for the 94 Elements H-Pu. *J. Chem. Phys.* **2010**, *132* (15), 154104. https://doi.org/10.1063/1.3382344

(21) Mori, K.; Goumans, T. P. M.; Van Lenthe, E.; Wang, F. Predicting Phosphorescent Lifetimes and Zero-Field Splitting of Organometallic Complexes with Time-Dependent Density Functional Theory Including Spin-Orbit Coupling. In *Physical Chemistry Chemical Physics*; Royal Society of Chemistry, 2014; Vol. 16, pp 14523–14530. https://doi.org/10.1039/c3cp55438d

(22) Pander, P.; Nastula, D.; Marek-Urban, P. H.; Kozhevnikov, V. N.; Williams, J. A. G. Evidence for Thermally Activated Delayed Fluorescence in Iridium(III) Complexes. *Inorg. Chem. Front.* **2026**, *13* (7), 2816–2834. https://doi.org/10.1039/d5qi01968k

(23) Nyga, A.; Blacha-Grzechnik, A.; Podsiadły, P.; Duda, A.; Kępska, K.; Krzywiecki, M.; Motyka, R.; Janssen, R. A. J.; Data, P. Singlet Oxygen Formation from Photoexcited P3HT:PCBM Films Applied in Oxidation Reactions. *Mater. Adv.* **2022**. https://doi.org/10.1039/d1ma01151k

(24) Nyga, A.; Motyka, R.; Bussetti, G.; Calloni, A.; Sangarashettyhalli, M.; Fijak, S.; Pluczyk-malek, S.; Data, P.; Blacha-grzechnik, A. Electrochemically Deposited Poly (Selenophene) -Fullerene Photoactive Layer : Tuning of the Spectroscopic Properties towards Visible Light-Driven Generation of Singlet Oxygen. *Appl. Surf. Sci.* **2020**, *525* (May), 146594. https://doi.org/10.1016/j.apsusc.2020.146594

(25) Nyga, A.; Czerwińska-Główka, D.; Krzywiecki, M.; Przystaś, W.; Zabłocka-Godlewska, E.; Student, S.; Kwoka, M.; Data, P.; Blacha-Grzechnik, A. Covalent Immobilization of Organic Photosensitizers on the Glass Surface: Toward the Formation of the Light-Activated Antimicrobial Nanocoating. *Materials* **2021**, *14* (11), 3093. https://doi.org/10.3390/ma14113093

(26) Wilkinson, F.; Helman, W. P.; Ross, A. B. Quantum Yields for the Photosensitized Formation of the Lowest Electronically Excited Singlet State of Molecular Oxygen in Solution. *J. Phys. Chem. Ref. Data* **1993**, *22* (1), 113–262. https://doi.org/10.1063/1.555934

(27) Luan, Q.; Hou, L.; Li, H.; Wang, T.; Xu, T.; Ding, Z.; Jiang, J.; Liu, T. Measurement Methods for Triplet-Triplet Annihilation Photon Upconversion. *Optics Continuum* **2024**, *3* (11), 2078–2087. https://doi.org/10.1364/OPTCON.538447

(28) Gray, V.; Dreos, A.; Erhart, P.; Albinsson, B.; Moth-Poulsen, K.; Abrahamsson, M. Loss Channels in Triplet-Triplet Annihilation Photon Upconversion: Importance of Annihilator Singlet and Triplet Surface Shapes. *Physical Chemistry Chemical Physics* **2017**, *19* (17), 10931–10939. https://doi.org/10.1039/c7cp01368j

(29) Zhou, Y.; Castellano, F. N.; Schmidt, T. W.; Hanson, K. On the Quantum Yield of Photon Upconversion via Triplet-Triplet Annihilation. *ACS Energy Letters*. American Chemical Society July 10, 2020, pp 2322–2326. https://doi.org/10.1021/acsenergylett.0c01150
